# Catalytically Active Carbon for Oxygen Reduction Reaction in Energy Conversion: Recent Advances and Future Perspectives

**DOI:** 10.1002/advs.202308040

**Published:** 2024-04-05

**Authors:** Shuling Liu, Ao Wang, Yanyan Liu, Wenshu Zhou, Hao Wen, Huanhuan Zhang, Kang Sun, Shuqi Li, Jingjing Zhou, Yongfeng Wang, Jianchun Jiang, Baojun Li

**Affiliations:** ^1^ College of Chemistry Zhengzhou University 100 Science Road Zhengzhou 450001 P. R. China; ^2^ Institute of Chemical Industry of Forest Products CAF National Engineering Lab for Biomass Chemical Utilization Key and Open Lab on Forest Chemical Engineering SFA 16 Suojinwucun Nanjing 210042 P. R. China; ^3^ College of Science Henan Agricultural University 95 Wenhua Road Zhengzhou 450002 P. R. China; ^4^ Center for Carbon‐based Electronics and Key Laboratory for the Physics and Chemistry of Nanodevices School of Electronics Peking University Beijing 100871 P. R. China

**Keywords:** biomass‐derived catalytically active carbon, electrocatalytic synthesis, energy storage and conversion, hydrogen peroxide, oxygen reduction reaction

## Abstract

The shortage and unevenness of fossil energy sources are affecting the development and progress of human civilization. The technology of efficiently converting material resources into energy for utilization and storage is attracting the attention of researchers. Environmentally friendly biomass materials are a treasure to drive the development of new‐generation energy sources. Electrochemical theory is used to efficiently convert the chemical energy of chemical substances into electrical energy. In recent years, significant progress has been made in the development of green and economical electrocatalysts for oxygen reduction reaction (ORR). Although many reviews have been reported around the application of biomass‐derived catalytically active carbon (CAC) catalysts in ORR, these reviews have only selected a single/partial topic (including synthesis and preparation of catalysts from different sources, structural optimization, or performance enhancement methods based on CAC catalysts, and application of biomass‐derived CACs) for discussion. There is no review that systematically addresses the latest progress in the synthesis, performance enhancement, and applications related to biomass‐derived CAC‐based oxygen reduction electrocatalysts synchronously. This review fills the gap by providing a timely and comprehensive review and summary from the following sections: the exposition of the basic catalytic principles of ORR, the summary of the chemical composition and structural properties of various types of biomass, the analysis of traditional and the latest popular biomass‐derived CAC synthesis methods and optimization strategies, and the summary of the practical applications of biomass‐derived CAC‐based oxidative reduction electrocatalysts. This review provides a comprehensive summary of the latest advances to provide research directions and design ideas for the development of catalyst synthesis/optimization and contributes to the industrialization of biomass‐derived CAC electrocatalysis and electric energy storage.

## Graphical abstract

This review expounds the basic catalytic principles of oxygen reduction reaction, the traditional and advanced synthesis methods and optimization strategies for biomass‐derived catalytically active carbon. The challenges in practical applications of biomass‐derived electrocatalysts and the direction of future development are discussed deeply.

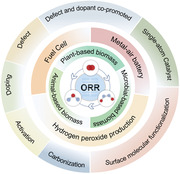

## 1 Introduction

During the exploration to address the shortage of conventional energy (coal, oil, natural gas, etc.),^[^ ^1^, ^2^ ^]^ academia and industry have been dedicated to the design and development of advanced, green, and efficient energy storage and conversion technologies.^[^ ^3^, ^4^, ^5^, ^6^, ^7^ ^]^ Among different energy storage systems, clean and sustainable energy sources such as wind, solar, and hydroelectric power generation cannot meet the demand for long time stable energy supply applications because of the limitation of weather, seasonal, and other environmental factors. Electrochemical energy systems (fuel cells, metal‐air batteries, etc.) are potential research targets for solving current energy and environmental problems because of efficient, green, and stable energy storage and conversion characteristics.^[^ ^8^, ^9^, ^10^ ^]^ The oxygen reduction reaction (ORR) at the cathode of fuel cells and metal‐air batteries suffers from slow kinetics. Highly active catalysts need to be developed to facilitate reaction kinetics for large‐scale commercial production.^[^ ^11^, ^12^ ^]^ Although platinum‐based catalysts exhibit excellent catalytic activity, the scarcity of their source and poor catalytic durability do not meet the needs of large‐scale commercial applications.^[^ ^13^, ^14^, ^15^ ^]^

**Figure 1:**
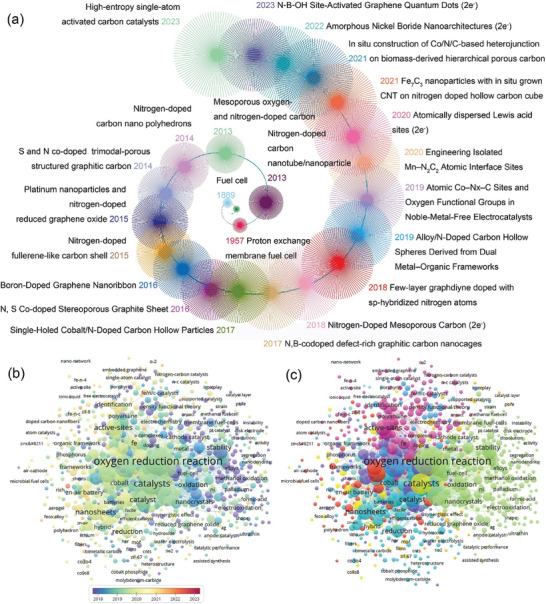
a) Timeline of the development of selected carbon‐based electrocatalytic oxygen reduction catalysts in the last decade. b) Network map of scientific research priorities on ORR in the last 5 years. c) Network diagram of catalyst design and application for ORR.

During the evolution of human civilization, the exploration and application of CAC materials have been used throughout in medicine,^[^ ^16^, ^17^ ^]^ energy,^[^ ^18^, ^19^, ^20^, ^21^ ^]^ environment,^[^ ^22^, ^23^ ^]^ etc. As can be seen from **Figure** **1**a, the development of selected carbon‐based materials in the last decade has built the foundation for ideal ORR catalysts by changing the structural morphology of carbon supports and regulating the doping of non‐precious metals/heteroatoms. When designing and developing new electrode materials, in addition to the improvement of catalytic performance, environmental and economic factors should also be taken into consideration. Abundant renewable raw materials and simple and environmentally friendly preparation methods are the key to develop efficient green catalysts.^[^ ^24^ ^]^

A large number of organisms in nature use carbon as a substrate for the transformation of energy substances. Using biomass as renewable precursors is more efficient, economical, and environmentally friendly than petroleum fuels to produce CAC materials. The former meets the low/non‐fossil energy consumption requirements of a sustainable human society because of its simpler and more controllable features.^[^ ^25^, ^26^, ^27^ ^]^ Based on the different resources, biomass‐derived catalytically active carbon (CAC) materials are summarily classified into plant‐based carbon,^[^ ^28^, ^29^, ^30^, ^31^ ^]^ animal‐based carbon,^[^ ^32^, ^33^, ^34^ ^]^ and microbial‐based carbon.^[^ ^35^, ^36^ ^]^ The porosity of different CAC materials reduces the apparent density for the larger specific surface area. The efficiency of interfacial energy and mass transfer, density of active centers, and diffusion resistance are regulated by controlling the size and shape of the pores of CAC materials.^[^ ^37^, ^38^ ^]^ Meanwhile, biomass‐derived CAC has a variety of nitrogen‐ and oxygen‐containing surface functional groups to serve directly as reactive centers. The pore structure of biomass‐derived CAC materials is adjusted by appropriate activation processes. The active centers can be constructed by heteroatom doping.^[^ ^39^, ^40^ ^]^ Therefore, biomass‐derived CAC as the support of ORR electrocatalysts has become a popular research direction. Based on the network relationship between experimental performance and application of catalysts for ORR in the past 5 years in Figure 1b and the design trend of oxygen reduction catalysts in Figure 1c, it can be seen that the design of CAC‐based catalysts focuses on the preparation of multi‐scale morphology and hierarchical porous structure. The preparation of multifunctional, high catalytic activity biomass‐derived CAC catalysts can follow this idea.

In addition to modulating different preparation methods such as pyrolysis,^[^ ^41^, ^42^, ^43^ ^]^ hydrothermal carbonization,^[^ ^44^, ^45^, ^46^ ^]^ liquefied carbonization,^[^ ^47^, ^48^, ^49^ ^]^ etc., the modification process should also be considered. For one thing, various heteroatoms (e.g., nitrogen (N),^[^ ^50^, ^51^, ^52^ ^]^ phosphorus (P),^[^ ^53^, ^54^ ^]^ sulfur (S),^[^ ^55^, ^56^ ^]^ boron (B),^[^ ^57^, ^58^ ^]^ and fluorine (F),^[^ ^59^ ^]^ etc.) are added to the CAC materials for the promotion of the oxygen adsorption, the reduction of the reaction barriers, and the break of O‐O bonds by adjusting the local charges and spin densities of the carbon substrates to achieve the improvement of catalytic activity. The other is the application of defect engineering.^[^ ^60^, ^61^ ^]^ The adsorption of oxygen‐containing intermediates is favored by defects or disordered structures at the edges or surfaces of CAC materials that break the electron‐hole symmetry. Therefore combining heteroatom doping with defect engineering is a commonly used strategy to enhance the catalytic activity of biomass‐derived CAC electrocatalysts.^[^ ^62^ ^]^ Modified biomass‐derived CAC materials as promising ORR catalysts are widely applied in electrochemical energy storage and conversion systems and green chemistry (including metal‐air batteries^[^ ^63^, ^64^ ^]^ fuel cells,^[^ ^65^ ^]^ and synthetic hydrogen peroxide^[^ ^66^, ^67^, ^68^ ^]^).

After the speedy development of this field, some targeted summaries and comparisons of one or more aspects of biomass‐derived CAC, such as synthetic strategies, performance, and applications in electrochemical energy storage and conversion, have been published. But a systematic and comprehensive review of ORR biomass‐derived CAC‐based catalysts from preparation methods to modulate the structure, structure‐property conformational relationships, and material functionalization for various applications is still in lack.

**Figure 2:**
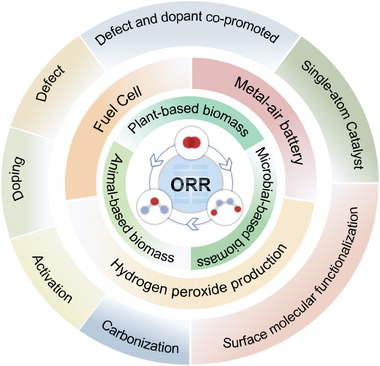
Schematic diagram of the biomass‐derived CAC‐based catalyst precursors, synthesis optimization methods, and applications.

This review talks about 530 literature in the last five years from five aspects such as reaction mechanism, precursors, preparation methods, optimization strategies, and applications as shown in **Figure** **2**. We first briefly outline the reaction mechanism of ORR electrocatalysts. Then the systematically introduction of the preparation of CAC materials from different biomass precursors and a detailed discussion of the strategies to improve the catalytic performance of CAC‐based catalysts. In the end, recent advances in the application of biomass‐derived CAC materials for different reaction pathways of ORR are summarized. The future challenges and major development directions are elucidated. This paper is of great significance in helping new scholars to quickly and easily understand the development process in this field. We believe that a timely review of this important research will be a valuable resource for the advancement of the design and development of green, efficient, and desirable ORR CAC‐based catalysts and for further guidance in the dynamic development of this emerging field.

## 2 Oxygen Reduction Reaction

### 2.1 Mechanism of Oxygen Reduction Reaction and Evaluation of Catalysts

Oxygen reduction reactions are vital nexus for biological and/or chemical energy conversion in nature. Meanwhile, ORR is an important reaction that occurs in the cathode region of various fuel cells or metal–air batteries. An in‐depth study of the mechanism of the ORR is crucial to further promote efficient electrochemical energy storage conversion.^[^ ^69^, ^70^ ^]^ The ORR can be simply generalized as an electrochemical reaction between H_2_O, H_2_O_2_, and O_2_. However, the specifics and number of its experimental steps are nowadays difficult to obtain in real time during the experiments due to the complex and diverse changes of the intermediates under the influence of electrolytes, electrocatalysts, and oxygen coverings. Therefore, the ORR mechanism is very complex.^[^ ^71^, ^72^, ^73^ ^]^ Generally speaking, ORRs are grouped into two alternative pathways: one is the “direct” reduction of molecular oxygen to water, called the four‐electron reduction process, and the other is the conversion of molecular oxygen to hydrogen peroxide and then to water, called the two‐electron reduction process. Regardless of the four‐electron pathway or the two‐electron pathway, the formation of O^2−^ and the next step of the reaction becomes the key to the reaction. This is because the structure of the oxygen molecule determines the pathway of the ORR. By referring to Hund's rule, in comparison to the triplet state, the oxygen molecule in the ground state has one unpaired electron in each of the two extra π* orbitals. The P‐bond electron alignment is two electrons in the bonding orbital and one electron in the antibonding orbital. The bond energy of an oxygen molecule with bond order two is −498.7 kJ mol^−1^, while the bond energy of superoxide ion O^2−^ bond is only −350 kJ mol^−1^. The chemical reduction reaction is closely related to the breaking of the O─O bond.^[^ ^74^, ^75^, ^76^, ^77^, ^78^ ^]^ In this section, the catalytic mechanism of the ORR in the four‐electron path and the two‐electron path will be analyzed according to the different pH values of the electrolyte (acidic, neutral, and alkaline). The electrochemical properties of different biomass‐derived CAC‐based catalysts are similarly summarized.

#### 2.1.1 In Acidic Medium

In acidic media, differences in the oxygen dissociation barriers at the electrocatalyst surface lead to the generation of direct four‐electron pathway ORRs through different mechanisms (dissociation or association mechanisms).^[^ ^4^, ^79^ ^]^ While the indirect four‐electron mechanism (a two‐electron mechanism) involves first a two‐electron pathway for hydrogen peroxide followed by a step of further reduction to water. The association mechanism that can be formulated by the following basic steps (* represents the electrocatalyst surface free sites, (l) and g denote the liquid and gas phases, respectively, and O*, HO*, and HOO* are intermediates for EC adsorption) (Equations (1), (2), (3), (4), (5), (6), (7), (8)):

In the four‐electron path:

(1) $$O_{2}\left(g\right){+}^{*}\to {O_{2}}^{*}$$

(2) $${O_{2}}^{*}+H^{+}+e^{-}\to \mathrm{HO}O^{*}$$

(3) $$\mathrm{HO}O^{*}+H^{+}+e^{-}\to O^{*}+H_{2}O\left(l\right)$$

(4) $$O^{*}+H^{+}+e^{-}\to HO^{*}$$

(5) $$HO^{*}+H^{+}+e^{-}\to H_{2}O\left(l\right){+}^{*}$$

In the two‐electron path:

(6) $$O_{2}\left(g\right){+}^{*}\to {O_{2}}^{*}$$

(7) $${O_{2}}^{*}+H^{+}+e^{-}\to \mathrm{HO}O^{*}$$

(8) $$\mathrm{HO}O^{*}+H^{+}+e^{-}\to H_{2}O_{2}{+}^{*}$$

the dissociation mechanism can be expressed by the following basic steps (Equations (9), (10), (11)):

(9) $$O_{2}\left(g\right)+2^{*}\to 2O^{*}$$

(10) $$2O^{*}+2H^{+}+2e^{-}\to 2HO^{*}$$

(11) $$2HO^{*}+2H^{+}+2e^{-}\to 2H_{2}O\left(l\right)+2^{*}$$

#### 2.1.2 In Neutral/Alkaline Medium

In alkaline media, intermediates such as O, OH^−^, O^2−^, and HOO^−^ are formed. Therefore, the oxygen electrocatalytic reduction reaction in alkaline environments is similarly complex, and not easy to determine the mechanism of the reaction. The association mechanism can be formulated by the following basic steps (Equations (12), (13), (14), (15), (16), (17), (18)):

In the four‐electron path:

(12) $$O_{2}\left(g\right)+*\to {O_{2}}^{*}$$

(13) $$O_{2}*+H_{2}O\left(l\right)+e^{-}\to \mathrm{HO}O^{*}+OH^{-}$$

(14) $$\mathrm{HO}O^{*}+e^{-}\to O^{*}+OH^{-}$$

(15) $$O^{*}+H_{2}O\left(l\right)+e^{-}\to \mathrm{HO}*+OH^{-}$$

(16) $$HO^{*}+e^{-}\to OH^{-}{+}^{*}$$

In the two‐electron path:

(17) $$O_{2}\left(g\right)+H_{2}O\left(l\right)+e^{-}\to \mathrm{HO}O^{*}+OH^{-}$$

(18) $$\mathrm{HO}O^{*}+H_{2}O+e^{-}\to H_{2}O_{2}+OH^{-}$$

the dissociation mechanism can be expressed by the following basic steps (Equations (19), (20), (21)):

(19) $$O_{2}\left(g\right){+}^{*}\to 2O^{*}$$

(20) $$2O^{*}+2H_{2}O\left(l\right)+2e^{-}\to 2HO^{*}+2OH^{-}$$

(21) $$2\mathrm{HO}*+2e^{-}\to 2^{*}+2OH^{-}$$

#### 2.1.3 Evaluation of Oxygen Reduction Electrocatalysts

The Rotating Disc Technology Assisted Electrochemical Workstation allows for tests such as cyclic voltammetry and linear scanning to evaluate the performance of ORR catalysts. For electrochemical systems, the preferred method of study is usually cyclic voltammetry (CV). CV is the most commonly used experimental method for electrochemical transients in the study of electrode reaction kinetics and mechanisms. The corresponding current‐voltage relationship curve (cyclic curve) is scanned and recorded after setting the scan rate and applying the voltage. Linear scanning voltammetry (LSV) is a method of recording the electrolytic current at a working electrode by applying a linearly varying voltage to the electrode. The performance testing of 4e^−^ oxygen reduction electrocatalysts relies on the rotating disk electrode (RDE) technique. A motor is used to control the electrodes in the electrolyte to rotate at an angular velocity (in the range of 50–10 000 rpm) to form a vortex. During electrode rotation, the electrolyte on the electrode surface is tangentially centrifuged while the new electrolyte is re‐injected into the center. The purpose of the high‐speed rotation of the electrode is to obtain a uniform diffusion current because of the uniform thickness of the diffusion layer on the electrode surface. 2e^−^ oxygen reduction electrocatalysts are measured using the rotating ring disk electrode (RRDE) technique. Unlike the RDE technique, RRDE uses a ring‐disk electrode with an additional layer of ring electrodes coaxial to the disk electrode. A thin isolation ring is spaced between the disc electrode and the ring electrode to prevent a short circuit between the ring and the disc. The peroxide generated by the disc electrode during the test is “identified and captured” by the ring electrode due to eddy currents. The amount of H_2_O_2_ formed at the working electrode is therefore quantified by the oxidation current (electrochemical oxidation of H_2_O_2_) at the ring. The percentage content of H_2_O_2_ and the number of electrons transferred are calculated using the following Equations (22) and (23):

(22) $$\%\left(H_{2}O_{2}\right)=200\times \frac{I_{r}/N}{I_{d}+I_{r}/N}$$

(23) $$n=4\times \frac{I_{d}}{I_{d}+I_{r}/N}$$

where *I*_d_ is the disk current, *I*_r_ is the ring current, and *N* is the current collection coefficient of the ring electrode on the rotating ring disk electrode.

Catalysts can change the rate of chemical reaction of reactants without changing chemical equilibrium. Moreover, the quality and physicochemical properties of the catalyst itself is not altered by the chemical reaction. An ideal 4e^−^ ORR catalyst should have good 4e^−^ ORR catalytic activity, good physicochemical stability, near four‐electron selectivity, and good electrical conductivity. Common performance evaluation indexes of 4e^−^ ORR catalysts contain reaction potential (open circuit voltage, OCV), half‐wave potential *E*_1/2_, and limiting current density *j*_L_. The ideal 2e^−^ ORR catalyst is slightly different from the 4e^−^ ORR catalyst. The magnitude of the half‐wave potential is no longer the criterion for 2e^−^ ORR catalyst performance. The test of 2e^−^ ORR catalyst performance focuses on the two‐electron selectivity of the catalyst and the concentration of H_2_O_2_ in the electrolyte (i.e., Faraday Efficiency, FE = *Q*_actual product_/*Q*_theoretical product_). The following table summarizes some of the performances of catalysts prepared using biomass materials as carbon sources for ORRs (4e^−^ ORR catalyst in **Table** **1** and 2e^−^ ORR catalyst in **Table** **2**) in the last 6 years.

**Table 1:** Summary of catalytic performance of biomass‐derived CAC‐based catalysts applied to four‐electron ORRs.

Carbon source	Catalyst	Electrolyte	Onset potential [RHE] and *n*	Half‐wave potential *E*_1/2_	Stability (in J or *E*_1/2_) and duration	Reference
Chitosan	Co‐NCNT	0.1 m KOH	−, 3.25	0.835 V	82.94%, 10h	[80]
Microalgae	N/biocar‐800–7	0.1 m KOH	−, 3.87‐3.98	0.900 V	91.20%, ≈24h	[81]
Chlorophylls	Co‐N‐C/CoO_x_‐800	0.1 m KOH	0.890 V, 3.80–3.90	0.820 V	89.90%, ≈5.5h	[82]
Chitosan	NC‐5‐900	0.1 m KOH	1.017 V, 3.96	0.861 V	87.20%, ≈3.3h	[83]
Legume root nodules	RN350‐Z(1‐2)‐1000	0.1 m KOH	–, 3.63–3.99	0.868 V	92.09%, ≈8.3h	[84]
Wood ear	Co–C‐16	0.1 m KOH	0.917V, 3.30	0.855 V		[85]
Microalgae	malg‐SACs‐900	0.1 m KOH	1.055 V, 3.96–3.99	0.875 V	86.20%, ≈11.1h	[86]
Triarrhena sacchariflora panicles	C‐A‐N	0.1 m KOH	0.940 V, ≈4	0.830 V	94.00%, ≈2.8h	[87]
spirulina	Fe_SA_/FeO_NC_/NSC	0.1 m KOH	0.990 V, ≈4	0.86 V	–	[88]
Starch	Fe_4_N@N–C	0.1 m KOH	–, 3.98–3.99	0.903 V	99.80%, ≈2.8h	[89]
Reed	Si‐N‐C‐6 min	0.1 m KOH	1.000 V, –	0.890 V	–	[90]
Acorn shells	Hemin/NPC‐900	0.1 m KOH	0.990 V, 3.88	0.840 V	85.48%, 10h	[91]
Pomelo peel	PPC‐NaZnFe	0.1 m KOH	–, 3.97	0.860 V	92.50%, ≈2.8h	[92]
Shrimp shell	Cu‐NSDC	0.1 m KOH	0.960 V, 3.90	0.840 V	91.00%, 10h	[93]
Bean sprout	NBSCP	0.1 m KOH	–, 3.56	0.836 V	88.4%, 20h	[94]
Glucose	NPC‐1000	0.1 m KOH	0.925 V, 3.60–4.00	0.859 V	−, 8h	[95]
Flour	E‐FeNC	0.1 m KOH	–, 3.71–3.91	0.875 V	91.40%, 5.6h	[96]
Basswood slice	TARC‐N	0.1 m KOH	0.980 V, 3.89–3.98	0.860 V	73.00%, 24h	[97]
Eggplant	HI‐RGO/CD	0.1 m KOH	0.930 V, 3.57	0.770 V	97.70%, 22h	[98]
Bagasse lignin	LC‐4‐1000	0.1 m KOH	–, 4.12	0.860 V	–	[99]
Soybean roots	Co_3_Fe_7_‐Fe_3_C/HNC	0.1 m KOH	0.980 V, 3.80	0.900 V	–	[100]
Microalgae	MRC	50 mm PBS	0.820 V, 3.80	0.720 V	–	[101]
Wood ear	Co‐C‐16	50 mm PBS	0.715 V, 3.48	0.630 V	–	[85]
Shrimp shell	P/Fe/N‐SS	50 mm PBS	–, 3.87	0.550 V	92.00%	[102]
Corn stalk	C_5_	50 mm PBS	0.850 V, 3.92	0.620 V	38.90%, 0.4h	[103]
Giantarum root	Fe/N/C‐50	50 mm PBS	0.878 V, 3.96	–	–	[104]
Water lettuces	N‐HPCNSs‐800	0.1 m PBS	–, ≈4	0.790 V	91.40%, .3h	[105]
Tea	5%Fe‐N/C	0.1 m PBS	0.820 V,	0.640 V	–	[106]
Coconut palm leaves	Mn‐N‐C‐n	3.5 wt% NaCl	, 3.50	0.564 V	–	[107]
Soybean roots	Co_3_Fe_7_‐Fe_3_C/HNC	3.5 wt% NaCl	0.780 V, 3.70	0.64 V	–	[100]
Legume root nodules	RN350‐Z(1‐2)‐1000	0.1 m HClO_4_	–, 3.90	O.723 V	67.55%, ≈8.3h	[84]
Acorn shells	Hemin/NPC‐900	0.1 m HClO_4_	0.860 V, 3.82	0.720 V	73.00%, 10h	[91]
Lignin	LN‐3‐1	0.1 m HClO_4_	0.892 V, 3.93	0.792 V	–	[108]
Bagasse lignin	LC‐4‐1000	0.1 m HClO_4_	–	0.689 V	–	[99]
Walnut shell	FeCr‐N‐C	0.1 m HClO_4_	0.880 V, ≈4	0.730 V	–	[109]
Soy straw	Fe–N–PC	0.1 m HClO_4_	–, 3.65	0.754 V	93.15%, ≈2h	[110]
Enteromorpha	HT‐950	0.1 m HClO_4_	0.840 V	0.650 V	56.00%, 10h	[111]
Sengon wood	Fe‐Pani‐RGO 2 py	0.1 m HClO_4_	0.820 V, 3.90	0.74 V	–	[112]
Shrimp shell	Fe/N‐SS	0.1 m HClO_4_	0.810 V, ≈4	0.630 V	92.50%	[102]
Pointed tail leaf	NSC‐PT‐0.1	0.5 m H_2_SO_4_	0.800 V, 3.96	0.690 V	–	[113]
Lignin	L_Fe	0.5 m H_2_SO_4_	0.840 V, 3.75	0.770 V	–	[114]
Calvatia	NH_3_‐NPCS‐900	0.5 m H_2_SO_4_	–, 3.97–3.99	0.615 V	–	[115]

**Table 2:** Summary of catalytic performance of biomass‐derived CAC‐based catalysts applied to two‐electron ORRs.

Carbon source	Catalyst	Electrolyte	*n* and H_2_O_2_ selectivity	Faraday efficiency (FE)	H_2_O_2_ production and time of electrolysis	Stability and duration	Reference
Alfalfa	NO/PC‐500	O_2_‐saturated solution	2.3–2.5, 85.1%	–	109.4 mg L^−1^, 4h	–	[116]
Litchi shell	C_800_	0.1 m KOH	2.8, 60%	–	–	92.3%, 1.7 h	[117]
Chitosan	NPCNS_700T	0.1 m KOH	–, 85.4%	80%	223.4 mmol g_catayst_^−1^ h^−1^, 4 h	–	[118]
Sophora flower	MCLA‐TENG	50 mm Na_2_SO_4_	2.04, ‐	–	221.9 mg L^−1^, 2 h	–	[119]
Kapok fiber	KFBC‐700	50 mm Na_2_SO_4_	2.2–2.5, 76.5%	–	10.7 mg L^−1^, 1 h	–	[120]

### 2.2 Research on Catalytic Mechanism

Understanding the catalytic mechanism and the conformational relationship between catalyst electronic configuration and catalytic activity is essential for better‐targeted design and synthesis of ideal catalysts. The modulation of the elemental composition and morphological structure of the catalyst affects the catalytic activity of the catalyst by changing the electronic configuration. Based on the study of catalytic mechanism, the adjustment of catalytic performance of catalysts for target reactions by tuning the elemental composition and microstructure of catalysts on the atomic scale is a feasible approach. Ultimately, the goal of efficiently preparing catalysts with optimal catalytic performance is realized.

#### 2.2.1 Sabatier Principle

**Figure 3:**
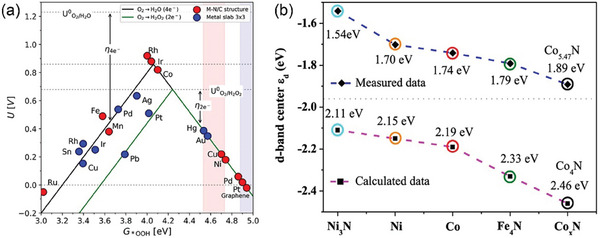
a) Sabatier volcano plots for electrochemical oxygen reduction for closely packed pure metal slabs (in blue) and M‐N/C structures (in red) obtained from DFT calculations.^[^ ^123^ ^]^ Copyright 2018, American Chemical Society. b) summary of all derived d‐band center positions: measured and calculated. The calculated model of Co_5.47_N is based on Co_4_N, due to the structure of Co_5.47_N is almost the same as that of Co_4_N. Dashed lines in g are intended as a guide to the eye.^[^ ^125^ ^]^ Copyright 2021, American Chemical Society.

In heterogeneous catalysis, active sites are adsorption sites for catalytic substrates and intermediates. Therefore, the observation, identification, modification, and design of active sites in heterogeneous catalysis have been of great interest. Sabatier principle is a well‐known theory that describes the relationship between catalytic activity and catalytic active sites. In catalysis, reactant molecules are bonded to the catalyst surface active site, and intermolecular collisions are divided into the following steps. 1) the first molecule collides with the catalyst surface to form a bonded intermediate; 2) the second molecule collides with this intermediate to react, and then proceeds to the subsequent reaction steps. Determination of the binding of the catalytic substrate to the catalytically active site lowers the energy potential barrier because the binding bonds within the catalytic substrate are weaker and easier to break.^[^ ^121^ ^]^ The reaction is favored when the binding energy of the catalyst to the reaction intermediate is moderate (near the top of the volcano diagram). And the adsorption energies of catalytic substrates and intermediates on the active sites on the catalyst surface in the oxygen reduction catalytic reaction satisfy the volcano relationship in **Figure** **3**a. The magnitude of the adsorption energy between the catalyst and the reaction intermediates also relates to the type of pathway followed by the ORR.^[^ ^122^, ^123^ ^]^ However, existing experimental methods cannot quantitatively characterize the strength of the adsorption energy between the catalyst and the corresponding product.^[^ ^124^ ^]^

#### 2.2.2 The D‐Band Model

Transition metals are commonly used as active sites in CAC‐based catalysts. During the catalytic process the surface of the metal forms a chemical bond with the reactant, while the chemical bonds within the reactant are opened to form new bonds.^[^ ^124^ ^]^ The d‐band model is a model that describes the formation of chemical bonds on transition metal surfaces. In this model, the valence electron orbitals of the adsorbate are coupled to the s and d orbital densities of states of the transition metals, resulting in the valence electron orbital densities of the adsorbate shifting and expanding in the direction that favors the adsorbate. This promotes the formation of chemical bonds between the adsorbate and all transition metals (with half‐filled and wider s orbital density of states). As shown in Figure 3b, the energy level positions of the antibonding orbitals are usually higher than those of the d orbitals. The center of the d band relative to the Fermi energy level position can effectively predict the bonding strength between the adsorbent and the metal surface.^[^ ^125^ ^]^ The higher the d orbital position relative to the Fermi energy level position, the higher the antibonding orbital energy level position. The stronger the bonding between the adsorbent and the metal surface will be. For the transition metal system, the degree of occupation of the d orbitals does not change. When the change of the d bandwidth causes the change in the energy level position of the d band center, the change of the energy level of the d orbitals caused by the change of the d bandwidth will be compensated. The change of the d band width with the change of the coordination number of the metal leads to the corresponding change of the position of the energy level of the center of the d band. In this case, the change in the adsorption energy of catalytic substrates and intermediates is equal to the change in the position of the d‐band center energy level.^[^ ^126^ ^]^

The d‐band shapes of active metals are determined by the geometrical structure and elemental composition. The differences in catalytic activity exhibited by catalysts cannot be rationally explained by descriptors such as d‐band centers, d‐band widths, and sp electrons alone. This is because the d‐band shape also has an important influence on the local electronic structure of the adsorbent, such as information on the occupancy state of the adsorbate‐metal antibonding orbitals and the energy level positions.^[^ ^127^ ^]^

The adsorption strength between metal and adsorbate is closely related to its corresponding catalytic activity. Because the d‐band center can regulate the adsorption energy, when the d‐band center is far away from Fermi level, the adsorption energy E_O_ of the intermediate O* species decreases. When the d‐band center approaches Fermi level, E_O_ increases. Therefore, the interaction between metal and adsorbate can be adjusted by adjusting the position of d‐band center of metal catalyst, thus enhancing catalytic activity. The regulation of atomic and electronic structure of electrocatalytic materials can be achieved by alloying,^[^ ^128^ ^]^ building core‐shell structure^[^ ^129^ ^]^ and changing the size of nano‐materials^[^ ^130^ ^]^ to produce lattice mismatch. The stretching or compression of lattice spacing will cause the d‐band center to rise or fall, making it close to or far from Fermi level. When the tensile strain occurs on the metal surface, the energy band of d narrows. In order to keep the d band occupying the same number of electrons, the d‐band center moves upward, thus approaching Fermi level, which may lead to the increase of d with the center energy and the stronger interaction between precious metals and adsorbates. Contrary to tensile strain, when compressive strain occurs, the d‐band center moves down, and the adsorption between precious metals and adsorbed species becomes weak.^[^ ^131^ ^]^ In addition, strategies such as heteroatom doping,^[^ ^132^ ^]^ secondary metal introduction,^[^ ^133^ ^]^ heterogeneous interface construction,^[^ ^134^ ^]^ defects, and geometric electronic structure regulation^[^ ^135^ ^]^ can also fine‐tune the d‐band center.

#### 2.2.3 Limitations of the Existing Catalytic Mechanism

The ultimate performance produced by a catalyst relies on the specific properties of the particular catalyst (intrinsic effects) and the modification of the structure of the catalyst in conjunction with the introduction of other substances (extrinsic effects). All the effects can be attributed to the effects of changes in the electronic configuration and the geometric structure of the catalyst. In order to overcome the theoretical limit of overpotential due to the quantitative relationship of the catalytic activity of a single active site, the design of multiple active sites for different intermediates using different methods (alloying, doping, introduction of defects, support‐catalyst coupling, tuning of nanostructures and structural limiting domains, etc.) is necessary. Different structural surfaces such as pores, holes, cavities, particles, or rods may provide different active sites. Whether it is a change in the local coordination environment or a dissolved portion of intermediates in the electrolyte or additives during the reaction, the three‐dimensional active site effect brought about by altering the surface chemistry of the catalyst can overcome the quantitative scaling relationship.

## 3 Synthetic Sources of Biomass‐Derived Catalytically Active Carbon

Biomass, one of the most fertile and environmentally friendly potential energy sources, can be used as an energy storage precursor for sustainable CAC materials. Biomass materials can be processed to obtain materials or by‐products with multiple functions and structurally stable properties. Biomass covers plants, animals, microorganisms, and their biological excretion and metabolism of organic substances and other categories, and can be converted and accumulated in a short period through the air, water, and light energy, and is a rare renewable resource treasury. Although biomass is a carbon‐rich material, its composition varies depending on its categories, and can usually be summarized as carbohydrates, lignin, proteins, lipids, and others. Even within the same species, the composition and proportions of elements, such as carbon, hydrogen, oxygen, nitrogen, phosphorus, sulfur, and chlorine, contained in different individuals or different organ parts may vary depending on their geographical location, growth conditions, and other factors. Some of the H, O, N, and S in biomass materials can be removed to some extent after processing and reprocessing.^[^ ^136^, ^137^, ^138^ ^]^ Some of these trace mineral elements such as potassium, calcium, sodium, magnesium, and silicon play an unexpected role in the application of biomass‐derived CAC materials for energy storage and conversion applications.^[^ ^139^ ^]^ No matter how the elemental composition of biomass materials changes during processing, their relatively stable three‐dimensional skeleton structure is preserved. Functionalized electrode materials can be synthesized after two high‐temperature pyrolysis steps of carbonization and activation of biomass materials, but the pore structure and graphitization of biomass‐derived CAC materials obtained under different carbonization activation methods or different parameter operations vary. Therefore different pore structures can be tailored to serve in material applications requiring different functions.^[^ ^140^, ^141^, ^142^ ^]^ Consequently, if we want to apply biomass resources on a large scale, we need to achieve a stable carbon yield and a functionalized structure that can be tailored to the application. The selection of widely available and cost‐effective biomass precursors is the primary criterion, and biomass precursors that can be designed to achieve various chemical functions through the structural morphology of CAC materials are the ideal candidates. Notably, when designing and developing biomass‐derived CAC materials, it is not only important to consider tuning out the application‐required pore size structure, but also benchmarking commercial carbon catalysts to partially/completely remove the effect of complex and uncertain elemental compositions left in biomass‐derived CAC materials on the oxygen reduction reaction. This leads to the realization of high‐quality homogeneous biomass‐derived CAC catalysts for large‐scale applications.

The available and various biomass materials can be summarily categorized as plant‐based,^[^ ^143^, ^144^ ^]^ animal‐based,^[^ ^145^ ^]^ and microbial‐based biomass.^[^ ^146^ ^]^ Not only is the elemental and chemical composition of the biomass materials themselves complex and incomprehensible, but it is also equally challenging to predict the final structure and composition of the derived biomass CAC materials obtained after subsequent carbonization and activation reaction treatments.^[^ ^147^, ^148^, ^149^, ^150^ ^]^ It is therefore of utmost importance to find biomass precursors with multidimensional micro‐ or mesoporous structures, multifunctional functional groups, and high carbon yields. In order to find biomass‐derived CAC with both good performance and abundant production, it is important to understand the composition and properties of various precursors, as well as to adapt biomass‐derived CAC to energy storage and conversion applications in the future.

### 3.1 Plant‐Based Biomass and its Chemical Composition

**Figure 4:**
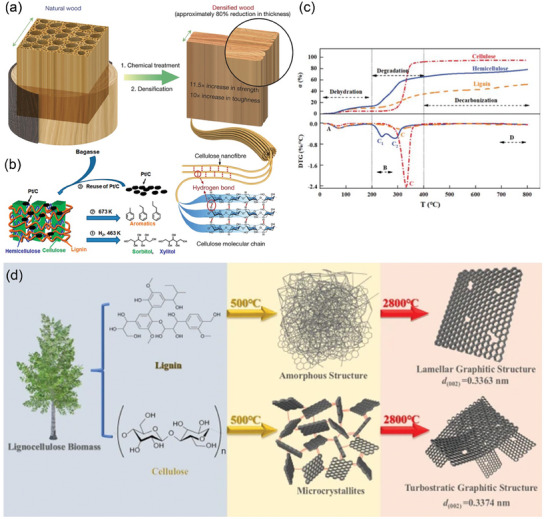
a) Schematic of the top‐down two‐step approach to transforming bulk natural wood directly into super‐strong and tough densified wood.^[^ ^154^ ^]^ Copyright 2018, Macmillan Publishers Limited, part of Springer Nature. All rights reserved. b) Plant biomass pyrolysis carbonization process.^[^ ^165^ ^]^ Copyright 2019, American Chemical Society. c) The α and DTG curves of individual biomass component.^[^ ^166^ ^]^ Copyright 2020, The Korean Institute of Chemical Engineers. d) The Raman spectra of lignin‐2800, points A and B, and cellulose‐2800, points C and D, are shown from the first left to the third right, respectively.^[^ ^172^ ^]^ Copyright 2023, The Author(s).

Green plants use chlorophyll to convert CO_2_ and H_2_O into glucose through photosynthesis while storing energy, and the glucose polymerization reconstitutes starch, cellulose, hemicellulose, and lignin into the plant itself. Thus, from a biological point of view, plant biomass consists mainly of cellulose, hemicellulose, and, lignin, with cellulose and lignin regenerated at a rate of 164 billion tons per year, 15–20 times more than oil production.^[^ ^151^ ^]^ Cellulose molecular chains are formed by the polymerization of (1,4)‐d‐glucopyranose unit chains. And the multiple fiber molecular chains are interwoven and entangled by van der Waals forces, hydrogen bonding forces, and hydrophobic interactions, and finally exist in the form of bundles in the fibers in the plant cell walls.^[^ ^152^, ^153^ ^]^ So they have extremely strong mechanical strength as well as excellent mechanical properties, and their polymerization degrees range from 100–40 000. By choosing different kinds of biomass and different extraction treatments, different kinds of microfibers can be obtained, and even microfibers can be further decomposed into nanofibers.^[^ ^154^ ^]^ As shown in **Figure** **4**a, they are distributed in plant cell walls and used as structural components. Different properties of cellulose can be obtained by changing the OH and H positions at the ends of the carbon atoms on the glucose chain. The preferred orientation arrangement of the crystal structure in cellulose controls the cell wall mechanical properties as well as the cellulose‐cellulose‐ and cellulose‐substrate interactions.^[^ ^154^, ^155^ ^]^ Hemicellulose consists of polysaccharides, such as glucose, mannose, xylose, and arabinose, and belongs to a heterogeneous polysaccharide with a complex structure.^[^ ^156^, ^157^ ^]^ In plant cell walls, hemicellulose chains are tightly bound to the surface of each cellulose microfilament through non‐covalent bonds. At the same time, the unique molecular structure of polysaccharides, the multiphase structure of hydrogels, and the aggregation effect between fibers improve the biomechanical properties of the cell wall in tension and compression, thus allowing the hemicellulose material to be tuned to specific mechanical properties.^[^ ^158^ ^]^ Lignin is a cross‐linked phenolic polymer organic substance with many negatively charged groups. Its complex molecular structure is rich in active functional groups, such as carbonyl, carboxyl, hydroxyl, and aromatic groups, so it is also the largest natural aromatic chemical in nature.^[^ ^159^ ^]^ At the same time, lignin has an extremely strong affinity for high‐valent metal ions, so it can be isolated and extracted in plants by organic solvent methods,^[^ ^160^ ^]^ acid methods,^[^ ^161^ ^]^ and alkali methods^[^ ^162^ ^]^ for the development of useful chemicals. As a filler of plant cell walls, the presence of lignin improves the resistance to compression, enhances the mechanical strength of plants, and facilitates internal water transport.^[^ ^163^ ^]^

As can be seen from Figure 4b, these three main components of biomass are mainly hydrolyzed to (hydroxymethyl) furfural during the pyrolytic carbonization process, then polymerized to form polyfuran, and finally carbonized by further intermolecular dehydration.^[^ ^164^, ^165^ ^]^ After the chemical dehydration of cellulose at 210 °C to become dehydrated fiber with the warming up of pyrolysis, levoglucose, dehydrated sugar, and CO_2_ and H_2_O gaseous volatiles are obtained. Decarboxylation reaction occurs in the primary degradation at low temperature (≤300 °C) to form CO_2_, and the secondary reaction of intermediates increases after the temperature increases and the rate of increase of CO in this process is higher than that of CO_2_. Cellulose has an ordered structure and no branching, so it has better thermal stability than hemicellulose, and most of the pyrolysis products remain unchanged, and only weak bonds are broken to form furan compounds at the reaction temperature. The pyrolysis process of both occurs mainly at 200–350 and 290–400 °C, and the gaseous volatiles are enhanced with increasing temperature, favoring the formation of more porous structures. Compared to cellulose and hemicellulose, lignin consists of a very complex polymer of different types of bonded phenyl propane units, and because of the complex composition, this composition is relatively stable during the pyrolytic carbonization at 140–900 °C, with very few intermediates converted to final participants and a much higher residual carbon weight percentage (≈40 wt%) than other carbons. All the above information can be obtained from Figure 4c.^[^ ^166^ ^]^ Therefore, lignin becomes an ideal material for the preparation of carbon fibers, again, its complex molecular weight and functional groups predestine the mechanical properties of lignin‐based carbon fibers to be much less than those of commercial carbon fibers.^[^ ^167^, ^168^ ^]^

Hemicellulose has negligible solid products after pyrolysis at 500 °C. Cellulose and lignin, on the other hand, have different structural changes after ultrahigh‐temperature graphitization (pyrolysis at 2800 °C) due to significant structural differences. The char formed by cellulose pyrolysis at 500 °C contains a large number of C─C bonds due to the abundant amorphous carbon fractions of the microcrystals connected by chemical bonds. These amorphous components lead to chaotic arrangement of the microcrystals resulting in the difficulty of obtaining a continuous and regular lamellar structure even after the subsequent ultrahigh‐temperature graphitization treatment.^[^ ^169^, ^170^ ^]^ The pyrolysis temperature changes the final disorder degree of carbon materials by affecting the absence/existence of medium‐range order. Low‐temperature pyrolysis forms smaller and denser microcrystalline carbon. The increase in temperature will embed highly distorted nanocrystallites in the continuous random network, which will increase the sheet resistance of the material by 10^9^ times and make it more electrically insulating.^[^ ^171^ ^]^ Therefore, lignin retained part of the stable carbon skeleton after the coking treatment (pyrolysis at 500 °C), which was favorable for the reconstruction and formation of a continuous graphite zone. The char of cellulose was transferred to tiny massive interwoven graphitic microcrystals in high concentration with limited reconstruction to form a vortex layer structure (Figure 4d). The well‐organized microcrystals in the coke advance the graphitization to build tiny crystal domains with higher structural regularity. Chaotically arranged coke microcrystals do not extend well during graphitization and it is difficult to improve the overall crystallinity. The degree of graphitization of lignin under calcination at 2800 °C was 89.53%. The continuous lamellar structure with high crystallinity had fewer structural defects and a conductivity of 104.6 S cm^−1^ under 20 MPa. The degree of graphitization of cellulose was 76.74% under the same treatment and had more structural defects, and the conductivity decreased to 48.8 S cm^−1^ under 20 MPa.^[^ ^172^ ^]^

#### 3.1.1 Stems/Straw/Rhizomes of Plants

Plants absorb water and salts through their roots and transport them to various locations through stalks/stems. Similarly, the organic matter accumulated by leaves after photosynthesis is transported through rods/stems to other organs for storage. Therefore, the rods, stems, and roots of plants have a higher ionic content and a greater percentage of lignocellulose compared to flowers, leaves, fruits, and seeds.

**Figure 5:**
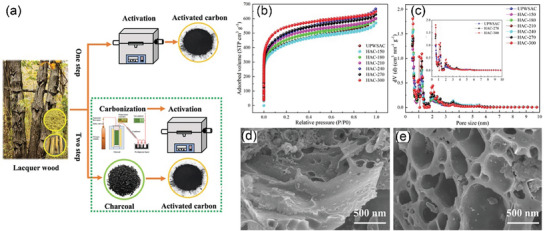
a) Preparation of CAC from lacquer wood by two‐step activation^[^ ^173^ ^]^ Copyright 2021 Elsevier Ltd. All rights reserved. b) Nitrogen adsorption/desorption isotherms of UPWSAC and HAC‐X; c) Pore size distribution; SEM images of d) UPWSAC and e) HAC‐300.^[^ ^174^ ^]^ Copyright 2022 Elsevier B.V. All rights reserved.

After continuous cutting of the bark to obtain raw lacquer, we can discard the traditional treatment (discarding it or burning it) and instead use it as a biomass material to prepare activated carbon, enriching its economic value. As shown in **Figure** **5**a, charcoal, charred wood liquor, and gas were generated after lacquered wood pyrolysis, but the char material obtained at this time had some cavities filled with white tar material. Activation by impregnation with H_3_PO_4_ solution can be performed to form surface oxygen functional groups, to improve graphitization in the subsequent high‐temperature carbonization process, together with the porous structure, to form well‐developed laminar pores to enhance ion transport and further improve the electrochemical properties of the char material. In the two‐step activation process, not only the CAC is obtained, but also the charred wood liquid, combustible gas, and charcoal produced during the reaction are collected, further improving the comprehensive utilization of lacquered wood. The CAC prepared by the one‐step activation method had a more excellent specific surface area (*S*_BET_ = 1609.09 m^2^ g^−1^) and larger specific capacitance (354 F g^−1^).^[^ ^173^ ^]^

Straw becomes a more abundant source of biomass than wood obtained from various types of trees as biomass‐derived CAC precursors. Wheat straw was hydrothermally treated as a biomass precursor and further dehydrated and polymerized after partial degradation of lignocellulose at high temperature, redistributed with lignin to form carbon microspheres with increased specific surface area (2034.41 m^2^ g^−1^) and consequently increased effective contact area with activator and more stable overall structure. The subsequently activated carbonization will obtain more developed and uniformly distributed carbon defects and pore size structure as shown in Figure 5b–e, the whole hydrothermal treatment process through the Maillard and Mannich reaction of lignocellulose, the nitrogen‐containing substances retained in the hydrocarbon, and the β‐O‐4 bond breakage of lignin to produce more phenolic hydroxyl groups, these are for the subsequent preparation of high specific capacitance of CAC (286.95 F g^−1^).^[^ ^174^ ^]^

The world's fourth largest crop, the potato, is also the most familiar tuber plant. Wang et al. used the potato as a precursor to prepare CAC using a self‐activating method to prepare CAC, where the potato releases CO_2_ and H_2_O in the early stages of the heating process, resulting in mass loss. When the temperature reached (250–600 °C), the organic structure of potatoes was cleaved and compounded, and CO_2_ and H_2_O acted as activators for the whole cleavage and compounding reaction. Being in the rhizome site, the metals (K, Na, Ca) within the biomass also act as catalytic centers to assist in reducing the activation energy between the activator and carbon, resulting in higher porosity compared to physical activation alone.^[^ ^175^ ^]^

#### 3.1.2 Leaves/Flowers/Fruits of Plants

Unlike straw, tree trunks, and root systems, flowers, leaves, and fruits are softer biomass materials with a higher sugar and protein content in their composition. Fruits, in particular, can have a protein and lipid content of 6%‐45%. And these three parts of tissues and organs contain processes such as the occurrence of photosynthesis the and production/storage of nutrients, so the nitrogen, calcium, and magnesium in the corresponding CAC materials prepared will become surface functional groups/active centers to improve the electrochemical performance. And the whole pyrolytic carbonization process, in addition to understanding the changes of lignocellulose with increasing temperature, the pyrolytic reactions of lipids and proteins also influence the structural composition of CAC materials.

**Figure 6:**
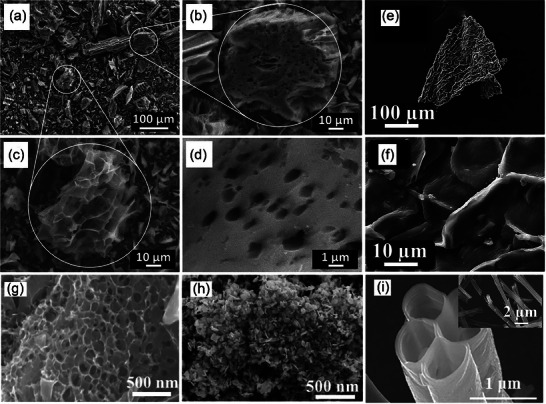
SEM images of NAB800‐3 obtained at a) 100 µm, b,c) 10 µm, and d) 1 µm.^[^ ^177^ ^]^ Copyright 2020 Elsevier B.V. All rights reserved. FE‐SEM images of e, f) carbonized cherry flower (CFC),^[^ ^178^ ^]^ Copyright 2022 Elsevier Ltd. All rights reserved. g) high magnification SEM images of f‐DSAC, h) SEM micrographic image of MoS_2_,^[^ ^184^ ^]^ Copyright 2020 Elsevier B.V. All rights reserved. i) and the inset SEM images of the ACTBs.^[^ ^185^ ^]^ Copyright 2018 Elsevier Ltd. All rights reserved.

For households and food processing plants, pineapple crown leaves have been disposed of as waste, but magnetized CAC can be successfully prepared for dye removal using KOH activation in combination with microwave heating. Throughout the reaction, the diffusion of potassium compounds broadens the carbon matrix pores and reacts with some of the carbon to form new pores for subsequent loading of magnetite. The micro/mesopores of the CAC material containing oxygen functional groups and the magnetic adsorbent removed the dye from the contaminated water very well.^[^ ^176^ ^]^ The use of KOH as an activator and pyrolytic carbonization allows the treatment of biomass waste such as pineapple crown leaves in addition to turning invasive vegetation (crocodile grass) into a treasure. As analyzed above, the escape of volatiles during the reaction formed pores of different shapes/sizes in **Figure** **6**a–d, making the carbon matrix highly porous. This disordered thin porous structure has a very high surface area (3106 m^2^ g^−1^,) and pore volume (1.62 cm^3^ g^−1^), and facilitates the diffusion of gases. Therefore, the low cost and simple treatment means of alligator weed make it an ideal material for biomass carbon precursors.^[^ ^177^ ^]^

Whenever the petals wilt, except for a very small portion for the development of color cosmetics products, most of the flowers are treated as waste. So taking the flowers to prepare biomass‐derived CAC becomes a good choice. Taking cherry blossoms as an example, after KOH activation and high‐temperature carbonization, the external morphology of carbonized cherry blossoms does not change much, but there are many folds on their surface and hollow structures as in Figure 6e,f. The presence of all these enhances the flow of ions and provides additional area for charge storage. The rich content of nitrogen functional groups in bioactive carbon produces defects that provide an active surface for ions and the incident free electrons increase the electrical conductivity. The synergistic effect of both improves the electrochemical properties of the CAC material with a specific capacitance of 333.8 F g^−1^.^[^ ^178^ ^]^ In addition to being taken from flower petals, flower pollen can also be used to prepare CAC. The carbon products obtained by high‐temperature pyrolytic carbonization after ZnCl_2_ activation using *Butnea monosperma* pollen as a precursor were a few microns in size. The carbon structure of mesopores and micropores formed by interconnecting the flakes was controlled by adjusting the impregnation ratio. The interconnected porous structure solves the problem of migration and adsorption of electrolyte ions, resulting in good electrochemical performance of the catalyst in water‐based and ionic liquid‐based electrolytes.^[^ ^179^ ^]^

Normally, the edible part of the fruit is removed and the rest, including the peel and seeds, is disposed of as waste. Seeds will be discussed separately in the next subsection, and the focus of this subsection is on the recycling of peels. The peel of citrus fruits is simple and easy to obtain,^[^ ^180^, ^181^, ^182^, ^183^ ^]^ and the heat treatment method can be changed to promote the activation cracking reaction of biomass by high‐speed heating throughout the preparation process, even better removing volatiles and thus better serving the pore development of CAC materials.^[^ ^180^ ^]^ Or choose different activators and other chemicals to activate and modify the material, the final treatment to obtain CAC materials with an ordered arrangement of dense porous structure, and the introduction of other heteroatoms not only introduces active sites but also may lead to a large number of defects induced.^[^ ^181^ ^]^ Regardless of the efforts made, obtaining a highly uniform and dense pore structure will provide a high surface area and more active sites in subsequent applications, and this CAC material is excellent for gas adsorption or water pollution decontamination applications.

#### 3.1.3 Seeds of Plants

The seeds of various types of plants in nature have different forms, and some plants have evolved their seed shells to be woody in order to avoid being eaten and digested by animals, For example, date seeds,^[^ ^184^, ^186^ ^]^ The apricot (*Prunus armeniaca* L.) seed,^[^ ^187^ ^]^ *Tucumã* seed,^[^ ^188^ ^]^ and tangerine seed.^[^ ^189^ ^]^ The date seeds were thermally activated by pyrolysis followed by thermochemical activation using KOH, a process that induces oxidation of surface carbon atoms to obtain highly mesoporous carbon in Figure 6g,h. This high‐density pore structure facilitates the anchoring of MoS_2_, and the functionalized CAC material is tailored by chemical reaction and ultrasonic treatment to uniformly distribute clusters of MoS_2_ nanosheets over the whole carbon matrix in Figure 6f. The final catalyst obtained has a high specific surface area (885 m^2^ g^−1^) and a uniform pore distribution (15‐80 nm), as this is favorable for CO_2_ adsorption during hydrogenation.^[^ ^184^ ^]^ Compared with direct pyrolysis and carbonization of date palm seeds to produce CAC, apricot seeds can be first de‐oiled by KOH‐catalyzed methanol and ethanol esterification reaction to produce methyl and ethyl biodiesel, followed by NaOH as an activator and then pyrolysis and carbonization to obtain the target product CAC. Through the activation of NaOH, the CAC has a richer pore structure and the presence of surface functional groups is more favorable for the adsorption of pollutants. Notably, apricot kernel oil is a good potential feedstock for biodiesel, with a higher flash point than petroleum diesel, making it safer for transportation and increasing the overall economic value of apricots.^[^ ^187^ ^]^ Interestingly, some plant seeds were spherical in whole when untreated, while the CAC materials obtained after activation and carbonization were still spherical in shape and had high mechanical strength and good adsorption properties. This provides a possibility to explore the preparation of spherical CAC.^[^ ^190^ ^]^

There is another kind of seeds with a more special form, showing a flocculent shape and using the wind for seeding purposes, such as willow flocc,^[^ ^191^, ^192^ ^]^ poplar floc,^[^ ^193^ ^]^ dandelion,^[^ ^185^ ^]^ etc. Most of the CACs prepared with such materials as biomass precursors show a hollow carbon microtubule structure in Figure 6i with excellent layered porosity. For the willow impregnation pretreatment using Ni(NO_3_)_2_·6H_2_O, the carbon matrix obtained by pyrolytic carbonization after activation with KOH as an activator retains a hollow microtubular structure on the rough surface, while the gas release during the reaction process makes nanopores appear on the tube walls, forming a nano grid structure. These qualities greatly provide the convenience of rapid electron transfer, and at the same time, help electrolyte ions to diffuse into the pores greatly improving the electrochemical properties of the CAC material. Therefore, willow‐based CAC is an ideal electrode material for capacitors and full batteries.^[^ ^191^ ^]^

### 3.2 Animal‐Based Biomass and Its Chemical Composition

The uncountable number of animals on earth also provide a constant supply of biomass materials, and the main components used in animal‐based biomass‐derived CAC contain chitin and protein.

**Figure 7:**
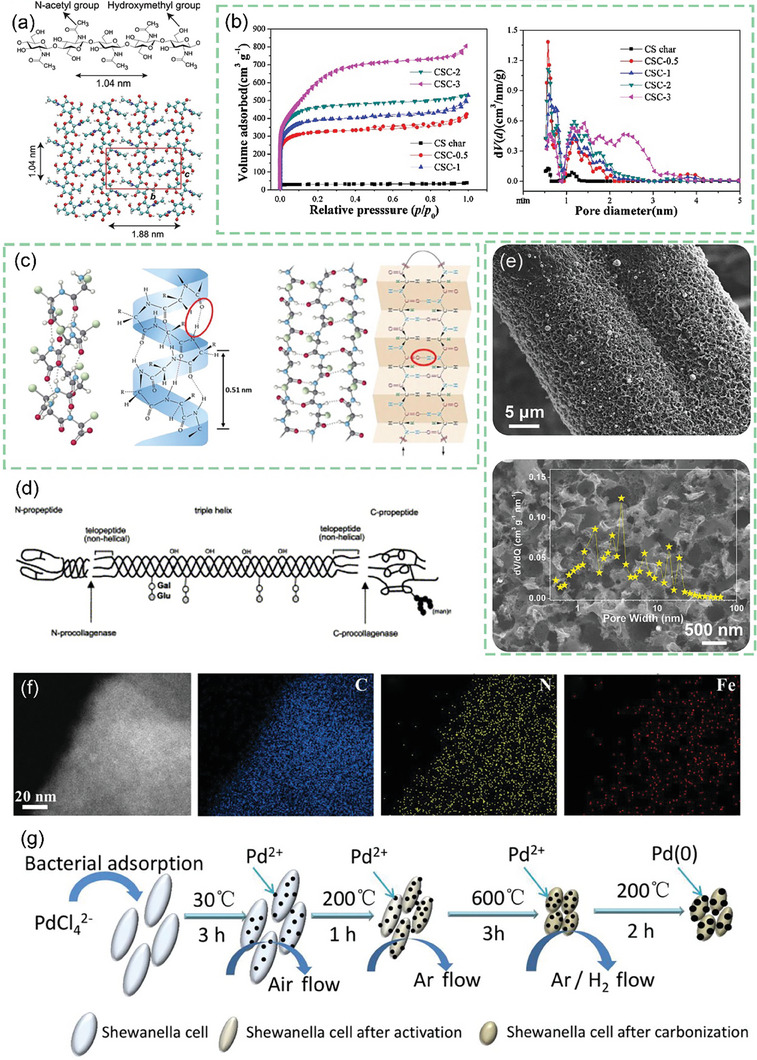
a) Chemical structure and nanocrystal structure of chitin.^[^ ^201^ ^]^ Copyright 2022 The Authors. Small Methods published by Wiley‐VCH GmbH. b) Nitrogen adsorption/desorption isotherms and corresponding pore size distribution curves using the NLDFT model of cicada slough‐derived carbons.^[^ ^210^ ^]^ Copyright 2017 Elsevier Ltd. All rights reserved. c) Structure of the alpha‐keratin and the beta‐keratin filaments.^[^ ^212^ ^]^ Copyright 2015 Elsevier Ltd. All rights reserved. d) Molecular structure of fibrillar collagens.^[^ ^215^ ^]^ Copyright 2003 Elsevier B.V. All rights reserved. e) Top view of SEM observations on GP‐CMTs.^[^ ^221^ ^]^ Copyright 2018, American Chemical Society. f) Dark‐field high‐resolution TEM image, and corresponding EDX elemental mapping showing the distribution of C, N, and Fe.^[^ ^223^ ^]^ Copyright 2019 Wiley‐VCH Verlag GmbH & Co. KGaA, Weinheim. g) Schematic diagram for the synthesis of the Pd/ HNC.^[^ ^235^ ^]^ Copyright 2019 Hydrogen Energy Publications LLC. Published by Elsevier Ltd. All rights reserved.

Chitin is the second largest biomass polymer after cellulose^[^ ^194^ ^]^ and is also an ideal biomass precursor because of its higher nitrogen concentration, thermal stability, and carbon yield than cellulose. Similar in structure to cellulose, chitin is a linear polysaccharide polymer consisting of thousands of acetylglucosamine linked into a polymer by b‐1,4 glycosidic chains.^[^ ^195^ ^]^ Pyrolysis of chitin, when the temperature is at 250–280 °C, the deacetylation and deacetylation products on the C‐2 position of chitin are continuously decomposed, and the product chitosan is finally obtained.^[^ ^196^, ^197^, ^198^ ^]^ As shown in **Figure** **7**a, the chitosan molecular chain has a large number of ─OH groups and ─NH_2_ groups. This means that it can be coordinated with transition metal or rare earth metal ions in dilute solutions to form chitosan metal complexes, and provides conditions for the preparation of modified animal‐based CAC.^[^ ^199^, ^200^, ^201^ ^]^ After chemical deproteinization, chemical demineralization, and mechanical grinding methods chitin can be extracted and obtained, while the final yield all depends on the type of biomass precursors (mollusks, insects, and crustaceans, etc.^[^ ^202^ ^]^) and the treatment method chosen.

The chitin content of crustacean shells of crustaceans and the pupal shells of insects in nature is high, up to 80%. Shrimp shells and crab shells are the most common biomass wastes produced by crustaceans in daily life, and the CAC materials, after activation, carbonization, and acid treatment, all exhibit channel‐like microporous/mesoporous structures. In addition to removing impurities, the acid treatment creates channel‐like mesopores or macropores. This pore structure shortens the ion diffusion path and facilitates ion penetration.^[^ ^203^, ^204^, ^205^ ^]^ In addition to changing the pore structure by creating pores during the reaction, the active agent can introduce heteroatoms that increase the number of active sites on the one hand and cause a large number of defects on the other. Reactive activity can also be enhanced. For example, the presence of nitrogen atoms can improve electron conductivity and surface adsorption capacity by providing one or two electrons to the π‐bond.^[^ ^206^ ^]^ However, not all catalyst designs revolve around modulating the catalyst as much as possible toward a channel‐like multilayer pore structure with medium or large pores. Compact and dense pore structures enable fast electron and ion transport kinetics. Microporous carbon with a large surface area and high bulk density meets the need for high bulk capacitance for electrode material applications. The highly cross‐linked structure formed by the Schiff base reaction during the pyrolysis of chitosan is utilized to inhibit foaming while promoting the formation of a densified backbone. Ultimately, the chitosan‐based microporous carbon designed using thermally induced Schiff base reaction obtained a large surface area (2007 m^2^ g^−1^), and high compaction density (1.21 g cm^−3^). High capacitances of 280 F g^−1^/339 F cm^−3^ at 1 A g^−1^ were achieved in practical applications.^[^ ^207^ ^]^

Controlling the pyrolysis temperature can regulate the growth of impurity materials and regularize the pore shape, and this also makes the final carbon‐based pores show a uniform multilayer dense distribution. Also, controlling the pyrolysis temperature can change the structure of carbon from *sp* ^3^ hybridization to *sp* ^2^ hybridization, and this hybridized carbon configuration is also essential for inducing the electron transfer path. Elevated *sp* ^2^ hybridized carbon content can make the final prepared CAC inclined to the 4‐electron transfer path in the ORR.^[^ ^208^, ^209^ ^]^ This provides a perfect focus for the purposeful design of CAC‐based catalysts in the future, i.e., focusing on the design and regulation of porosity and carbon configuration. Insect cuticles have less inorganic material than the shells of crustaceans, and the extraction of chitin obtained from cicada molts, for example, is also much higher than that of crab shells. The obtained CAC derived from cicada molts retains the characteristics of well‐developed micropores, large surface area, and abundant heteroatoms as seen in Figure 7b, resulting in improved capacitive behavior and exhibiting high capacitance and high power density in electrode applications.^[^ ^210^ ^]^ In addition to using biomass materials containing chitin as CAC precursors for direct use, biomass materials can also be converted into chitosan combined with CAC to synthesize bio‐nanocomposites by acoustic‐chemical methods. This method makes good use of the adsorption capacity of amine and hydroxyl functional groups in chitosan, and the combination with the stable CAC can avoid the disadvantages of low mechanical and chemical stability of chitosan. Combining the advantages of CAC and chitin by‐products (chitosan) is innovative, but the method is designed from the physical–mechanical combination point of view, and direct preparation from biomass materials should be considered to obtain The CAC material for in situ growth of chitosan should be considered as a way to streamline the experimental steps and reduce the production cost.

Changing the amino acid sequence and steric structure results in proteins with different properties, such as keratins, silk proteins, and collagens. Keratin contains alpha‐keratin and beta‐keratin, in alpha‐keratin the polypeptide chains are arranged in alpha‐helices, while in beta‐keratin they are arranged as folded beta‐sheets as shown in Figure 7c. The superhelical sequence of keratin contains three types of intermolecular bonds, covalent disulfide cysteine crosslinking, ionic salt bonding, and non‐covalent hydrogen bonding, that also result in good thermal and chemical stability of keratin.^[^ ^211^, ^212^ ^]^ Keratin is usually obtained in hooves, claws, and horns after carbonization to obtain biochar that maintains high yield and high performance. Silk proteins mainly consist of discrete beta‐sheet microcrystals and amorphous structural domains. After heat treatment, β‐sheet microcrystals can be transformed into *sp* ^2^ hybridized carbon structures,^[^ ^213^, ^214^ ^]^ containing many volatile gases of ─OH, ─C═O,─C─N, and ─NH_2_ groups spilling out at temperatures below 400 °C. The graphitization of the CAC material obtained increases with increasing temperature, and eventually, biomass with excellent electrical conductivity is obtained from CAC with excellent electrical conductivity. Collagen, on the other hand, is mainly distributed in bones, tendons, ligaments, and other tissues, forming a fibrous network structure in the form of collagen protofibrils and collagen fibers. The basic structure of collagen is a right‐handed three‐stranded helical structure formed by three alpha‐peptide chains, with glutamic acid uniformly distributed in the center of the helix and non‐helical C‐ and N‐terminal peptides distributed at both ends connected to other proteins or extracellular matrix by covalent bonds to form collagen protofibrils or extracellular matrix cross‐linked network as shown in Figure 7d.^[^ ^215^, ^216^ ^]^ The glycine, alanine, and proline on the collagen polypeptide chain contain a large number of C, N, O, and S atoms. Therefore, the porous carbon obtained by pyrolysis treatment is rich in heteroatoms. In particular, the preparation of CAC using animal bones as precursors retains the structural advantages of heteroatom doping and high porosity while can be applied as natural nanostructure templates for electrochemical energy storage/conversion devices.^[^ ^217^, ^218^ ^]^

The biomass‐derived CAC obtained by pyrolytic carbonization using avian animal feathers as precursors shows a multilayer microporous structure, in contrast to the honeycomb‐like mesh structure of CAC materials of plant origin. After setting the temperature and pressure of pyrolysis, the keratin barrier is weakened and the abundant functional groups such as carboxyl, hydroxyl, and amino groups on the surface of the characteristic primitive beta‐folded structure therein are exposed. The abundant functional groups coupled with the unique hierarchical porous structure play an important role in applications for adsorption, photo recycling, and bioelectrochemistry.^[^ ^33^, ^219^, ^220^ ^]^ Animal hair is also a low‐cost and environmentally friendly precursor material. The presence of cysteine in hair makes the hair‐forming carbon‐rich in multiple heteroatoms and improves the electrochemical energy storage performance of the electrode through the Faraday Effect and synergistic effect. Moreover, the uniform and dense nickel nitrate hydroxide nanofilms grown around the hair surface can be graphitized rapidly at moderate temperatures and form rich pore channels as seen in Figure 7e. Such open/porous channels facilitate the smooth entry of ions deep inside the carbon matrix and expose more reaction sites. The internal carbon matrix activators are activated gradually as the reaction proceeds until the whole electrode is utilized, and the deep functionalized heteroatoms participate into The participation of deeply functionalized heteroatoms in the reaction greatly improves the specific capacity and the utilization efficiency of the active material, ultimately presenting excellent output capacity and gradually increasing stability, extending the cycle life.^[^ ^221^ ^]^ Moreover, this structure applied to Li‐S batteries can fill the raw sublimated sulfur into the carbon matrix by capillary force, provide enough space to accommodate sulfur and anchor soluble polysulfides, inhibit the dissolution of long‐chain polysulfides during charging and discharging, and buffer the volume expansion. Buffering volume expansion, while the doped heteroatoms enhance the affinity between polysulfide and carbon skeleton, effectively suppressing the shuttle effect, thus enhancing electrochemical performance and electrical stability.^[^ ^222^ ^]^

When we think of silk protein‐derived CAC, we usually think of silk, a commonly used protein precursor material. The thermal annealing treatment of natural silk proteins dissolved in transition metal salt solution still retains the beta‐sheet structure of silk fibrous proteins. The abundant amino functional groups in its chemical structure strongly interact with transition metal ions anchored in the silk protein precursor framework, ensuring a high dispersion of atomic active sites as seen in Figure 7f.^[^ ^223^ ^]^ Meanwhile, the CAC materials obtained after carbonization of the extracted nitrogen‐rich filament proteins are variable in form and can be prepared as one‐dimensional fibers, 2D carbon sheets, and three‐dimensional carbon nanotubes by simple carbonization. To obtain the final porous structure, it is necessary to modulate the concentration of transition metal salt solution and activator to act as a pore‐forming agent during pyrolysis, while the final obtained *sp* ^2^ hybrid carbon network and highly defective structure provide a high specific surface area. Using the lamellar structure of the filamentous protein pyrolyzed into multiple carbon layers can tightly encapsulate the metal nanoparticles and adjust the central metal atom local structure by changing the quantitative coordination bonds between N and metal atoms through pyrolysis to synthesize metal single‐center catalysts effectively and feasibly.^[^ ^223^, ^224^ ^]^ While in the preparation of carbon nanofibers using the electrostatic spinning method, the concentration of the activator affects whether the β‐sheet structure is transformed into an amorphous structure. Too low concentration will confine the silk protein molecules in the β‐sheet crystal structure, exacerbate the fragmentation movement causing fiber expansion, and ultimately lead to fiber melting to form lumps. Therefore the regulation of activator concentration is crucial.^[^ ^225^ ^]^ In addition to silk proteins from cocoons, silk gum has good biocompatibility and is rich in small molecules of hydrophilic amino acid residues that can interact with carbon‐based aromatic groups. The addition of silk gum in the preparation of CAC‐based catalysts can stabilize the surface dispersion of the carbon base and change the surface properties, providing an involved idea for the development of conductive inks for flexible wearable electronic devices.^[^ ^226^ ^]^

As mentioned earlier, collagen‐derived carbon, represented by animal bone, is rich in in situ dopant atoms and maintains a stable composition that is not susceptible to significant changes due to the growth environment. Moreover, animal bone contains hydroxyapatite and calcium phosphate that is not only precursors for phosphorus doping but also can induce self‐activation in situ.^[^ ^227^ ^]^ Under the combined effect of natural pores and organic matter pyrolysis, the final obtained CAC has a graded porous structure. This structure reduces resistance, accelerates electron transfer, and expands the specific surface area to expose more active sites. The catalyst conductivity and reaction rate are improved by ensuring efficient mass transfer of reactants through the pores. In conclusion, different types of pore structures in the hierarchical porous structure of the CAC matrix were obtained to play different roles. Among them, macropores shorten the diffusion distance of electrons, mesopores are a low resistance channel, and micropores enhance the adsorption capacity.^[^ ^228^, ^229^ ^]^ As known, graphene‐N and pyridine‐N are *sp* ^2^ hybridized and have similar bond lengths for C─C and C═N instead. Thus C─N is bonded to an O atom and two C atoms at the edge of graphene, and this provides a π system with p electrons. C─C replaces the C atom in the hexagonal ring and can improve the conductivity because it affects electron transport during charge/discharge. The π‐electron delocalization of pyrrole‐N and strong adsorption of *OH is suppressed by pyrrole‐N. Pyrrole‐N is *sp* ^3^‐hybridized, contributing two p‐electrons to the π system, disrupting the planar structure of graphene, and the lone pair of electrons occupies the vertical pz orbital, contributing to the aromatic six‐membered structure. Therefore, different pathways of the catalytic reaction are targeted to modulate the specific gravity of graphene‐N, pyridine‐N, and pyrrole‐N. Upon controlled pyrolysis temperature increase, the nitrogen atoms in the hexagonal graphite structure provide additional electrons to the conjugated system, reducing support scattering thereby enhancing support concentration and increasing conductivity, but note that too high pyrolysis temperature can remove the heteroatom N by volatilization of NH_4_ single bond N fraction, NO_3_ single bond N fraction and N‐containing groups, resulting in a reduction of the active site.^[^ ^230^ ^]^

### 3.3 Microbial‐Based Biomass and its Chemical Composition

Microorganisms have a very fast reproduction rate and can grow exponentially in a short period, making them more resourceful and easier to obtain than other types of biomass. At the same time, due to their small size, microorganisms have a simpler structure and pure components than plants and animals. The chemical composition of microorganisms includes carbohydrates (chitin), proteins, fibers, fats, and ashes. The use of widely distributed fungi carbonized at high temperatures to retain a porous framework with high surface area, high bulk density, and graded interconnections is an ideal alternative carbon source.^[^ ^231^ ^]^ Exceptionally, bacterial cellulose is a typical biomass feedstock with a higher degree of polymerization, higher crystallinity, and 100% cellulose content than plant cellulose. The CAC‐based catalysts obtained by high‐temperature carbonization of bacterial cellulose exhibit rich 3D pore structure, high specific surface area, and electrical conductivity, and are candidate carbon‐based materials for high‐performance electrocatalysis.^[^ ^232^, ^233^ ^]^

Interestingly, some bacteria have a highly adaptive metabolism and are well tolerant to metal ions. The presence of many oxygen‐containing functional groups in the bacteria can adsorb reduced metals under specific conditions when they are exposed to high concentrations of metal ions as seen in Figure 7g.^[^ ^234^, ^235^, ^236^ ^]^ Based on this property, metal doping of catalysts was achieved by high‐temperature carbonization of bacteria treated with adsorbed metals to increase conductivity, and metal loading was increased by exposing the internal oxygen functional groups through cell fragmentation.^[^ ^237^ ^]^ To solve the aggregation of metal ions, strains with good wettability to metal solutions were selected to minimize the agglomeration and precipitation during the uptake of metal ions, thus improving the electrocatalytic catalytic activity of the corresponding catalysts.^[^ ^238^ ^]^

### 3.4 Summary of Catalytically Active Carbon Synthetic Sources

Whatever the biomass, it can be subdivided into organic molecular polymers rich in functional groups. The exposure of oxygen‐containing groups after pyrolysis of biomass materials at high temperatures and the formation of pores after the escape of pyrolysis gases contribute to the catalytic reaction of the catalyst. Nevertheless, the pursuit of complex molecular weight and functional groups will compromise the mechanical properties and electrical conductivity of the catalyst. The dynamics of biomass chemical components with pyrolysis temperature is the primary factor to be investigated, while the degree of graphitization after high‐temperature carbonization of biomass CAC materials should be taken into account, and the optimum value of carbonization temperature is determined by balancing the two.

The original form of biomass present influences the form and pore structure of the carbon matrix after pyrolysis, for example, plant branches and roots‐derived CACs mostly present a honeycomb mesh structure, while feather‐ and hair‐derived CACs present a multilayer microporous structure. The choice of suitable precursors for the preparation of catalysts for different application scenarios is a good choice to take advantage of the structural characteristics of the precursors themselves to obtain the desired structure and controlled morphology. Alternatively, from the production cost point of view, a low‐cost biomass precursor can be adapted/designed with the suitable process to change the original structure and further modified to obtain a highly active CAC‐based catalyst suitable for different application scenarios. Therefore, the right precursor with the right process can be considered as a complete catalyst design idea, but the whole idea of preparation should be based on a “customized” model with functional/performance requirements as the starting point. When a biomass‐derived CAC or composite material has reached the peak of its performance in a particular field of application, optimization/refinement of the design should be considered to enable it to serve different application scenarios at a high level and to give the CAC‐based catalyst a universal character.

## 4 Synthesis Methods of Biomass‐Derived Catalytically Active Carbon

The conversion of biomass into CAC materials undergoes many reactions, and the whole process is accompanied by the transfer of heat and chemicals. The choice of carbonization method plays a decisive role in determining the final biomass‐derived CAC type, while the choice of carbonization method and various parameters such as time, temperature, reagents, surface properties, and availability together influence the physicochemical properties such as morphology, specific surface area, porosity, chemical composition, functional groups, and degree of graphitization.^[^ ^239^ ^]^ Pyrolysis and hydrothermal carbonization are commonly used to extract carbon from biomass. The former is a process of heating carbon‐rich feedstock in a limited oxygen or inert atmospheric environment at a specific temperature level (300–600 °C),^[^ ^240^, ^241^ ^]^ whereas hydrothermal carbonization is a thermochemical process used to convert biomass to carbon and relies on water as a medium and a pressurized environment of 120–250 °C. It uses subcritical water to convert biomass to carbon products, thereby effectively dehydrating and hydrolyzing oxygen‐rich functional groups of hydrogenated coke precursors. Other functional groups, such as nitrogen‐containing groups, can also be applied in the use of additives or doping of hydrogenated coke‐containing precursors, thus leaving a high oxygen and nitrogen content in the biomass residue, while the surface of the carbonized material is rich in oxygen‐ and nitrogen‐containing functional groups and has multiple uses.^[^ ^242^, ^243^ ^]^ In contrast, hydrothermal carbonization reacts faster and requires shorter reaction time, so the former consumes more energy than the latter. The final specific surface area of CAC materials obtained by direct pyrolytic carbonization is not satisfactory although the porosity increases with increasing temperature, and the large loss of active sites in the process reduces the reaction activity.^[^ ^244^ ^]^ Complex components in biomass materials are somewhat uncontrollable by thermal decomposition into low molecular weight materials by carbonization alone. Preparation of CAC materials with adjustable porosity becomes a luxury. At this point, effective strategies to regulate the conductivity, electron transport rate, capacity, and crystal structure of catalysts can be explored using physical and chemical methods or a combination of physical and chemical methods. Eventually, biomass can be efficiently converted into high‐value‐added carbon products.^[^ ^245^, ^246^ ^]^

Activation is an excellent option for the efficient conversion of biomass precursors. The activation process opens previously inaccessible pores, forms new pores, and enlarges existing pores. The end result is a general increase in porosity and surface area, the formation of ordered and tunable structures, and the conversion of CAC materials into highly porous CACs.^[^ ^247^, ^248^ ^]^ In addition to applying activation treatments to optimize the structure of CAC materials and improve their catalytic performance, there are other treatments that can modulate the macroscopic/peripheral structure of CAC materials, such as using pretreatment,^[^ ^249^, ^250^ ^]^ freeze‐drying technique,^[^ ^251^, ^252^ ^]^ template method,^[^ ^253^, ^254^ ^]^ etc. Ultimately, they exhibit better performance and provide more possibilities for the wide application of CAC materials.

### 4.1 Carbonization

#### 4.1.1 Pyrolysis Carbonization

Pyrolytic carbonization is one of the most commonly used carbonization methods. Pyrolytic carbonization is usually carried out by introducing nitrogen, argon, and other protective gases using instruments such as tube furnaces or muffle furnaces. In addition to the basic parameters such as temperature, heating rate, holding time, and gas to control the structure and properties of the final CAC material, the whole preparation process will be optimized by other experimental methods, reaction media, and even from the material structure itself to achieve the best results.

**Figure 8:**
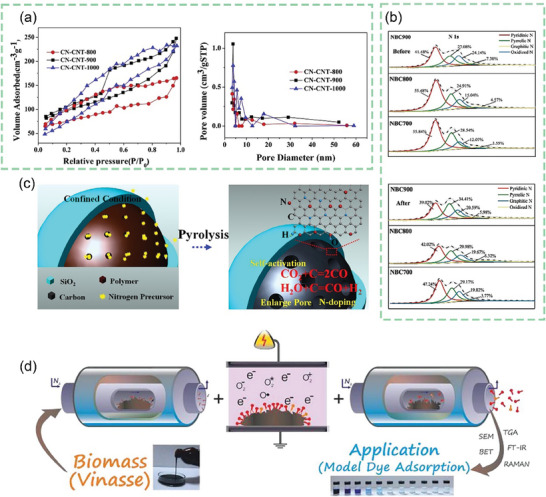
a) Nitrogen adsorption–desorption isotherms and the pore size distribution of GN‐CNT‐800, GN‐CNT‐900, GN‐CNT‐1000.^[^ ^256^ ^]^ Copyright 2017 Elsevier B.V. All rights reserved. b) N 1s of NBC700, NBC800 and NBC900 before and after reaction.^[^ ^260^ ^]^ Copyright 2021 Elsevier B.V. All rights reserved. c) Schematic illustration for activation and N‐doping of N‐MCS inside compact silica shell.^[^ ^272^ ^]^ Copyright 2019, Elsevier. d) preparation of CAC by cold oxygen plasma treatment.^[^ ^269^ ^]^ Copyright 2017 Elsevier B.V. All rights reserved.

Along with the whole heating process, the biomass precursors first evaporate water and volatiles and react to form hydrogen peroxide, ─COOH, and ─CO groups. As the temperature rises to 200 °C, macromolecules in biomass decompose. For example, hemicellulose and cellulose in plants will first rapidly volatilize and decompose, followed by the degradation of lignin and other organic materials with stronger chemical bonds and higher thermal stability. And animal‐based biomass materials such as chitin, proteins, and other macromolecules will also denature/decompose into smaller molecules and eventually carbonize.^[^ ^255^ ^]^ As shown in **Figure** **8**a, the decomposition of these organic macromolecules removes or cleaves pore‐clogging substances, equivalent to enlarging the available surface area. And the gases inevitably produced during the reaction accelerate the generation of micropores. All these reaction changes favor the increase of surface area and pore volume as well as the construction of pore structure.^[^ ^256^ ^]^ At the same time, the variety and concentration of functional groups on the surface of carbon matrices have increased. These functional groups play an important role in applications such as organic/inorganic pollutant adsorption on CAC materials. The carbon structure of biomass‐derived CAC becomes dense and the carbon content increases upon increasing pyrolysis temperature. However, the ash content increases with increasing pyrolysis temperature due to the gradual concentration of inorganic and organic residues. Therefore, the ash content can be reduced by subsequent chemical acid treatment.^[^ ^257^ ^]^ The structural changes of biomass‐derived CAC directly depend on the pyrolysis temperature. During the pyrolysis process the carbon matrix increases the specific surface area in addition to forming some cracks and pores. The increase in pyrolysis temperature increases the degree of graphitization. When the temperature reaches 700 °C, the biomass‐derived CAC shows a graphitized carbon skeleton. The graphitized carbon skeleton provides a site for water retention and adsorption of chemical elements. Nevertheless, it should be noted that too high pyrolysis temperatures also accelerate the degree of carbonization and can destroy or enlarge the microporous structure. At temperatures of 850–900 °C, external ablation of the carbon particles severely damages the high porosity. Due to the substantial widening of the micropores, the microcrystals on the pore wall react with the active agent, which may even lead to the collapse of the pore wall.^[^ ^258^, ^259^ ^]^ A point worth noting is that biomass‐prepared CACs are readily doped with nitrogen, as shown in Figure 8b. The form of nitrogen is also easily affected by the pyrolysis temperature. It is easy to transform from unstable pyridine nitrogen and pyrrole nitrogen to stable graphitic nitrogen. The lone pair of electrons on the pyridine nitrogen can change the charge distribution by conjugating with the free‐flowing π‐electrons on the biomass‐derived CAC. However, when nitrogen‐doped CACs prepared by pyrolysis at 900 °C are applied for PMS activation, the nitrogen transition leads to electron transfer from neighboring atoms. The graphitic nitrogen becomes the active site.^[^ ^260^ ^]^

Therefore, the effect on the morphological transformation of the doped atoms and the construction of active sites should be considered when setting the experimental parameters according to the catalytic reaction. Controlling the heating rate will result in either rapid cracking of the controlled biomass material to produce carbon‐less products of gas and tar (high‐speed pyrolysis) or conversion to carbon‐rich products (slow pyrolysis). When the heating rate is faster, the pyrolysis reaction will also be completed in a shorter time. At the same time the contact time between the pyrolysis vapor and the biomass particles will be shortened. During the whole reaction process, the time for the secondary carbonization reaction to occur may be shorter, or the secondary carbonization reaction that occurs may be insufficient. The specific gravity of the carbon‐containing precursors, light bio‐oils, and volatiles of the final prepared catalysts will subsequently increase. In the case of CAC materials prepared by the CaO template method with a set heating rate of 2 K min^−1^, the pyrolysis vapor will further carbonize with the calcium citrate in the carbon precursor to generate CO_2_ and H_2_O for internal activation to develop the pore structure.^[^ ^261^, ^262^ ^]^ Nevertheless, the use of a strongly acidic catalyst can increase the carbon yield. In the case of fast lignin pyrolysis, the whole pyrolysis process causes selective production of H_2_S, the strongly acidic part of catalytic cracking. Oxygen‐containing compounds are produced when H_2_S, a non‐condensable gas, is catalytically cracked. The low molecular compounds formed by the dehydration reaction of this substance cause blockage when they enter the micropores of the carbon matrix. Unpolymerized dehydration intermediates readily form coke deposits thereby increasing carbon yield. However, the size of the oxygenated compounds produced by rapid pyrolysis of lignin needs to match the size of the CAC pore. Structural constraints in the carbon skeleton pore structure will ultimately promote coke formation from the dehydrated intermediates.^[^ ^263^ ^]^

In general, the inert gas N_2_/Ar is passed to the CAC preparation by pyrolysis using a tube furnace or muffle furnace to act as a protection against reaction with precursors during pyrolysis and to improve the controllability of the product. Nonetheless, from another perspective, specific gases can be introduced to participate in the pyrolysis reaction in order to dope the atoms or increase the reactivity. Commonly, physical activation with CO_2_ or H_2_O is used to increase the reaction activity. Other less common physical activation methods, such as the use of NH_3_ atmospheres capable of inducing stronger π‐electron delocalization to prepare nitrogen‐doped carbon supports with stronger electron supply capabilities. Nitrogen doping increases the intrinsic reactivity of the catalyst.^[^ ^264^, ^265^, ^266^ ^]^ In addition, the use of NH_3_ as a nitrogen source for nitrogen doping results in the removal of additional C atoms from the M‐N_4_ site, allowing partial conversion of the pyridine nitrogen configuration to pyrrole nitrogen.^[^ ^267^ ^]^ Even more novel was the adherence to a pre‐oxidation and ammonolysis design using an O_2_/NH_3_ mixture. However, the final carbon material was not optimal for separating coal coke in space. The cellulose carbonized into twisted, ultrathin graphite flakes that did not retain the dense fiber bundle structure. It is not unique that hydrogen sulfate (H_2_S) can be utilized to achieve the effect of S‐doped CAC materials. The combination of sulfur doping with microporous carbon tubes was applied to sodium‐ion batteries (SIBs). Therein, sulfur reacts with sodium as a high‐capacity electroactive element, while the porous structure of the carbon matrix improves the electrical conductivity. Excellent rate performance, high charging capacity, and excellent cycling performance are finally obtained.^[^ ^268^ ^]^ This shows the possibility of providing more structure and activity through the choice of atmosphere setting as a means of regulating the structural performance of the catalyst.

In addition to adjusting the experimental parameters to change the structural properties of CAC materials, pyrolysis will be combined with other experimental tools or techniques (Figure 8c,d). The reactive oxygen atoms in cold oxygen plasma can easily form oxygen functional groups with the carbon surface. The use of cold oxygen plasma increases the reactivity, the number of oxygen‐containing functional groups, and the hydrophilicity of CAC. Therefore, the generation of oxygen radicals, oxygenated ions, and molecules after treatment of biomass precursors with cold oxygen plasma can improve chemical activity. C═O and C─O─C groups are partially or completely reduced to C═C bonds during the reaction due to plasma etching. The disruption of the *sp* ^2^ hybridized graphite structure by this change produces defects in the carbon matrix. Eventually, the defects are transformed into ordered graphitic structures as active sites. The graphitized ordered structure combines with oxygen‐containing functional groups to functionalize the CAC material. As shown in Figure 8d, the specific surface area can be effectively increased by combining the cold oxygen plasma after setting a suitable pyrolysis temperature. However, setting too high a pyrolysis temperature can lead to the clogging of pores with oxygen‐containing functional groups or even the collapse of the pore structure. So the first pyrolysis temperature determines the availability of cold oxygen plasma in the oxidation stage. Compared to conventional preparation methods, cold oxygen plasma technology shortens the test cycle and is an eco‐friendly oxidation method.^[^ ^269^ ^]^ The residence time of organic vapors (hydrocarbons) in the pyrolysis process is shorter than under atmospheric pressure conditions when the vacuum pyrolysis method is employed by abandoning the N_2_/Ar gas cycle. Less carbonaceous deposits are also produced on the surface and in the pores of the final CAC obtained. Therefore, CAC‐based catalysts prepared by vacuum pyrolysis are more likely to produce mesoporous and mesoporous carbon structures. The specific surface area and porosity of vacuum pyrolyzed carbon materials increased accordingly. Since vacuum pyrolysis carbon has a wider spatial pore structure, this pore structure can be utilized to adsorb macromolecules.^[^ ^259^, ^270^, ^271^ ^]^ The desired structure and properties can be achieved by modulating the carbon structure to achieve self‐encapsulated pyrolysis without changing the experimental means. Ordered mesoporous polymer spheres (MPS) treated with uniform dense silica layer coating were used as carbon precursors. The 2‐methylimidazole (Hmim) is adsorbed into the MPS and used as a nitrogen precursor for restricted pyrolysis. The in‐situ gases (CO_2_, H_2_O, etc.) generated from the decomposition of Hmim and MPS will act as activators to activate the carbon walls of the MCS while forming a macroporous structure by etching the adjacent pore walls. The decomposition of Hmim also leads to in situ N doping of the carbon skeleton. As shown in Figure 8c, it is this self‐activation effect and N doping behavior that somewhat disrupts the orderliness of the carbon structure and increases the surface area, pore volume, and mesopore size. The reconfiguration of the carbon structure effectively increases the capacitance and improves the electrochemical properties.^[^ ^272^ ^]^ Dense silica shells play a key role in confined pyrolysis methods. The presence of the silica shell provides a nanoreactor for the polymer. The boundary established by this reactor prevents the polymer and carbon spheres from sticking together due to high temperatures. The silica shell also allows the volatile carbonaceous material to be efficiently converted to solid carbon during the reaction process. Silica shells have the defect of being fragile and not resistant to high pressure. In order to prevent the rupture of silica shells, formation of carbon fragments by pyrolysis gas escape, and reduction of internal residual carbon, the content of ethylene orthosilicate (TEOS) can be varied. Different thicknesses of silica shells can be prepared by fine‐tuning the structural parameters to achieve different hollow structures.^[^ ^273^ ^]^

#### 4.1.2 Hydrothermal Carbonization

Hydrothermal carbonization (HTC) mimics the natural process of a coal formation by converting carbohydrates, organic molecules, or biomass into carbon in a subcritical water state and at relatively low temperatures (120–250 °C).^[^ ^242^, ^274^, ^275^ ^]^ Thus, the HTC process produces a partially carbonized product with a high density of oxygen‐containing groups and low condensation. Hydrothermal carbon has great advantages in electrochemical and other applications. The HTC dehydration process releases one‐third of the combustion energy and obtains higher yields (≈70–80%) compared to pyrolysis or chemical activation processes, even with carbon efficiencies close to 1 under the right conditions and for the right reaction time. In the whole HTC process, carbonization of biomass materials is reached after biomass dehydration, fragment polymerization, and intermolecular dehydration.^[^ ^44^, ^276^ ^]^ Similar to pyrolysis, the main controllable parameters of HTC can be summarized as biomass precursors, temperature, residence time, and doping.^[^ ^277^ ^]^ Thus, the physical and chemical interactions between reagents and solvents facilitate the final formation of the desired CAC material from the treated biomass after modulating the hydrothermal conditions related to the reaction rate and time.^[^ ^278^ ^]^ It also means that the structure, composition, and morphology of the biomass‐derived CAC can be precisely regulated by changing the reaction conditions.^[^ ^243^, ^279^, ^280^ ^]^

**Figure 9:**
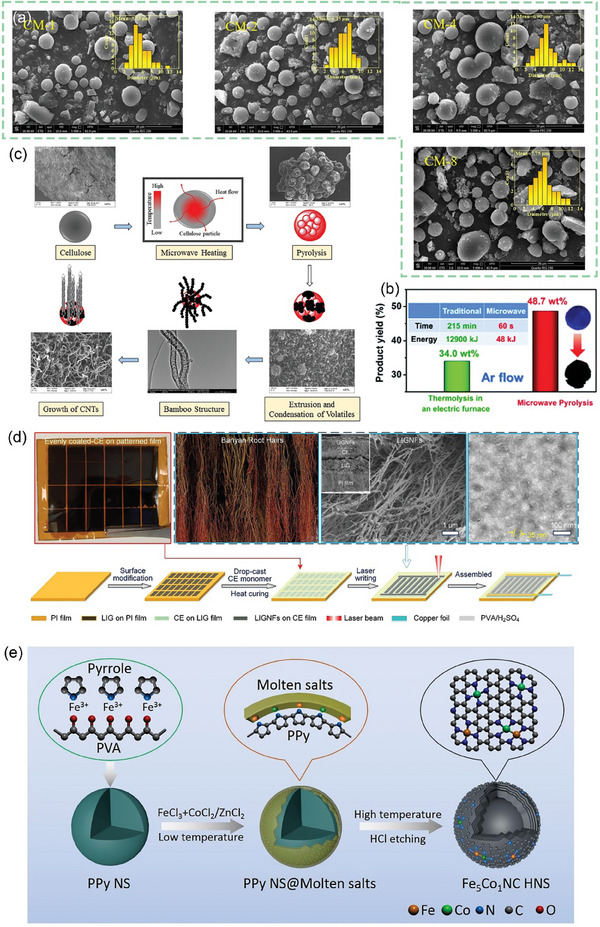
a) Microscopic morphology of the coke microparticles obtained at different HTC residence times.^[^ ^281^ ^]^ Copyright 2022 Elsevier Ltd. All rights reserved. b) comparison of product yield (*m*(product)/*m*(Co‐MOF precursor)), total consuming time, and energy consumption (estimated only by equipment power multiplied by operation time) for Co‐MOF‐800 °C^−1^ h and Co‐MOF‐microwave‐60 s.^[^ ^43^ ^]^ Copyright 2024 Copyright Clearance Center, Inc. All rights reserved. c) Schematic illustration of the mechanism of formation and growth of CNTs during microwave‐induced pyrolysis of cellulose.^[^ ^287^ ^]^ Copyright 2019 Elsevier Ltd. All rights reserved. d) Schematic diagrams and corresponding photographs/images for the preparation of LIGNFs‐IMSCs by laser modification and direct laser writing approach.^[^ ^296^ ^]^ Copyright 2020 Elsevier B.V. All rights reserved. e) Schematic illustration of the synthesis of PPy nanospheres (NS) and surface anchorage of Fe and Co atoms to nitrogen‐doped carbon hollow nanospheres (HNS) with the assistance of ZnCl_2_.^[^ ^308^ ^]^ Copyright 2020 Elsevier B.V. All rights reserved.

Depending on the operating temperature, HTC can be divided into two categories: one is high‐temperature HTC, a method with a pyrolysis temperature of 300–800 °C, mainly used to produce highly graphitized CAC materials, such as carbon nanotubes or CAC; the other is low‐temperature HTC with a pyrolysis temperature below 300 °C. The low‐temperature HTC process is milder and therefore suitable for the preparation of various functional CAC materials for electrolysis. It is suitable for the preparation of various functional CAC materials for electrolysis.^[^ ^45^ ^]^ Water is both solvent and reaction medium, so there is no strict requirement to keep the material dry. More streamlined experimental steps avoid the cost of adding to the production process and are more economical. Moreover, some of the gases produced in the carbonization process can be dissolved to reduce air pollution. The HTC process thermally compresses water changing the viscosity of the water by increasing the temperature. Water easily penetrates into the porous medium under high temperature and pressure conditions. When the temperature exceeds the activation energy, the polymer components in the biomass initiate degradation and depolymerization due to hydrolysis. Eventually, the ether and ester bonds of the biomass material are broken and the ash content is reduced. A controlled residence time determines the degree of polymerization of the soluble intermediate fragments. Short residence times result in cracks and grooves on the carbon surface. However, increasing the residence time to more than 6 h results in the formation of carbon microspheres and even aggregation of carbon microspheres on the carbon surface, as shown in **Figure** **9**a.^[^ ^281^ ^]^ After the fragment compounds are stabilized in the hydrothermal process, reducing the heating rate allows the fragments to undergo secondary reactions to form solid carbon. The disadvantage of hydrothermal carbon, however, is that it usually lacks intrinsic porosity.

The degradation of macromolecules during the hydrothermal process is highly controllable to regulate the morphology of CAC materials with small molecules, but hydrothermal carbon usually lacks inherent porosity and does not have a tunable pore structure. Therefore, post‐thermal carbonization (>350 °C) or templating is required to prepare porous carbon structures.^[^ ^282^ ^]^ The pore structure of the controlled CAC material is synthesized by hydrothermal liquefaction of the biomass precursor into a liquid, using a template to polymerize with the liquid into a biomass polymer.^[^ ^47^ ^]^

#### 4.1.3 Microwave‐Assisted Carbonization

Conventional heating methods mainly transfer heat from the outside of the material to the inside of the material through heat conduction, heat convection, and heat radiation, so that the temperature of the outside of the material is higher than that of the inside. Under microwave heating, the material as a whole absorbs microwave energy, and the interior of the material continuously and strongly dissipates heat to the outside due to the high temperature. The entire process is independent of the rate of heat conduction and convection. Therefore, microwave heating can control the start and pause of heating and has a faster heating rate. Higher microwave power provides enough energy to break the chemical bond between carbon and oxygen, converting the oxygen‐containing compounds in carbon into less volatile compounds. Simultaneously breaking the carbon‐carbon single bond promotes the rearrangement of carbon atoms. Concentration and retention of elemental carbon is achieved by binding the carbon–carbon double bonds.^[^ ^283^, ^284^ ^]^ Microwave pyrolysis of MOF wrapped with graphene powder can prepare high‐performance MOF‐derived electrocatalyst in the 1960s, compared with conventional rapid pyrolysis even if the controlled pyrolysis temperature. In contrast, conventional fast pyrolysis cannot reach the energy consumption level of microwave pyrolysis of only 0.37% even if the controlled pyrolysis temperature reaches 20 °C S^−1^ as seen in Figure 9b. Nanosheets before and after microwave pyrolysis show well‐preserved structures. The distribution of these highly graphitized carbon nanotubes improved the mass transfer capacity and increased the active sites. In contrast, conventional pyrolysis for too long a time can lead to the collapse of the nanosheet structure due to excessive decomposition of organic ligands.^[^ ^43^ ^]^

It is important to note that due to the inhomogeneous nature of the material, microwave heating can lead to uneven temperature distribution resulting in some areas reaching higher temperatures faster than adjacent areas. A large temperature gradient exists in the material as a whole. From the point of view of the heating principle and characteristics, microwave pyrolysis cannot precisely adjust the heating rate and pyrolysis temperature to control the structure and properties of CAC materials. Moreover, the microwave penetration depth of conventional microwave‐absorbing materials is 1–2 cm. The stronger the absorption properties of the material, the lower the penetration depth. Therefore, the size of CAC materials is also limited.

The biomass material was used as a precursor for the preparation of CAC by microwave pyrolysis. Due to the good water absorption inside the biomass, it was released quickly by heating. The pore structure and specific surface area of the CAC materials from microwave pyrolysis were increased.^[^ ^285^ ^]^ However, due to the high rate of pyrolysis, secondary reactions of volatile organic components to form carbon layers cannot be avoided. The presence of localized hot spots promotes the conversion of organic products to small molecules and non‐condensable gas products. The internal hotspots of biochar may also serve as a driving force for the diffusion of pyrolysis gases to the outside (pumping effect). The increased temperature difference between the inside and outside enhances the pumping effect and promotes the internal amorphous carbon to the rich porous structure of graphitic carbon. Thus the hot spots become active sites for graphitic carbon as shown in Figure 9c.^[^ ^286^, ^287^ ^]^ And more high‐value additional products (syngas, small molecule organic bio‐oil, etc.) appear during the preparation process.^[^ ^285^, ^288^, ^289^, ^290^ ^]^ Combining microwave‐assisted pyrolysis with preheating reaction is a good choice. Setting the preheating temperature at 350 °C, retaining a more complete carbon skeleton, and attenuating the water‐gas reaction during microwave pyrolysis can well increase the carbon content (>70%) and carbon retention (>50%).^[^ ^291^, ^292^ ^]^

#### 4.1.4 Laser‐Induced Carbonization

In addition to microwave pyrolysis, lasers are a means of generating very high energy in a short period to rapidly pyrolyze carbonization at high temperatures. Laser irradiation concentrates the high laser energy for a short period in a small area of ablation, causing destructive damage to the material. Any material that can be converted to amorphous carbon can be pyrolyzed into graphene by a CO_2_ laser beam.^[^ ^293^ ^]^ The laser pyrolysis process depletes the oxygen in the air at the surface of the material for a short period of time creating a special static low‐oxygen environment. Therefore the carbon structure in the center is not oxidized.^[^ ^294^, ^295^ ^]^ The laser‐induced high‐temperature localized rapid heating promotes polymer chain breakage and the aromatic backbone breaks and rearranges to form the *sp* ^2^ carbon structure. At pyrolysis temperatures between 400 and 450 °C, the hydrocarbon chains break/crosslink to release O_2_, CO, CO_2_, and other gases. As the temperature rises to 450 °C, the pyrolyzed material gradually releases NO_x_, NH_3_, and N_2_ gases. The growth direction of carbon fibers prepared by laser pyrolysis is along the direction of the laser beam. This special vertically oriented structure facilitates uniform penetration of the electrolyte and shortens the fast transport path of ions and charges. The fiber diameter is affected by the laser power. Medium power (0.76–0.86 W) has a uniform fiber diameter, whereas high power leads to the formation of inhomogeneous root‐like or agglomerated nanofiber structures by high‐temperature oxidation, as shown in Figure 9d.^[^ ^296^ ^]^ Due to the extremely high energy of laser radiation, pyrolytic materials should be protected from prolonged exposure. Failure to do so could result in structural damage or partial collapse and collapse. If the precursor contains metal atoms they will likewise aggregate due to prolonged laser exposure. So the laser scanning speed should be optimized. Using ZIF‐67 as a precursor, the scanning speed was controlled at 10 000 mm min^−1^. After pyrolysis, fragmentation, and cross‐linking a honeycomb network structure was formed. This layered porous structure facilitates close contact between electrolyte and electrode material.^[^ ^297^, ^298^, ^299^ ^]^

The high adiabaticity, medium orderliness, and high monomer dissociation of polyamides make them ideal precursors for laser pyrolysis. Laser pyrolysis produces an ionization plume on the material surface. The partial shielding effect of the ionization plume leads to further absorption of laser radiation by the material, potentially resulting in the accumulation of local temperatures above 2000 °C. The accumulation of higher temperatures leads to rapid graphene, and the C─N and C═O bonds in the oriented structure are rapidly broken and expelled throughout the process. The accumulated C─N and C═O bonds are dislocated and combined in the graphene sheet structure, enriching the surface functionalization of the nitrogen and oxygen heteroatoms of the CAC material. The graphitized carbon structure after pyrolysis improves resistance, mechanical stability, and precursor substrate adhesion.^[^ ^300^, ^301^ ^]^ When using poly(1,1‐difluoroethylene) (PDFE) as a precursor for localized laser heating, the incorporation of MXene contributes to fiber reinforcement and heat transfer. During pyrolysis, MXene lowers the carbonization temperature and forms microstructures along the fibers while accelerating the transformation of the graphitic structure of the carbon material. The final pyrolyzed carbon material exhibits high electrical conductivity, high mechanical stability, fiber integrity, consistent microstructure, and high carbon content. The properties of these materials meet the requirements for microdevices for smart skin bioelectronics and energy storage applications.^[^ ^302^ ^]^

#### 4.1.5 Molten Salt Carbonization

Low melting point salts or polysalts are used as useful media for the synthesis of a wide range of inorganic materials with a wide range of reaction temperatures. After mixing with CAC material precursors, they are heated and carbonized under an inert atmosphere and washed with water or dilute acid solutions to obtain the final porous CAC materials. Using biomass (wood chips) as the CAC precursor, the heat absorption from salt melting during pyrolysis mixed with salt is offset by the exothermic heat of the wood chips. The peak of heat absorption due to salt volatilization occurs at 710–770 °C. Thus, the molten salt medium remains stable up to 700 °C without significant evaporation losses. When the temperature is cooled down to room temperature, the molten salt‐carbon mixture splits into two phases due to salt recrystallization, so the molten salt can be recycled several times.^[^ ^303^ ^]^ The molten salt is also easy to handle or recover without using strong acids or bases in the whole process. Molten salt pyrolysis is therefore a green, sustainable, and easily scalable method.^[^ ^304^, ^305^ ^]^

The structural properties of the carbon products are similarly affected by the operating parameters. Molten salts interact with the carbon as an ionic liquid in violent thermal motion to form pores, and reactive gases released during pyrolysis act as activators to create a large number of pores. At controlled pyrolysis temperatures below 675 °C, the combination of pyrolytic melting of the organic material and pore creation by the inorganic material acting as a hard template produces thin‐walled, widely spaced sponges of carbon.^[^ ^306^ ^]^ At temperatures above 675 °C, the molten eutectic carbon‐containing mixtures act as a liquid sealing layer to prevent combustion of the carbon products in air, impede the release of volatiles, and increase carbon yields. Trace air infiltration into the molten salt during pyrolysis promotes the formation of oxygen‐containing functional groups that provide reactive sites, as shown in Figure 9e.^[^ ^303^, ^305^, ^307^, ^308^ ^]^ At controlled high salt/precursor ratios, the fast‐flowing molten salts interact strongly, and the carbon peels off into clumps and solidifies/carbonizes. The material separates from the molten salt phase to form mesopores/macropores.^[^ ^303^ ^]^ Residual salt clusters are likewise observed in the micropores. The carbon intermediates partially oxidize with the oxygen salts in the molten salt system to form pores. Thus the salt clusters act as pore‐forming agents and the size of their content influences the size of the carbon pores. However, an excess of molten salts can deteriorate the carbon structure causing the carbon pore walls to collapse. If the salt/precursor ratio is kept low, the dispersed salt acts as a template in the micropores causing the material to produce microporous carbon particles.^[^ ^309^, ^310^ ^]^ The separation of the carbonized and salt phases is facilitated by the addition of excess alkali metal chloride to alter the polarity/viscosity of the molten salt, increasing the ratio of mesopores to macropores.^[^ ^311^ ^]^

In the preparation of CAC by chemical activation method, molten salt is used as the reaction medium to enhance the activation reaction. The generation of pores by chemical activation and the generation of carbon particles by the template effect of the molten salt is carried out simultaneously to achieve the output of highly porous CAC material. This also means that explosive nitrates or sulfates/thiosulfates can be used as activators to prepare N‐ or S‐doped porous carbon, which provides a simple method for preparing doped porous carbon.^[^ ^312^ ^]^ Notably, electrochemical graphitization in molten salt to convert amorphous carbon (carbon black, carbon microspheres, carbon microfibers, and coke) into graphitic materials is a new method of preparing graphitic carbon at relatively low temperatures (850 °C). Amorphous carbon materials are removed from amorphous carbon materials by cathodic polarization in a molten salt environment to remove heteroatoms (O). Defects on the carbon surface then rearrange the disordered carbon atoms in the graphite lattice to form a scaled graphite structure. Catalytic electrolytic graphitization in molten salt is processed at lower temperatures, with higher crystallinity, graphitization and purity, and relatively low energy consumption.^[^ ^313^ ^]^

### 4.2 Activation

The specific surface area and pore structure of biochar obtained by pyrolysis/hydrothermal carbonization of biomass materials alone are not ideal for applications in pollutant treatment, and electrochemical energy storage applications. Therefore, after the high‐temperature carbonization of biomass materials to obtain non‐porous/less porous solid CAC materials with high carbon content and low oxygen and hydrogen content, the CAC materials can be appropriately modified by physical/chemical activation treatment. After activation treatment, more specific surface area, increased porosity, and richer surface functional groups were found in the CAC materials. Physical activation requires a high‐temperature environment (600–1200 °C) and the whole process is easy to implement and environmentally friendly. In contrast, chemical activation is usually carried out at low temperatures with short reaction times. Compared to physical activation, the final carbon product after chemical activation treatment requires additional impurity removal and wastewater treatment processes.

#### 4.2.1 Physical Activation

Physical activation is usually carried out in a temperature‐controlled tube furnace. The first step of pyrolytic carbonization is carried out in a neutral atmosphere with the pyrolysis temperature controlled at 400–850 °C. The catalyst precursor is then activated in an oxidizing gas (e.g. steam, carbon dioxide, carbon dioxide, and nitrogen or air mixture) and the pyrolysis temperature is raised to 600–1200 °C. Physical activation is the simpler method of activating biomass‐derived CAC. At the end of the pyrolysis process, the tar decomposed into disorganized carbon clogging the pores and reducing the specific surface area. The activation gas was controlled at high temperature to promote further calcination and decomposition of the CAC material to further develop the porous structure into interconnectable pore structure. Still, steam and CO_2_ are less corrosive than chemical activators and pore generation in porous carbon relies on oxidation reactions (Equations 24 and 25) that occur in an oxidizing atmosphere (H_2_O and CO_2_).

(24) $$C+H_{2}O\to \mathrm{CO}+H_{2}$$

(25) $$C+CO_{2}\to 2\mathrm{CO}$$

During physical activation, oxidizing gas molecules react preferentially with the solid carbon surface before gradually penetrating into the core region. This heterogeneous gasification occurring on the carbon particles and microzonated carbon surfaces inevitably leads to undesirable pore development and activation yields.^[^ ^314^ ^]^ Correspondingly, the activation time for physical activation needs to be enhanced to obtain a rich pore structure. Although the whole process consumes high energy, it is less corrosive to the production equipment. Physical activation is more suitable for practical application as a green and inexpensive method for CAC preparation.^[^ ^315^ ^]^

**Figure 10:**
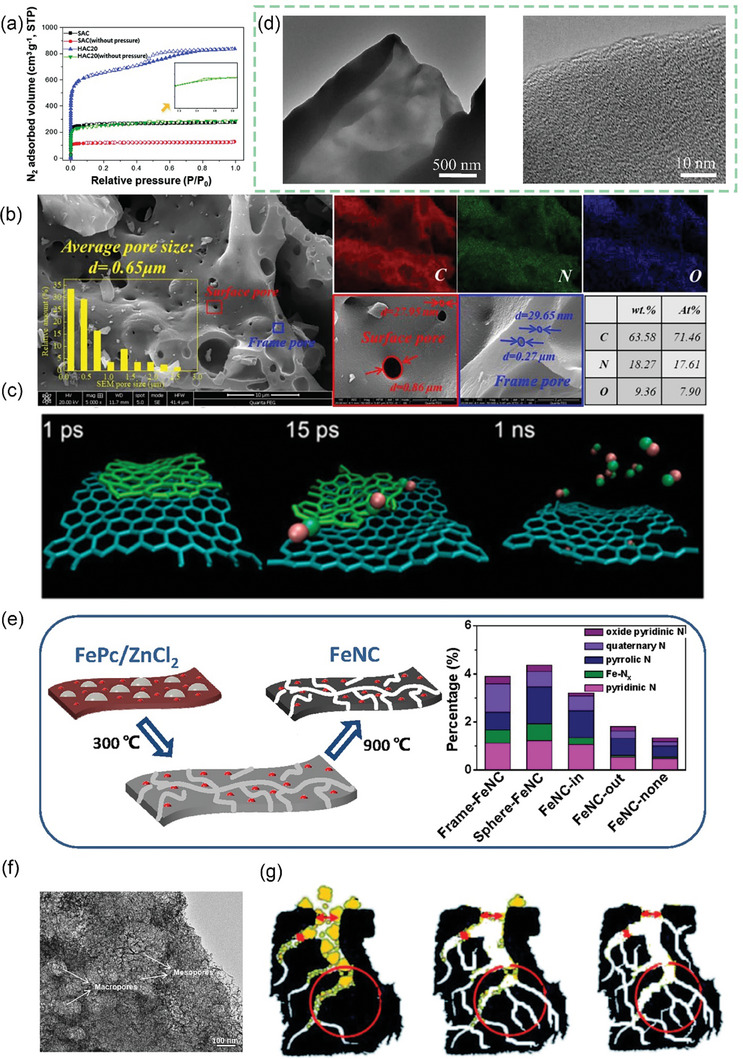
a) SAC and HAC20 with or without pressure as a function of the H_2_O_2_/steam ratio.^[^ ^317^ ^]^ Copyright 2024 Copyright Clearance Center, Inc. All rights reserved. b) Scanning electron micrograph and energy dispersive spectrum result of CS‐NH_3_·H_2_O‐800G.^[^ ^319^ ^]^ Copyright 2020 Elsevier B.V. All rights reserved. c) Representative snapshots in ReaxFF molecular dynamics simulations of CO_2_ activation of carbon: One finite nanographene layer on the surface of one graphene layer with indefinite size and the reaction results after 15 ps and 1 ns.^[^ ^323^ ^]^ Copyright 2017, American Chemical Society. d) TEM image and high‐resolution TEM image of C_PVA‐Mg(AC)2‐a_.^[^ ^328^ ^]^ Copyright 2017 Elsevier B.V. All rights reserved. e) The branch structures constructed by melt ZnCl_2_ well separate the adjacent Fe to avoid their agglomeration, Comparison of the absolute content of the different N species for the prepared materials.^[^ ^337^ ^]^ Copyright 2020 Dalian Institute of Chemical Physics, the Chinese Academy of Sciences. Published by Elsevier B.V. All rights reserved. f) TEM image of HPC‐700.^[^ ^348^ ^]^ Copyright 2019 Elsevier B.V. All rights reserved. g) CAC prepared by ZnCl_2_ activation (AC‐Zn); CAC prepared by ZnCl_2_ and KOH activation (AC‐K); CAC prepared by ZnCl_2_, KOH and water vapor activation (AC‐W).^[^ ^352^ ^]^ Copyright 2024 Copyright Clearance Center, Inc. All rights reserved.

When steam is used as the oxidizing gas for activation, the surface carbon atoms react with the steam molecules and the oxy and hydroxyl groups play a key role in forming the required pores.^[^ ^316^ ^]^ Considering that the permeability and reactivity of vapor molecules are not ideal, pressurization can be used during the activation process to enhance the diffusion of the activator thereby increasing the collisions between the surface carbon atoms and the activator. The conversion of water vapor into another effective and environmentally friendly oxidant (H_2_O_2_) can also be considered. At high temperatures, H_2_O_2_ can be easily converted into two hydroxyl groups, thus shortening the reaction time and increasing the reactivity to form a porous CAC material. **Figure** **10**a shows that the increase in collision frequency after re‐pressurization with starch as the CAC precursor increases the activation efficiency.^[^ ^314^ ^]^ Controlling the activation ratio of H_2_O_2_ also promotes the generation of new pores with oxygen‐rich functional groups and the expansion of pore size. Therefore, H_2_O_2_/steam activation becomes an efficient and environmentally friendly method for the top‐down preparation of CACs.^[^ ^317^ ^]^ CAC made by activation with NH_3_·H_2_O and H_2_O has graded pores and O/N functional groups. By this activation method, the carbon atoms in the amorphous region are gradually vaporized into graphite‐like microcrystalline regions. The carbon material forms a pore structure dominated by micropores and mesopores.^[^ ^318^ ^]^ In the two‐step pyrolysis method, some meso‐ and micropores formed in the first pyrolysis step are further broadened in the subsequent gas‐activated pyrolysis process. The corresponding specific surface area and pore volume of the carbon material are increased. During the activation reaction, the free NH_3_ molecules will compete for the reaction sites of H_2_O molecules. Therefore, the NH_3_ molecules will sacrifice part of the cavitation and etching effect of the H_2_O molecules, and the specific surface area of the resulting CACs is not as large as that of the CACs treated with H_2_O molecules only. However, the increase in the content of nitrogen‐containing functional groups facilitates the application of CO_2_ and NH_3_ adsorption, as shown in Figure 10b.^[^ ^319^ ^]^ Ammonia/vapor activation not only promotes carbon activation but also increases the concentration of combustible compounds (CO and H_2_) in the exhaust gas. Combustion treatment is therefore possible in industrial applications to reduce costs and recover gasified carbon and heat.^[^ ^318^ ^]^

In addition to H_2_O, CO_2_ gas can also control the gasification of CAC materials at high temperatures and is a common physical activator. Moreover, CO_2_ can be used as a carbon scavenger and the use of CO_2_ activation is beneficial to reduce carbon emissions. CO_2_ activation can reduce the formation of tar and avoid the carbon structure from clogging the pores, ensuring that the resulting catalyst has good porosity and surface area.^[^ ^320^ ^]^ The activation gas CO_2_ reopens the pore channels by accelerating the cleavage of condensable hydrocarbons such as tars and volatile organic carbons (VOCs) that are clogging the pores. H_2_O reacts with small molecules of hydrocarbons in the CAC to remove pore blockages. However, the production of CO and H_2_ accelerates the consumption of H_2_O, and therefore CO_2_, among the reactive gases, promotes the development of CAC pores better than H_2_O, and CO_2_ activation promotes the formation of aromatic and graphitic structures in the biochar to enhance the resistance of CAC to chemical and thermal oxidative decomposition. Therefore, CO_2_ activation has a better enrichment effect than H_2_O on carbon stability in CAC.^[^ ^321^ ^]^ According to the developed degree of pore structure and connectivity of CAC material, the reaction between carbon dioxide gas and carbon particles proceeds gradually from the outside to the inside. The degree of oxidation inside and outside of CAC material is relatively different. The pore diameters of mesopores and macropores formed during low‐temperature activation gradually increase with the increase of temperature.^[^ ^322^ ^]^ As shown in Figure 10c, carbonization residual atoms attack the substrate to produce carbon defects. The subsequent CO_2_ activation process of the CAC material first reacts with the graphite layer. Vacancies in the graphene substrate promote further activation to thin the defective graphite layer to produce micropores. As a result, the defective portion of the localized structure is effectively removed.^[^ ^323^ ^]^ In a series of V‐doped layered hydroxide‐derived Ni_x_–V–MgAl catalysts prepared and applied to the dry reforming of methane (DRM) reaction, the activation of CO_2_ can be accelerated by the reaction of CO_2_ with oxygen species on the surface of the carrier to form monodentate carbonates as active primary intermediates. However, multidentate carbonates are formed due to the strong interaction between CO_2_ and the support. Therefore, it is necessary to control the increase of monodentate carbonates by adding a V promoter to increase the CO_2_ concentration. V doping reduces the polydentate carbonates to prevent over‐strong adsorption and thus reduces the degree of CO_2_ activation, thus achieving a balance between CO_2_ adsorption and activation.^[^ ^320^, ^324^ ^]^ The catalyst prepared by using starch as a precursor undergoing dehydration and decarboxylation reactions during lyophilization and CO_2_‐activated carbonization had reduced oxygen content (containing only a small amount of oxygens). But the presence of a widely distributed mesoporous structure provided a total pore volume of 0.91 cm^3^ g^−1^ and a specific surface area of 1143 m^2^ g^−1^ for the catalyst, with the surface area of the micropores being as high as 490.8 m^2^ g^−1^. The presence of mesopores provides for the physical adsorption of HgO and the presence of mesopores provides conditions for the physical adsorption and rapid transport of HgO in the pore channels, while micropores are the active sites for the adsorption of HgO.^[^ ^251^, ^325^ ^]^

#### 4.2.2 Chemical Activation

The organic precursors are impregnated with chemical substances and oxidized and then highly dehydrated to reach chemical substance saturation. The mixture obtained after drying the suspension is activated by heating at a temperature of 400–900 °C and washed repeatedly to obtain the CAC. For this reason, chemical activation is also called wet oxidation.^[^ ^247^, ^326^ ^]^ Compared with the physical activity of carbonization and activation, which requires two furnaces, chemical activation requires only one furnace to complete the whole process. Moreover, chemical activation has a lower temperature, shorter processing time, and higher efficiency. The pore structure of the carbon product obtained is more advanced and controllable. However, due to the corrosive nature of chemical activators, the carbon products obtained after high‐temperature pyrolysis treatment need to be washed repeatedly to remove the spent activators. The cleaning step is time and resource intensive. Moreover, the toxic wastewater generated can pollute the environment requiring secondary treatment.^[^ ^327^ ^]^ Different types of chemical activators react differently with the precursors, and the pore size structure of the prepared CAC is different. In general, activators and precursors can be physically mixed under dry conditions and impregnation conditions. Common chemical activators are basic groups such as potassium hydroxide (KOH), sodium hydroxide (NaOH), calcium chloride (CaCl_2_), and potassium carbonate (K_2_CO_3_), acidic groups such as phosphoric acid (H_3_PO_4_) and sulfuric acid (H_2_SO_4_), intermediate metal salts such as ZnCl_2_ and other activators.

During carbonization of the dried KOH‐impregnated biomass precursor in an inert gas, the reaction between the carbon in the precursor and the KOH is first a solid‐solid reaction and then a solid‐liquid reaction. The process is completed by the reduction of potassium compounds to the metal K, the oxidation of carbon to carbon monoxide and carbonates, and the generation of other reaction intermediates.

The etching of carbon substrate by KOH, the physical activation of carbon particles by H_2_O and CO_2_ from the activation reaction, and the washing away of metal K and K compounds from the carbon lattice all cause the generation and expansion of pores. As shown in Figure 10d, the embedding of potassium salts increases the defective or disordered structure and in this way expands the carbon layer space. Therefore, most of the CAC materials show a disordered turbo‐layer graphite structure with a large number of micropores and doped heteroatoms in the carbon shelf. In this regard, the doping of heteroatoms can improve the wettability of the CAC material in the electrolyte to increase the ion transport rate of the material. Ultimately, the CAC material obtains higher electrochemical double‐layer capacitor performance. The presence of micropores, on the other hand, allows the CAC material to rapidly diffuse the electrolyte into the pores at higher current densities. This structure facilitates the storage of a large number of electrons.^[^ ^328^, ^329^ ^]^ After the nitrogen‐rich CAC material is pre‐oxidized, the nitrogen functional groups in the carbon skeleton are exposed in large quantities. The nitrogen functional groups reacted strongly with K species to form a well‐developed pore structure when the carbon skeleton was activated and reconstructed by KOH. The resulting CAC material has excellent nitrogen content and high BET‐specific surface area. The additional asymmetric pseudocapacitance due to the structural features gives the CACs excellent rate and cycling properties.^[^ ^330^ ^]^ During the activation of nitrogen‐doped Fe catalysts with KOH, in addition to further improving the pore structure, the combination of K and N promotes the electron‐rich environment of Fe. An appropriate amount of residual K can help the carburization of Fe to stabilize the activity as soon as possible. However, an excessive amount of K will produce too many graphitic carbon deposits covering the active phase and lead to catalyst deactivation.^[^ ^331^ ^]^

Activation with H_3_PO_4_ is also a common choice. This method is characterized by high efficiency, low energy consumption, non‐toxicity and non‐volatility, and low generation of exhaust gas, etc. H_3_PO_4_ activation is expected to be a green method for the preparation of CAC. Phosphoric acid as an acid catalyst has strong dehydration and elimination effects on the hydroxyl groups of organic compounds. After a series of bond dissociation, hydrolysis, and dehydration condensation reactions, the biomass precursor forms a stable condensed carbon structure and retains as much carbon as possible. After acid and water washing to remove inorganic components, highly adsorbable activated carbon is formed on the inner surface of the CAC. The reaction of phosphoric acid with CAC precursors generates whether particles or volatiles are removed by the washing process, leaving spaces that become new pores or expand the original pore size.^[^ ^332^ ^]^ The CAC obtained by phosphoric acid activation using goat hair as a biomass precursor has a well‐developed microporous, mesoporous, and macroporous structure. This structure facilitates the rapid transport of ions and electrons. The multi‐sized pore structure characteristic of CAC material plays the role of a capillary when applied to the electrocatalysis of Li‐S batteries. The sufficient pore space ensures the dissolution of long‐chain polysulfides, sulfur loading, and polysulfide anchoring. Meanwhile, the pore space provides a good buffer for the subsequent volume expansion. The effective suppression of the “shuttle effect” in Li–S cells greatly optimizes the electrochemical performance.^[^ ^222^ ^]^ The high polarity of phosphoric acid determines the importance of tuning the physical and chemical interactions by controlling the ratio of the solution body to the substrate. Increasing the impregnation ratio resulted in the gradual development and enlargement of micropores into mesopores thereby increasing the surface area of the mesopores.^[^ ^333^ ^]^ In addition to adjusting the impregnation ratio, the copper powder can be introduced during the activation process to make the dangling bonds on the precursor produced by phosphoric acid activation combine with the copper atoms in the electronically unsaturated state and the unsaturated bonds of the organic compounds to accelerate the pyrolysis reaction. However, it should be noted that excessive amount of copper powder may reduce the BET surface area due to the clogging of the pore structure and the conversion of mesopores into micropores.^[^ ^334^ ^]^

The preparation of biochar by activation with ZnCl_2_ is a mild chemical activation method. ZnCl_2_ does not react with carbon throughout the reaction. However, ZnCl_2_ can be used as a dehydrating agent and Lewis acid to enhance the carbonization of the precursor and the condensed aromatic reaction resulting from the hydrogen deformation of the molecules of the hydroaromatic structure in the precursor. The precursor undergoes deoxidation after the reaction reaches high temperatures, resulting in a decrease in the amount of hydrogen and oxygen and an increase in the amount of nitrogen in the material. Therefore, the CAC obtained by this method has a higher yield than that produced by KOH.^[^ ^335^ ^]^ Controlling the concentration of ZnCl_2_ allows controlling the CAC pore size from micro‐scale to mesoscale.^[^ ^336^ ^]^ The preparation of biochar using ZnCl_2_ activation is a mild chemical activation. Throughout the reaction, although ZnCl_2_ does not react with carbon, it acts as a dehydrating agent and a Lewis acid to enhance the carbonization of the precursor and the condensation aromatic reaction caused by hydrogen deformation of the hydrogen‐aromatic structural molecules in the precursor. Upon reaching high temperatures, the precursor undergoes a deoxygenation reaction to reduce the hydrogen and oxygen content. Encapsulation by ZnCl_2_ protects nitrogen‐containing small molecules from high temperatures. As shown in Figure 10e, the encapsulation effect of ZnCl_2_ can increase the nitrogen content in CAC materials by preventing small nitrogen‐containing molecules from escaping at high temperatures.^[^ ^337^ ^]^ Before the temperature reaches 283 °C, ZnCl_2_ occupies the carbon substrate as a template. After the temperature reaches 283 °C, ZnCl_2_ softens and melts and rapidly penetrates into the pores or cracks.“ The ZnCl_2_ occupying the ”carbon matrix is removed by high temperature and acid washing, but new jointed pores and cracks are retained. The new pores and cracks that are preserved help to increase the ion transport and diffusion rate and reduce the spatial resistance.^[^ ^338^ ^]^ Moreover, the CAC obtained by this method has a higher yield than that produced by potassium hydroxide. Controlling the concentration of ZnCl_2_ can control the CAC pore size from microscale to mesoscale. At temperatures above 732 °C, the specific surface area of the CACs was increased and more active sites were exposed by removing ZnCl_2_ by evaporation to produce more micro‐ and mesopores.

Conventional chemically activated synthesis strategies can severely corrode reactors. The reprocessing of acid/base contaminants increases the production costs and there is still a risk of environmental contamination. The final CAC produced has a small pore size and even the pore structure collapses due to high temperatures. These facts are indisputable and it is imperative to find new effective chemical synthesis strategies.^[^ ^327^, ^339^ ^]^

Inspired by KOH activation, alkali metal carbonate and bicarbonate activation reactions are gentler, less toxic, and corrosive.^[^ ^340^ ^]^ Take K_2_CO_3_ activation as an example, under re‐inert conditions, K_2_CO_3_ is reduced to K, K_2_O, CO_2_, and CO. Potassium vapor penetration into the graphene layer causes the carbon microstructure to expand and rupture. Both the potassium compounds that penetrate into the carbon matrix and the escaping generated gases cause the pores to expand and create new pores. Most CAC materials exhibit abundant microporous structures, and changing the addition of K_2_CO_3_ can adjust the pore parameters of CAC.^[^ ^341^, ^342^ ^]^ Compared to KOH, K_2_CO_3_ activation maintains the original morphology of the precursor and increases the carbon content.^[^ ^343^ ^]^ Similarly, the decomposition gas released from other decomposable salts by heat can also reach the pore formation by etching carbon, such as zinc acetate, Zn(NO_3_)_2_, NaNO_3_, calcium acetate, sodium acetate, MgCO_3_, sodium chloroacetate, K_3_PO_4_, NaH_2_PO_4_, tetraethyl orthosilicate, and potassium phthalate. Among them, zinc salts can act as dehydrating agents by trapping H_2_O molecules of organic carbon sources to promote the formation of double bonds between carbon atoms. The Zn(NO_3_)_2_·6H_2_O combustion reaction generates a large amount of high‐temperature gases during the activation reaction. In addition to the generation of volatile gases, the zinc oxide generated at temperatures up to 800 °C becomes embedded in the carbon matrix and reduces to liquid metallic zinc that penetrates the carbon matrix and diffuses. Eventually, most of the metal will be evaporated at 900 °C, and the residual Zn will be washed off by acid.^[^ ^344^ ^]^ Using phenolic resins (PFs) as the CAC precursor and Zn(NO_3_)_2_·6H_2_O as the hard template, combined with the soft template polyvinyl butyral (PVB), the mixture was directly carbonized under argon at 1000 °C. The final carbon product obtained had a total BET surface area of 864 m^2^ g^−1^ and a total pore volume of 0.76 cm^3^ g^−1^. The soft/hard templates used can be easily removed by an evaporation process, resulting in pure carbon without any post‐treatment.^[^ ^345^ ^]^

ZnCl_2_ was previously analyzed as a dehydrating agent involved in the activation of CAC materials, and the same is true for corrosive molten salt chemicals such as CuCl_2_, NiCl_2_, NaCl, KCl, and FeCl_3_ that can also react with carbon to generate porous structures. The use of molten salts for activation is a milder means of activation than traditional chemical activation, but their high cost is an obstacle to advancing practical applications. In the preparation of CAC for SO_2_ and H_2_S adsorption, CuCl_2_ was used as an activator to achieve in situ impregnation of copper. Because of the presence of copper and other surface functional groups chemisorbed SO_2_ and H_2_S, CAC prepared by ZnCl_2_ activation has a lower adsorption efficiency.^[^ ^346^ ^]^ When CAC was prepared by co‐activation with FeCl_3_ and CuCl_2_, the introduction of Cu enhanced the strong interaction between Fe and N. The electronic structure was changed. The ORR performance was enhanced by changing the electronic structure of the carbon matrix, and the Fe compounds enhanced the graphitization, allowing the carbon atoms to dissolve into the catalyst after graphitization precipitation. The result is a carbon structure with high porosity and abundant active site formation that possesses excellent ORR catalytic activity. Thus the synergistic effect of Cu and Fe‐N‐C modulates the electronic/microstructure ultimately achieving high ORR activity, excellent long‐term stability, and excellent methanol tolerance of CAC catalysts.^[^ ^347^ ^]^

Modulation of CAC materials to the desired pore size structure can also be achieved using oxidation activators at room or high annealing temperatures, such as HNO_3_, KMnO_4_, KNO_3_, and Mn(NO_3_)_2_. Potassium permanganate, with strong oxidizing properties, reacts with the reducing groups (─OH and ─NH_2_) on chitin (precursor) during the activation process to break the hydrogen bonds of the long chain of chitin. Potassium salt and manganese dioxide generated during the reaction process were used as templates embedded in the skeleton to form macropores and mesopores. The activator was pyrolyzed at high temperature to form micropores and mesopores. The final catalyst carbon matrix has a layered porous structure as shown in Figure 10f.^[^ ^348^ ^]^ After the nitrogen‐doped CAC is etched by KMnO_4_, in addition to generating a large number of mesoporous structures and defects, oxygen‐containing functional groups are also introduced at the edges. After high‐temperature pyrolysis, some oxygen‐containing functional groups on the material are reduced as well as the graphitization of the carbon material is reduced. As a result, the nitrogen dopants on the *sp* ^2^ carbon backbone are exposed. The C═O and ─COOH generated by the oxidized carbon precursor after KMnO_4_ will promote the generation of pyridine N. The final pyridine N‐rich and defective CAC material is obtained with high ORR activity.^[^ ^349^ ^]^

#### 4.2.3 Combination of Chemical and Physical Activation

After initial chemical activation by activators to obtain a microporous‐rich carbon structure, physical activation was then performed to develop or adjust the initial pore size structure to finally prepare a modified CAC with high porosity and surface area.^[^ ^350^ ^]^ The lignocellulosic materials (coconut shell and palm) were activated by ZnCl_2_ or KOH followed by CO_2_ activation. This coupled activation design enhances the generation of mesopores. With the variation of activation conditions (ratio of activation to precursor, impregnation time, and pyrolysis temperature), the BET surface area of CAC material could exceed 2100 m^2^ g^−1^ and the mesopore content (mesopore volume to total pore volume) of the carbon structure was 71%. In addition, palm‐based CAC had a high mesopore content of 94%.^[^ ^351^ ^]^ Using coconut shell as the biomass precursor, the biomass precursor was first activated by zinc chloride to generate mesopores in the carbon skeleton. Then further activation of the carbon material was carried out using more etching effect KOH to enrich the number of micropores and connect the mesopores while removing impurities and non‐carbonate particles from the pores. Finally, the carbon material was water vapor activated at high temperature to further remove impurities and maintain the connectivity of the pore structure, as shown in Figure 10g. After three activations, the biomass material was finally transformed into a CAC material with a 3D interconnected network structure. The abundant and interconnected microporous and mesoporous structure of the material greatly reduces the resistance to ion transport and diffusion and improves capacitance. The abundant pore structure enlarges the reaction contact area of the carbon material and exposes more active sites. The application of this CAC material to capacitive electrodes has a low capacitance loss rate and also maintains good long‐term stability.^[^ ^352^ ^]^

In addition to the high porosity carbon structure obtained by treating the precursors with physical‐chemical activation, the surface chemistry can be improved by adding a process of surface treatment with oxidants (HNO_3_, H_2_SO_4_, and H_2_O_2_). After the introduction of the modification treatment, the resulting surface functional groups will reduce the pore size and even block some of the pores. So the surface area and pore volume of the modified CAC is reduced. The increase in oxygen‐containing functional groups gives CAC a partial negative charge. Increasing the electronegativity of the material enhances the polarity of the CAC material making it more hydrophilic. The modified carbon electrode material has higher capacitance, good reversibility, and high stability.^[^ ^353^ ^]^ Sulphuric acid was used as a dehydrating agent and catalyst to break glycosidic bonds in cellulose and hemicellulose as well as aromatic groups in lignin during the reaction with corncob meal. The viscous material produced after activation by sulphuric acid ensures that the carbon material remains spherical during the subsequent pyrolysis process. The H_2_O, CO_2_, and CO gases generated during the pyrolysis process further activated the carbon materials to achieve the adjustment of the pore structure. The finally prepared self‐adhesive 3D spherical biochar achieved the highest power density (2066.7 ± 7.0 mW m^−2^) and high electroactive bacterial abundance (92.8%) as an anode for microbial fuel cells.^[^ ^354^ ^]^

#### 4.2.4 Self‐Activation

Whether it is physical or chemical activation, the pollution and waste caused by the corrosion of equipment and washing treatment during the reaction process are difficult to avoid. Therefore, the development of self‐activation methods that simplify the procedure, reduce costs, and reduce the risk of pollution has become a valuable research direction. The self‐activation effect was achieved by pyrolyzing organic acid salts containing Na, K, and Ca (ammonium iron citrate, potassium gluconate, sodium gluconate, sodium citrate, sodium alginate, potassium tartrate, sodium tartrate, zinc citrate, calcium citrate, iron, potassium citrate, trisodium citrate calcium citrate, iron, potassium citrate, trisodium citrate, mixtures of citrates, etc.) on carbon substrates to form metal nanoparticles and oxides as in situ templates.

Another approach, without any reagents, is called the biomass self‐activation strategy. The ability of biomass materials to achieve self‐activation may be due to the reaction of CO_2_ and H_2_O released during pyrolysis with carbonaceous materials. Or because the alkaline earth elements stored in the organism act as active agents in the activation reaction. Biomass self‐activation can be classified into physical and chemical self‐activation according to the type of reaction.^[^ ^355^, ^356^, ^357^ ^]^

Physical self‐activation exhibits conventional pyrolytic behavior at temperatures between 100 and 700 °C. As the temperature increases from 700 to 1000 °C, the self‐activation reaction takes away carbon atoms resulting in a decrease in carbon yield. The pore size and the content of other elements (O, H, N) in the carbon matrix also decrease with increasing temperature. According to the quality and yield of CAC, the optimum self‐activation temperature is 1000 °C. The specific surface area and pore volume of CAC are influenced by the holding time, showing a trend of first increasing and then decreasing with time.^[^ ^358^ ^]^

The central idea of chemical self‐activation is to transform the “harmful” metal impurities in the CAC precursor into activators in the preparation process.^[^ ^359^ ^]^ As a high‐quality, harmless, and renewable carbon source rich in alkaline earth elements, the carbon structure of grapefruit peels has a uniform distribution of dopant elements. Heteroatoms evolve into nanoparticles during carbonization and become sacrificial templates for volatilization during subsequent pyrolysis. This results in a large number of mesopores or macropores in the final carbon material obtained. In addition to enriching the pore structure, the presence of doped metals will promote graphitization during carbonization, and the increase of graphitization ensures effective charge transfer.^[^ ^360^ ^]^

### 4.3 Other Methods

In addition to the traditional activation carbonization methods for biomass materials to prepare ideal CAC‐based catalysts, researchers introduce other methods or techniques to enhance the catalytic performance of CAC materials or to impart special properties to CAC materials for special applications. Based on the original high chemical resistance to the decomposition of biomass materials and the inherent presence of recalcitrant structures that limit the utilization efficiency, various pretreatment methods promote high levels of biomass utilization. Pre‐carbonization of lignin increases the *sp* ^2^‐hybridized carbon bonds and thus extends the polycyclic aromatic structure as well as increases the mesopore volume in porous carbon. The evolution of the spatial structure of the carbon material can be inferred by examining the ratio of the content of *sp* ^2^‐ to *sp* ^3^‐hybridized carbon bonds in the pre‐carbonized lignin material. It is therefore feasible to precisely control the pore structure by modulating lignin inter/intramolecular bonding, a molecular‐level strategy.^[^ ^361^ ^]^ Acid pretreatment methods can dissolve hemicellulose, leading to changes in lignocellulosic biodegradability. Hydrolysis of Lewis acid (FeCl_3_) breaks the β‐1,4‐glycosidic bonds in cellulose and hemicellulose macromolecules in wood leading to reduced polymerization. Micro‐ and mesopores appear in wood‐based carbon materials treated with FeCl_3_. Fe^3+^ is retained by the chelation of oxygen‐containing functional groups in the cellulose/hemicellulose hydrolysis products and is coordinated to N atoms during pyrolysis. Therefore, the retention of Fe^3+^ facilitates nitrogen doping and the formation of monatomic iron in the porous structure of carbon materials.^[^ ^362^ ^]^ In addition, the “waste” from other biochemical technologies is also a pretreated biomass‐derived CAC precursor. Pretreatment of microalgae lipid extraction results in a CAC material with higher nitrogen content than the raw material, making it an ideal carbon source for N‐doped CAC materials. Moreover, the lipid extraction will result in a richer nano‐ and mesoporous structure, effectively increasing the specific surface area and enhancing the ORR activity.^[^ ^101^ ^]^

The modification of biomass‐derived CAC materials by ball milling is a kind of mechanical force chemistry, mainly through the kinetic energy generated during the ball milling process to break the chemical bonds of related molecules and generate free radicals to obtain the target properties or add functional groups to the biochar. In addition, the biochar material after ball milling is fine particles with a diameter of 100 nm, so a large specific surface area is obtained. Moreover, the content of *sp* ^3^‐C and the defective structure will be increased during the grinding process due to the breakage of C═C bonds, thus introducing oxygen‐containing functional groups and contributing to the increase of catalytic activity.^[^ ^93^, ^363^, ^364^ ^]^

The stencil method is a common method for modulating the micro‐ and macro‐structure of porous materials according to the need and is divided into hard and soft stencil methods depending on the structure of the stencil itself and the connection between the subjects. Hard templates are obtained by coating carbon precursors onto the template and then etching the template using acid or alkali, resulting in a CAC material with a fixed shape and size and the ability to synthesize a narrow pore size distribution. The CAC material obtained by one‐pot pyrolytic carbonization of defatted soybean residues in the combined presence of potassium oxalate (activator) and calcium sulfate (template method) has a hierarchical porous structure of interconnected large cavities, leading to a denser structure and larger specific surface area.^[^ ^365^ ^]^ The nitrogen‐doped hierarchical porous carbon is synthesized using crayfish as a bio template and carbon skeleton with heavy bio‐oil, and the 3D interconnected hierarchical porous structure provides electrolytes with smooth channels and reduces diffusion distance and diffusion resistance.^[^ ^366^ ^]^ Then, for example, two hard templates (Mg_5_(OH)_2_(CO_3_)_4_ and ZnCl_2_) were mixed with different biomass precursors (roots, stems, leaves, flowers, and fruits of common plants) were successfully synthesized as multilevel porous heteroatom‐doped CAC materials.^[^ ^367^ ^]^ The soft template method controls the material morphology by forming an ordered microstructure of amphiphilic molecules in solution, the size of the template can be controlled to regulate the narrowest pore size distribution of the CAC material, and the soft template can be removed by pyrolysis under inert atmosphere. The CAC materials obtained by using sweet potato as the carbon precursor and F127 as the soft‐templating agent have perfect interconnected carbon spheres of relatively uniform size and a complex pore structure including macro‐ and mesopores, meaning that the use of soft‐templating increases the specific surface area and porosity of the CAC materials.^[^ ^368^ ^]^

### 4.4 Summary of Synthetic Strategy

The decomposition of organic macromolecules and the generation of reaction gases during the carbonization of biomass precursors with increasing temperature accelerate the construction of pore structures, but too high a temperature can cause the destruction or enlargement of microporous structures and is not conducive to improving the reaction activity. At the same time, the nitrogen‐doped in biomass also changes its form of existence with temperature, and the different forms of nitrogen exist to form active sites by changing the charge distribution of surrounding carbon atoms. The size of the pyrolysis rate determines the adequacy of the biomass pyrolysis reaction and the final carbon yield. The choice of the pyrolysis atmosphere will be determined according to the catalyst design needs, with the flow of inert gases ensuring no reaction with precursors during the pyrolysis process, physical activation gases increasing the catalyst reactivity, and the introduction of NH_3_ being used as a nitrogen source to induce the generation of nitrogen‐doped carbon. The high catalytic performance of biomass‐derived CAC‐based catalysts can also be obtained by designing a reasonable structure from the catalyst itself or by combining other experimental tools/techniques with appropriate pyrolysis parameters. Hydrothermal carbonization, as a branch of conventional pyrolysis, abandons the pyrolysis atmosphere parameters and produces CAC from the physical and chemical reaction of biomass precursors in a solvent by controlling the hydrothermal temperature and residence time only. Although hydrothermal carbonization offers energy savings and higher carbon yields than pyrolytic carbonization, while avoiding the risk of gas contamination, the controlled adjustment of carbon structure during the hydrothermal process is a major challenge. The emerging microwave pyrolysis and laser pyrolysis, characterized by rapid pyrolysis, have the advantages of high efficiency and low energy consumption compared with traditional pyrolysis, so once the pyrolysis time is too long, it will lead to structural collapse, and the feasibility of precise regulation of heating rate and temperature is low from the heating principle. Molten salt carbonization and hydrothermal carbonization are both wet chemical synthesis methods, but molten salt carbonization has a higher operating temperature than hydrothermal carbonization, so biomass in molten salt will get a higher degree of carbonization and introduce new morphology and structure. Considering that molten salts are solid phase at room temperature, it is better to crush the biomass precursors and mix them with molten salts before proceeding to the next steps to achieve better results, and this also means that controlling the material homogeneity is the difficult part of the technique. The control of heating time, heating rate, and heating medium during the carbonization of biomass materials is based on the changes in microchemical composition and macroscopic carbon skeleton structure with energy input to ensure precise and controllable preparation of catalysts. The trend is to combine the carbonization process with the structural modification and activation of biomass materials to reduce the process. While ensuring that the structure and properties of the final CAC are in accordance with the experimental design, time and energy saving should be pursued in order to reduce the cost of the catalysts prepared by large‐scale synthesis.

Activation of biomass precursors is usually required to obtain a large specific surface area, multi‐level pore structure, and abundant surface functional groups to enhance reactivity, compensating for the high carbon, low hydrogen, low oxygen, and non‐porous/low pore size of the material after carbonization only. Whether physical activation utilizing oxidizing gases or chemical activation using chemical reagents, the reaction on the carbon particles or microtonal carbon surfaces promotes the development of porous structures into interconnected pore structures. The modulation of the pore structure by activation depends on changing the physical activation time or the impregnation ratio of chemical reagents to biomass precursors. On this basis, changing the oxidation gas or chemical activator to impart surface functionalization to the CAC material or introducing heteroatoms to change the charge distribution of carbon atoms to enrich the active sites is what has been explored. The search for activation methods does not stop at the development of richer activation media within each branch, and the combination of physical and chemical activation for double or even triple activation of biomass materials is a good attempt. Although the time cost is sacrificed, the CAC‐based catalysts eventually present rich and interconnected microporous and mesoporous structures as the best feedback. In addition, the use of self‐activation is a valuable research direction from a green perspective. Regardless of the activation method chosen, priority should be given to the feasibility and reproducibility of preparing catalysts with suitable pore size structures and improved surface chemistry before pursuing greener and more economical processes to promote large‐scale industrial preparation of catalysts. When performance and green economy are irreconcilable, the advocates of both are like the relationship between deep‐rooted and blooming, both have some research value, and only the coexistence of both can improve and solidify our research framework. Of course, it is the ultimate pursuit of all researchers to be able to balance performance and greenness while achieving high‐level results.

## 5 Strategies and Methods for Improving Catalytic Performance

Due to their abundant resources, easy fabrication, and stable physicochemical properties, biomass CAC materials have been viewed as ideal materials for electrocatalysts, but to meet the application requirements and improve the intrinsic activity of CAC‐based catalysts, it is necessary to rely on the introduction of heteroatom doping, defect engineering, single‐atom catalysts, and surface molecular functionalization to adjust the altered charge distribution, spin redistribution and electronic energy band structure among carbon atoms. Altering the physicochemical properties of carbon catalysts to achieve functionalized CAC materials serving related applications in catalysis, energy, and environmental technologies.^[^ ^62^ ^]^

### 5.1 Doping

Due to the poor catalytic effect of pure carbon materials in various catalytic applications, researchers further found that the application performance of materials can be greatly improved by modifying carbon materials. Heteroatom‐doped carbon materials have made great progress in the past decades, resulting in a very promising material concept. There are two main forms of heteroatom doping: one is substitution doping. Heteroatoms substitute one or more carbon atoms in *sp* ^2^ configuration; The other is that heteroatoms are fixed in the carbon framework, and no atoms are replaced. Heteroatom doping can be achieved by carbonizing the precursor of heteroatom‐rich compounds or by post‐treating CAC materials with reactive heteroatom sources. In metal‐air batteries and fuel cells, heteroatom‐doped carbon materials are promising and effective substitutes for platinum‐based catalysts for ORR electrocatalysis.^[^ ^79^ ^]^

#### 5.1.1 A Doping‐Driven Catalysis Mechanism for the Oxygen Reduction Reaction

Substitution of doped atoms such as nitrogen (N), boron (B), sulfur (S), phosphorus (P), fluorine (F), silicon (Si), and chlorine (Cl) on the carbon surface is an effective way to adjust the basic properties and improve the electrochemical properties. Heteroatoms are embedded in the carbon skeleton in a covalent and homogeneous form, leading to distortion of the *sp* ^2^ carbon network to form defects, the uneven charge distribution in the carbon framework, bending of the graphite layers, and widening of the interlayer spacing. When there is an electronegativity difference between the introduced heteroatom and the carbon atom, the heteroatom with high electronegativity will “grab” the electrons around the carbon atom, and the stronger the electronegativity of the heteroatom, the higher the density of positive charges around the carbon atom. In contrast, heteroatoms with low electronegativity will give carbon atoms electron density and make them negatively charged. By studying the structure‐activity relationship of N/P/S‐doped Pt‐based catalysts synthesized with different dopants, it can be seen that N atom doping increases the activity of active sites, S doping forms C─S bonds to improve the stability of catalysts, and the existence of platinum–phosphorus species results in adverse effects on the oxygen reduction process.^[^ ^369^ ^]^ It can be seen from the theoretical calculation results of the synthesis of metal heteroatom clusters with engineering coordination from carbon‐based materials doped with multiple heteroatoms that the strong electron traction from the center Fe to the surrounding N atoms forms a high degree of electron localization. The difference is that S atoms with electronegativity lower than N have weak electron absorption ability. In addition, electrons tend to delocalize among multinuclear Fe atoms, which results in higher electron density and decreased oxidation number of central Fe sites for Fe_2_−S_6_ and Fe_3_−S_5_N_1_. Notably, the N atom for stabilizing Fe_3_−S_5_N_1_ further adjusts the electronic structure and the asymmetrical charge distribution is favorable for promoting ORR kinetics.^[^ ^370^ ^]^ Therefore, the selection of a dopant atom with high or low electronegativity depends on whether the collective electronic band structure altered by doping enhances the interaction between carbon and nucleophilic/electrophilic reagents. The charge and rotational changes of carbon modulate the power function and the adsorption of reactants or intermediates at specific sites.^[^ ^371^ ^]^ When the introduced heteroatoms have similar electronegativity to the carbon atoms, the effect of doping effects can be considered in terms of atomic size. Large‐size heteroatoms are not avoided to create a large number of defects and strains in the carbon skeleton, the charge density (or spin density) symmetry is broken and the local dense electrons become active sites. And the distortion of the forming carbon skeleton and the widening of the graphite layer spacing effectively enhance the intercalation. All these changes can affect the performance of the reactivity.^[^ ^39^ ^]^

In doped CAC materials, the presence of heteroatoms changes the wettability of the surface of the material. Under aqueous electrolyte conditions, the formation of hydrophilic groups increases the wettability of the CAC material with water, allowing the CAC material to pass through the electrolyte solution more quickly when applied to electrode materials. Correspondingly, hydrophobic groups are applied to organic system electrolyte conditions, and such treatment application greatly improves the surface utilization of CAC material and increases the fast charging and discharging capability of the supercapacitor.^[^ ^79^ ^]^ In addition, the functional groups formed by the doped heteroatoms are a major contributor to the redox reaction of CAC materials, so the resulting pseudo capacitance expands the double‐layer capacitance of the original material, and consequently increases the capacitance of the capacitor, and its electrochemical catalytic performance is improved. The presence of the heteroatom itself reduces the internal resistance of the material and improves the electrical conductivity and conductivity, resulting in good multiplicity performance, cycling performance, and charge/discharge rate when applied to supercapacitors.^[^ ^371^, ^372^ ^]^

#### 5.1.2 Spatial Distribution of Heteroatoms

The spatial distribution of doped heteroatoms in the carbon structure is likewise a point worth exploring, and clarifying the effect of the spatial distribution of heteroatoms on catalytic activity will advance the understanding of the structure‐property conformational relationship.

**Figure 11:**
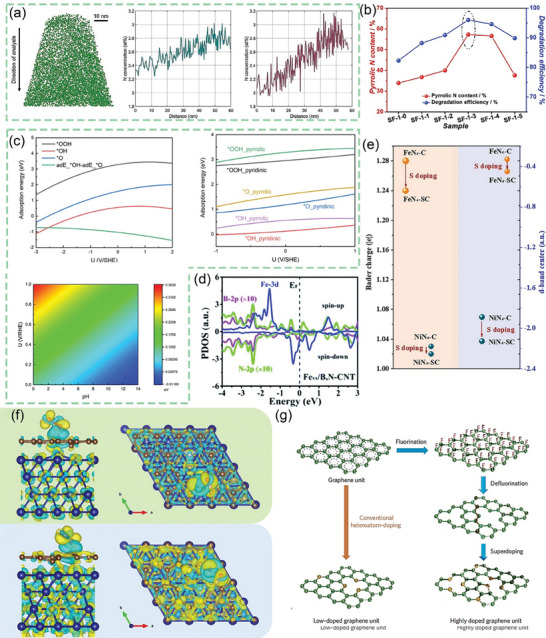
a) 3D atom‐by‐atom reconstruction of N atoms in T800. For clarity's sake, only 10% of the acquired N atoms are shown. N concentration profile of lateral specimens of T800 and IMS65. (A color version of this figure can be viewed online.)^[^ ^373^ ^]^ Copyright 2021 The Author(s). Published by Elsevier Ltd. b) The curves of the pyrrolic nitrogen contents and the degradation efficiencies of the samples of SF‐1‐0, SF‐1‐1, SF‐1‐2, SF‐1‐3, SF‐1‐4, and SF‐1‐5.^[^ ^119^ ^]^ Copyright 2019 Elsevier Ltd. All rights reserved. c) pH‐dependent and potential‐dependent contour plot of adsorption energies of *OH on pyridinic FeN_4_C. Adsorption energies of *OOH, *O, and *OH and the difference between *OH and *O as a function of the applied electrode potential. Adsorption energies of *OOH, *O, and *OH as a function of the applied potential on pyridinic FeN_4_C and pyrrolic FeN4C.^[^ ^386^ ^]^ Copyright 2022, American Chemical Society. d) The PDOS for the Fe‐3d, N‐2p, and B‐2p orbitals in Fe_SA_/B,N‐CNT.^[^ ^391^ ^]^ Copyright 2024 Copyright Clearance Center, Inc. All rights reserved. e) Trend of Bader charge and D‐band center after S doping.^[^ ^398^ ^]^ Copyright 2023 Elsevier B.V. All rights reserved. f) Charge density differences of the carbon layer in the catalyst models of NC‐Co and NSC‐Co, in which the Co, C, S, and N atoms are in blue, gray, yellow, and light white, respectively; the yellow and light blue areas represent charge accumulation and depletion, respectively.^[^ ^409^ ^]^ Copyright 2023 The Authors. g) Conventional heteroatom‐doping of the graphitic honeycomb network consisting of conjugated alternating C─C single and C═C double bonds with delocalized electrons (represented by the blue dotted rings; omitted in the subsequent structures for clarity). Carbon atoms are shown in green and dopant atoms in yellow.^[^ ^420^ ^]^ Copyright 2016, Macmillan Publishers Limited.

The 3D spatial distribution and chemical states of doped atoms can be interpreted using atom probe tomography (APT) and synchrotron hard X‐ray photoelectron spectroscopy (HAXPES). APT uses voltage or laser impingement from a highly localized power plant to determine the species based on the time of flight of individual ions and the relative position based on the ion impact location and combines the above information to obtain 3D reconstructions. Synchrotron‐based hard X‐ray photoelectron spectroscopy (HAXPES) has larger tunable X‐ray energy and therefore high throughput and high energy resolution to determine chemical states at different depths and to satisfy the measurement of chemical states of low‐content atoms. Detection using APT shows that the distribution of N atoms within the carbon fiber is smooth and non‐aggregated over a small range, but increases with volume depth as shown in **Figure** **11**a. HAXPES detection results can be seen as the graphite‐N content becomes deeper with material depth. The information detected by HAXPES can provide corroboration for the conclusions of APT, but some of the information does not agree with APT. The difference between the two techniques is that HAXPES detects a large area and the analysis volume is close to the surface, while APT detects the probe at the nanoscale and goes deeper into the material. The influence of the distribution and chemical state of the heteroatoms on the electrocatalytic performance can be inferred from the amount of each type of N atom in the different CAC materials and their electrocatalytic performance.^[^ ^373^ ^]^ The spatial distribution of heteroatoms can also be mapped on the nanoscale using aberration‐corrected scanning transmission electron microscopy and electron energy loss spectroscopy (STEM‐EELS). The technique can even capture the distribution of heteroatoms in gaseous soot particles.^[^ ^374^ ^]^

#### 5.1.3 Single‐Atom Doping

Nitrogen doping is a common choice among heteroatom doping strategies. Since nitrogen is adjacent to carbon in the periodic table, N can be used as an electron donor to provide electrons to the off‐domain carbon network to increase the conductivity, and the similarity of N and C atomic radii reduces lattice mismatch. N doping in the lattice inhibits hydrocarbon formation and avoids the formation of dielectric dead layers, and the C─N bond structure resembles N‐type semiconductor materials with faster ion and electron transport. The N content of the catalyst sample can be adjusted by controlling the mixing ratio of carbon and N source and annealing temperature during the N doping process.^[^ ^375^, ^376^ ^]^ Therefore, the optimal equilibrium active site density should play a “site isolation effect”, so that each N site maintains the optimal distance to play a beneficial benefit.^[^ ^377^ ^]^

The nitrogen doping configuration is divided into three types according to the position of the nitrogen‐substituted carbon atom: the first type of nitrogen atom replaces the carbon in the edge five‐membered ring and contributes two p‐electrons to the π‐electron system, this configuration is called pyrrole nitrogen. Pyrrole nitrogen belongs to *sp* ^3^ hybridization with a pair of lone electrons in the outermost layer. The second type of nitrogen atom replaces the carbon in the marginal six‐membered ring and contributes one p‐electron to the π‐electron system by bonding with two C atoms, and this configuration is called pyridine nitrogen. Pyridine nitrogen belongs to *sp* ^2^ hybridization and retains an aromatized off‐domain π‐bond with a pair of lone and unpaired electrons in the outermost layer and a large difference in electron cloud density. The third type of nitrogen atom replaces the carbon atom in the center of the three benzene rings in‐plane, providing 0.5 electrons to the π‐electron system, and this configuration is known as graphitic nitrogen. Graphitic nitrogen belongs to *sp* ^2^ hybridization with more homogeneous aromaticity and a lone pair of electrons outside the nucleus. Graphitic nitrogen belongs to n‐type doping, while pyrrole nitrogen and pyridine nitrogen take electrons from the π‐electron system and belong to p‐type doping.

Modulating the type of configuration of N atom doping based on different purposes is possible, and selectively building only one nitrogen configuration or increasing the proportion of a certain nitrogen configuration as much as possible is an important and interesting attempt to better understand the material properties of different nitrogen configurations. In the ORR, the pyridine nitrogen adjacent carbon atoms occupy regions near the Fermi energy level to maintain the local density of states so that the carbon atoms have Lewis bases and adsorb O_2_ electrons for the π* orbitals of the pyridine nitrogen, and the ORR, the pyridine nitrogen is protonated to form pyridine ions as new active sites.^[^ ^378^ ^]^ Interestingly, in the M‐N_5_ structure, pyridine nitrogen has access to net electrons, leading to a hole enrichment near M‐N_5_. The enriched holes contribute positively to all four ORR reaction steps.^[^ ^379^ ^]^ Nonetheless, when the concentration of pyridine nitrogen as the ORR active site is increased, the carbon atom Lewis basic site between two pyridine nitrogen sites is inactivated, so each pyridine nitrogen ratio activity is reduced instead.^[^ ^380^ ^]^ Moreover, in the ORR, the pyridine nitrogen is protonated to form pyridine ions as new active sites.^[^ ^75^ ^]^ Under an oxygen‐rich environment, increasing the pyridine nitrogen content should enhance the catalyst stability given that the pyridine nitrogen free energy is higher than that of pyrrole nitrogen and the bond length of pyridine nitrogen is shorter than that of pyrrole nitrogen.^[^ ^381^ ^]^ The hydrogen sites in pyridine nitrogen‐related structures provide low reaction potentials and high limiting potentials for the 2e^−^ ORR to form H_2_O_2_ at relatively low potentials under acidic conditions.^[^ ^382^, ^383^ ^]^

Different nitrogen configurations have different adsorption capacities for oxygen molecules and different effects on the electron density and spin density of adjacent carbon atoms, leading to different production and desorption of intermediate OOH*, while the nitrogen configuration affects the surrounding defective pores. The differences in these factors will interfere with the diffusion kinetics of H_2_O_2_ into the bulk phase. The intensity of intermediate adsorption was determined by the variation of peak intensity characterized by oxygen K‐edge XANES spectra, with strong C─OOH* peak intensity and weak C─O* peak intensity can be concluded that the catalyst has a high two‐electron oxygen reduction selectivity, and combined with the negative shift of pyrrole peak due to heterocyclic distortion caused by carbon atom adsorption in the nitrogen K‐edge XANES spectra while the position of other carbon configuration peaks remained unchanged, so the specific nitrogen configuration is correlated with hydrogen peroxide selectivity.^[^ ^384^ ^]^ When applying the 2e^−^ ORR to the EF degradation process, the pyrrole nitrogen content becomes critical, and it can be clearly observed from Figure 11b that the pyrrole nitrogen content has a positive effect on the EF degradation efficiency.^[^ ^119^ ^]^ At the same time, metal catalysts with high pyrrole‐nitrogen coordination content use M‐N_4_ sites with adjacent metal nanoparticles to modulate the electronic structure to achieve efficient bifunctional oxygen electrocatalysis by ORR and OER.^[^ ^385^ ^]^ The contribution of pyridine nitrogen and pyrrole nitrogen to the catalytic activity of oxygen reduction is still controversial, and it can be seen from Figure 11c that the adsorption intensity of the reaction intermediate *OH on pyridine nitrogen increases with increasing PH or decreasing applied potential, so pyridine nitrogen has PH dependence. Based on the X‐ray absorption fine structure and the N signal obtained by XPS can only determine limited local coordination information, and the pyrrole nitrogen model is more complex, so the contribution of pyrrole nitrogen to the catalytic activity is more ambiguous. However, it can be seen from Figure 11c that compared to pyrrole nitrogen, pyrrole nitrogen has a reduced intensity of adsorption for the three intermediates (*OOH, *OH, and *O), while the higher DOS values near the Fermi energy level than pyrrole nitrogen make it easier to transfer electrons from the catalyst to the adsorbate, and the adsorption energy changes more rapidly with the applied potential. Regarding the modulation, axial adsorption of O_n_H_m_ species helps to mitigate the relatively strong adsorption strength of intermediates on pyridine‐like FeN_4_C, thus optimizing the adsorption energy and improving the catalytic activity.^[^ ^386^ ^]^

The presence of pentagonal carbon atoms in graphene with enriched conjugated π‐electrons facilitates electron transfer from the active site to the adsorbent. And the presence of adjacent graphene nitrogen further disrupts the electron conjugation and improves the activity of the catalytic sites.^[^ ^387^ ^]^ Graphite nitrogen transfers additional electrons to the Fe center, changing the electron configuration and increasing the filling of partially occupied d orbitals, thus weakening the over‐adsorbed *OH and the over‐expanded σ‐bonds in the Fe center as well as the on‐site magnetic moment in the Fe center, and the corresponding catalytic activity of the catalyst ORR increases.^[^ ^388^, ^389^ ^]^ Graphite nitrogen‐doped carbon undergoes a pronounced distortion of its geometry upon adsorption of O_2_ reactants and *OOH intermediates in the oxygen reduction reaction. Moreover, the free energy of graphitic nitrogen‐C in catalyzing the generation of H_2_O_2_ is lower than that of pyridine and pyrrole nitrogen. Therefore, the carbon adjacent to graphitic nitrogen is the main catalytically active site for 2e^−^ ORR.^[^ ^390^ ^]^

In addition to nitrogen doping, other heteroatoms are effective in modulating the electrochemical properties of CAC materials. Since heteroatoms have electronegativity different from that of carbon (B = 2.04, P = 2.19, S = 2.58, Se = 2.55, Cl = 3.16, Br = 2.96, I = 2.66, and C = 2.55), doping disrupts the conjugated system and induces a charge redistribution that allows easy adsorption of intermediates thus directly affecting the ORR activity. When boron or phosphorus is doped in the carbon structure, the charge distribution is very different from that of nitrogen‐doped carbon due to the low electronegativity of the doped atom generating an electron‐deficient system. As shown in Figure 11d, B doping weakens Fe‐3d and N‐2p interactions away from the Fermi energy level, shifts the lower spin resonance to higher energies, improves π‐electron utilization in the conjugated carbon, and increases catalytic activity.^[^ ^391^ ^]^ The boron‐doped sites have excellent quasi‐capacitance kinetics and high electrochemical oxygen reduction properties. The doping of boron atoms enhances the electrical conductivity and provides active sites for the reaction.^[^ ^392^ ^]^ In two‐electron ORR applications, B doping has a near‐zero overpotential and greatly enhanced kinetics without sacrificing high selectivity.^[^ ^393^ ^]^ The CAC material generated by the reaction of NaBH_4_ and CO_2_ is then doped with boron atoms using a boric acid treatment to activate the carbon substrate pore structure and form more edge defects. And the doping of boron atoms at the edge position gives the catalyst a larger surface area, contributing to the high selectivity and activity of the two‐electron oxygen reduction.^[^ ^394^ ^]^ The introduction of P dopant in Co─N_4_ CAC‐based catalysts, carbon substrate doped with P induced electronic modification as well as coordinated electronic structure, changed the microenvironment such as valence state of Co and Co‐N bond length, reduced the electron density of Co atoms and optimized the key intermediate (*OOH), thus improving the catalytic efficiency.^[^ ^395^ ^]^ During the reaction, the metal species in the carbon matrix react with oxygen as well as the conversion of phosphide to the corresponding phosphate, the synergistic effect of phosphide and phosphate with metal accelerates the oxygen reaction, and the crystalline and amorphous nanostructures in the metal‐carbon based catalysts combine into optimal crystals to ensure excellent stability of the catalyst.^[^ ^396^ ^]^ Doping phosphorus with phosphorylation reduces the crystallinity of the metal, tends to the amorphous state of the metal and metal oxide on a carbon substrate, increases the active sites on the catalyst surface, and increases the catalytic activity.^[^ ^397^ ^]^ S and Se atoms have similar electronegativity to carbon and have less effect on charge distribution but cause a redistribution of spin density or distortion of carbon structure to regulate electrochemical properties. The doping of sulfur produces different electronic regulation mechanisms for different metals. The introduction of sulfur decreases the Bader charge at the Fe site, but has a negligible effect on the Bader charge at the Ni site, but decreases the d‐band center of NiN_4_. The anisotropy of the electronic structure coordinates to change the binding ability of different oxygen‐containing intermediates, thus improving the oxygen catalytic performance as seen in Figure 11e.^[^ ^398^ ^]^ When the carbon material has a large specific surface area and a developed multi‐stage porous structure, it can fully expose the dense and evenly embedded positions of rare atoms on the surface of carbon matrix. S atoms will be embedded in the carbon lattice or anchored at the edge. Adding ZnS to the composites can improve the doping level of S, while MgO can enrich thiophenic‐S groups. Therefore, a high level of S doping and the balanced distribution of thiophenic and oxidized S substances will give the catalyst excellent catalytic activity. Therefore, a layered carbon structure rich in defects can be constructed to adjust the density of S doping.^[^ ^399^ ^]^ The abundant d electrons and high polarizability of selenium atoms increase the content of the low spin‐polarized conformation of Fe in the catalyst. Therefore, the doping of selenium atoms is beneficial to improve the adsorption strength and conductivity of ORR intermediates and to regulate the electronic state and spin structure of the Fe active sites. The lowering of the reaction energy barrier of the decisive step. the lower sublimation temperature of SeO_2_, during the preparation process, volatilizes a large part of it, making the rich pore structure appear on the catalyst.^[^ ^400^, ^401^ ^]^ When the carbon material is built with a multilevel pore structure, the pore confinement effect can be used to stabilize the Fe monoatom due to the high accessibility and utilization within the material. The high content of N introduced into the porous carbon material will become the anchoring point for Fe atoms during the pyrolysis process, and the introduction of Se can further modulate the electronic structure of Fe–N_4_ and increase the spin moment of the Fe center to optimize the adsorption of ORR intermediates and improve the intrinsic activity of the catalyst.^[^ ^402^, ^403^ ^]^ Alternatively, the ball milling technique can be used to construct Fe and Se bis‐monatomic sites, asymmetrically coordinated Fe‐N_5_, and partially present SeC2 bis‐active sites to facilitate the resolution of *OH.^[^ ^400^ ^]^

#### 5.1.4 Multi‐Heteroatom Doping

Different heteroatoms have different electronic configurations and atomic radii, and their properties are used to serve CAC materials with different performance requirements. Therefore, the structural coordination brought by the co‐doping of different heteroatoms makes the co‐doping valuable for research, and the research group hopes to obtain catalysts with more active centers, stronger hydrophilicity, better electronic conductivity, and large specific surface area with balanced micro‐mesopores by co‐doping.

The synergistic coupling effect between double‐doped/multi‐doped atoms in CAC materials to improve the physical/chemical properties becomes an effective strategy, usually with N doping as the main body and other heteroatoms as co‐dopants.

Co‐doping B and N with similar size to carbon will not cause structural changes, but the p‐type doping caused by the intrinsic electron‐deficient nature of B atoms is different from the n‐type doping of electron‐rich N atoms, so the co‐doping of both will cause significant electronic effects and have a synergistic optimization effect on the physical and chemical properties of CAC materials. N‐doping will change the electron distribution of adjacent C atoms, so that the C atoms at the edge of the armchair nanoribbon adjacent to graphite N become ORR active site, pyridine nitrogen lone pair electronically induced charge transfer from π orbitals to O_2_ antibonding orbitals, contributing to electron transfer and reducing the ORR overpotential. The B doping facilitates the adsorption of O and intermediates on the catalyst, and the B atom replaces the *sp* ^3^ C atom to form the BC_3_ structure, and the whole structure redistributes the charge to form the polarization active catalytic site. The synergistic effect of both pyridine N and BC_3_ significantly improves the ORR catalytic activity.^[^ ^404^ ^]^

Nitrogen‐phosphorus co‐doped CAC materials are also a good choice for the preparation of highly efficient ORR catalysts, and phosphoric acid impregnation of nitrogen‐rich CAC materials can be used to prepare nitrogen‐phosphorus co‐doped catalysts efficiently and cost‐effectively. The presence of phosphoric acid as a surface activator promotes the generation of more micropores/mesopores as well as carbon edges and defects in the CAC material, facilitating the transient in situ doping of more nitrogen atoms into the carbon framework during the subsequent pyrolysis process. The final CAC materials obtained by nitrogen and phosphorus co‐doping are characterized by a large specific surface area and abundant mesoporous structure, and these structural features expose more active sites, conducive to mass transport and improved ORR catalytic activity.^[^ ^405^, ^406^ ^]^ The volatile small molecules produced by hydrothermal treatment with phosphoric acid as a solvent during the preparation of CAC materials using biomass material (shrimp shells) as a carbon source improved the pore structure of the carbon matrix, promoting better mass transfer, and the doping of phosphorus formed more crystal defects. The final prepared catalyst has catalytic performance close to 20% Pt/C, providing a new research and development direction for shellfish waste reuse.^[^ ^407^ ^]^ The establishment of metal sites for nitrogen‐phosphorus coordination changes the electronic structure of the microenvironment, facilitating the adsorption/desorption of oxygen intermediates and accelerating the reaction kinetics and catalytic oxygen reduction activity.^[^ ^408^ ^]^ While doping phosphorus atoms into the carbon substrate of the Co–NC catalysts lengthens the Co–N bond, decreases the Co electron density, and weakens the adsorption strength of *OOH intermediates at the active site, finally obtaining catalysts with more than 90% hydrogen peroxide selectivity in a wide potential range (0.1–0.7 V).^[^ ^395^ ^]^

S and Se atoms have the same electronegativity as C atoms, and their own properties of large atomic size and unique electron configuration can widen the carbon layer spacing, change the spin density distribution of the carbon matrix, and create internal defects in the carbon matrix. When co‐doped with N atoms, the N atoms effectively increase the electrical conductivity of the CAC material, and the combination of the two improves the catalyst charge mobility and reactant species accessibility. Co‐pyrolysis of nitrogen source and sulfate can successfully dope nitrogen and sulfur atoms onto the carbon matrix with higher charge density and more significant electron gain and loss in the final electronic configuration. In contrast to N‐doped carbon, N, S doping changes the electronic configuration of the carbon layer to produce more catalytic centers and the electron transfer with the metal adjusts the electron density of the carbon layer in favor of the electron balance of the adsorbed oxygen atoms as shown in Figure 11f.^[^ ^409^ ^]^ The adjacent modified sulfur anion transfers charge to the metal atom, forming electron‐rich states and strong coupling bonds to accelerate the reaction kinetics and improve the conductivity of the catalytic system, and the metal central site with adjacent sulfur anion in neutral media can irreversibly combine with the hydroxyl group to reconstruct the kinetics and effectively regulate the adsorption/desorption state of the reaction intermediate to oxygen reduction, and the whole reaction process has a lower thermodynamic overpotential than the M‐N_4_ catalyst with efficient ORR catalytic activity.^[^ ^410^ ^]^ The nitrogen and sulfur co‐doped porous CAC materials not only show excellent catalytic activity in neutral media but also perform well in acidic and basic media.^[^ ^411^ ^]^ The introduction of S atoms in the M‐N_4_ configuration of the catalyst does not change the geometry, but the charge state of the central metal atom is changed by the combined action of the ligand nitrogen and the outer sulfur atom. In the central metal atom after the change in charge state weakens the adsorption strength of *OOH intermediates, making it easy for *OOH intermediates to be removed and H_2_O_2_ products are formed. Thus the sulfur‐doped M‐N_4_ conformation becomes a catalyst with high two‐electron oxygen reduction activity and hydrogen peroxide selectivity.^[^ ^412^ ^]^ When the selenium doping is reduced to atomic size can be used as an effective ORR active site to modulate the local electron configuration on porous nitrogen‐doped carbon substrates, lowering the reaction energy barrier and optimizing the reaction path, showing excellent ORR activity and stability.^[^ ^401^ ^]^ In the strongly coupled Fe_2_NiSe_4_@Fe‐NC hybrid materials, selenium strongly interacts with nitrogen, forming Se‐GNG bridge bonds to become a bridge for electron transfer from Ni and Fe to graphite‐N, facilitating electrolyte ion transport, electron excitation to pyridine‐N, facilitating O_2_ molecule capture, and enhancing ORR/OER coupling‐interaction and stability.^[^ ^413^ ^]^ When the catalyst active sites are FeSe nanoparticles protected by different morphological nitrogen species and nitrogen doping, the doped N atoms on the carbon skeleton enhance the charge leaving the domain of C atoms and improving the electron utilization, the FeSe heterojunction promotes charge transfer and oxygen dissociation efficiency, and the coupling effect of good multi‐level pore size three‐dimensional skeleton structure and multiple active sites further improves the ORR and OER performance.^[^ ^414^, ^415^ ^]^

Ternary or poly heteroatomic doping is still an unexplored area and less research has been reported than single or diatomic doping. To be clear, ternary doping is more synergistic than single or binary doping, yielding more significant asymmetric charge density and spin density. But maintaining a high concentration of doping sacrifices the degree of graphitization and thus reduces the conductivity of the catalyst, so it becomes necessary to develop simple strategies for multiple doping that can balance the doping degree and conductivity. The high doping concentration, large specific surface, and high graphitization of N, P, and S‐triple‐doped carbon flakes can be synthesized by high‐temperature pyrolysis with thiourea using phosphate groups and nitrogenous bases of DNA itself. Due to the highly ordered arrangement of the groups on the DNA backbone, it is easy to achieve in situ doping of the heteroatoms on the carbon backbone into highly active‐active sites. The prepared catalysts exhibit a half‐wave voltage of O.88 V at alkaline ORR (Pt/C half‐wave potential of 0.86 V), and the charge/discharge stability is also superior to that of Pt/C when applied to rechargeable zinc‐air batteries.^[^ ^416^ ^]^ Similarly, nitrogen, phosphorus, and sulfur triple‐doped porous CAC materials can be prepared by mixed pyrolysis of zinc pyrithione and phytate. 95.2% of nitrogen atoms in the form of graphitic nitrogen and pyridine nitrogen and 92.2% of phosphorus atoms in the form of P–C bonds are present in large quantities to bind to the adjacent carbon, maximizing the electron‐modulating effect and becoming the necessary active site for electrocatalysis. The heteroatoms of different sizes disrupt the lattice geometry, creating folds and edges that produce greater differences in charge and spin density. Meanwhile, the carbon substrate remains highly graphitized after multiple doping to maintain effective charge transfer, and ultimately the catalyst exhibits excellent basic ORR activity, high selectivity, and selectivity.^[^ ^417^ ^]^ The use of secondary nitrogen precursors can affect the number, quality, dispersion, and utilization of active sites in different ways, thus improving the electrocatalytic activity of metal‐free N‐doped carbon. Similarly, the pyrolysis of tertiary nitrogen precursors (i.e., three different nitrogen precursors) can be reasonably designed along the same lines to synthesize triple‐doped porous graphene with nitrogen, fluorine, and sulfur, and the overall synergistic effect of the three elements co‐doping improves the ORR catalytic activity. Among them, the doping of fluorine changes the charge distribution of the graphitic carbon framework, reducing the charge leaving domains and introducing a large number of vacant sites. The presence of vacancies facilitates the doping of N and increases the active sites. The sawtooth and armchair edges of the carbon framework are doped with S atoms to induce electron spin redistribution, and the large size of S atoms introduces more defects in the carbon skeleton. By controlling the nitrogen precursors to influence the surface area and active center conformation of CAC materials, synergistic more active F and S doping improves the catalyst performance with Pt/C equivalent ORR activity.^[^ ^418^ ^]^ When the preparation method was changed from mixed pyrolysis of several different heteroatomic precursors to single precursor pyrolysis, it was good to circumvent the surface functionalization and prepare catalysts to obtain an overall uniform distribution of electrocatalytic active sites, and the heteroatom‐rich covalent triazine polymer (FP‐CTP) became a good choice. Due to the presence of abundant and uniform nitrogen, phosphorus, and fluorine in the cross‐linked structure of the FP‐CTP backbone, these heteroatoms are still well‐doped in the carbon backbone to form effective catalytic active centers after high‐temperature carbonization treatment and the prepared CAC material has great wettability due to the fluorine enrichment, making the electrolyte in close contact with the catalyst layer, and combined with the presence of high porosity in the carbon backbone, it can exhibit The excellent ORR electrocatalytic activity in different pH domains.^[^ ^419^ ^]^

#### 5.1.5 Superdoping

The preparation of catalysts with high doping amounts by simple and controllable methods will break the limitation of the number of active sites, and this is the key to solving the problem that metal‐free heteroatom‐doped carbon is less active than metal catalysts. Therefore, the trend is to obtain uniformly high doping concentration, high reproducibility, and high stability materials. As mentioned earlier, the uniformly distributed vacancies generated by thermal defluorination of fluorinated CAC materials induce high levels of nitrogen doping, thus achieving a wide range of controlled doping levels and heteroatom‐producing doping of CAC materials as seen in Figure 11g. The high concentration of vacancies produced by annealing after fluorination is also suitable for high‐level doping of other heteroatoms, and the heteroatom doping level can be precisely adjusted in a wide range by changing the fluorination degree of the CAC material, but excessive fluorination/doping will produce severely distorted π‐π conjugation, leading to irreversible changes in the physicochemical properties of the CAC material.^[^ ^420^, ^421^ ^]^ The high N concentration of CAC material obtained by fluorination treatment enhances the electrical conductivity and wettability, and combined with the large surface area of CAC material, its electrochemical performance is excellent in acid, alkali, and neutral media with strong stability.^[^ ^422^ ^]^ In addition to the fluorination of CAC materials, the reaction of graphite fluoride with sodium azide can be directly used to complete the defluorination to introduce vacancies and nitrogen super‐doping, and the preparation method is relatively simplified, but the nitrogen doping effect is significant.^[^ ^423^ ^]^

### 5.2 Defect

Theoretically, CAC materials inherently contain defects and it is crucial to explore the influence and contribution of defects in CAC‐based catalysts. The formation of suspension bonds at unsaturated coordination sites in defective locations makes the performance more active, improves the activity of the reaction sites, and optimizes the adsorption energy of the associated reaction intermediate species. Defects in CAC materials fall into two main categories: non‐intrinsic defects caused by doping with heteroatoms or metal atoms, and intrinsic defects due to carbon atom self/rearrangement without any dopant or “foreign matter”. This section will focus on the various intrinsic defects (edge defects, point defects, topological defects). To obtain higher electrocatalytic activity, the formation and preparation of defects in CAC materials, the dynamic evolution of defects during the reaction, and the effect of the dynamic evolution of defects on the catalytic reaction is the basis for the preparation of stable defect‐structured CAC‐based catalysts with high recombination activity. Analysis of the conformational relationships between catalyst structure, electronic structure, and electrocatalytic performance can effectively improve the design of defective catalysts and can guide the design of high‐performance catalysts for practical applications.^[^ ^62^, ^424^, ^425^ ^]^

#### 5.2.1 A Defect Driven Catalysis Mechanism for the Oxygen Reduction Reaction

Heteroatom doping drives the charge redistribution of carbon networks to make active sites to promote chemisorption during ORRs, and in turn, affects the catalytic performance of CAC materials. In contrast, the presence of defects or disordered structures in CAC materials can disrupt the absence or reconfiguration of certain carbon atoms in the lattice, disrupting the electron‐hole symmetry of CAC materials, together with the presence of saturated groups in the defective regions can interfere with the π‐electron local density, thus affecting the in‐plane charge transfer causing charge localization. Thus, it can be seen that defect engineering as a method that can also change the electronic configuration of carbon atoms can promote ORR and is a starting point to investigate the development of effective methods to modulate the catalytic properties of CAC materials.^[^ ^5^, ^79^, ^371^, ^426^ ^]^

**Figure 12:**
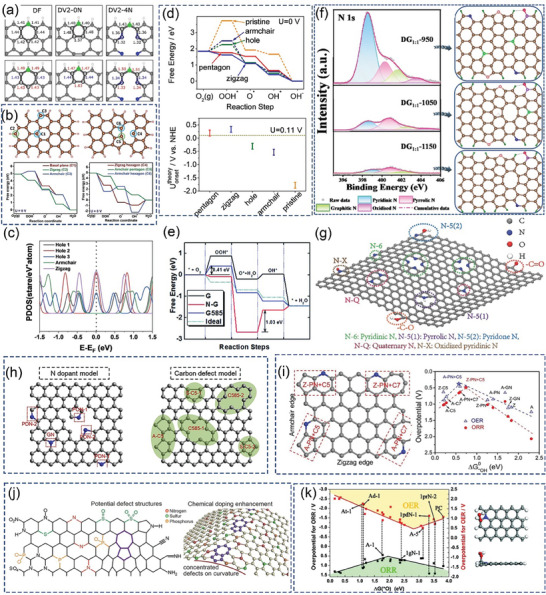
a) Changes in bond lengths after O_2_ adsorption. Bond lengths before (after) O_2_ adsorption are shown in the top (bottom) panels for DF, DV2‐0N, and DV2‐4N. The red and blue numbers represent increased and decreased bond lengths, respectively. The adsorbed O_2_ molecules in the bottom Figures are not shown for clarity. All bond lengths are in angstrom units.^[^ ^427^ ^]^ Copyright 2022, American Chemical Society. b) Theoretical models, and the corresponding free energy diagrams for perfect and defective carbon atoms of graphene cluster at electrode potentials U = 0 in 0.1 M KOH.^[^ ^431^ ^]^ Copyright 2023 Wiley‐VCH GmbH. c) Density of states for armchair, zigzag, hole1, hole 2, and hole 3 defects carbon site and the final adsorption energy of *OOH.^[^ ^435^ ^]^ Copyright 2022 Elsevier B.V. All rights reserved. d) DFT calculations for ORR activities of different defects. Free energy diagrams. U_onset_ theory ranges (bars) and U_onset_ exp of CNC700 (dashed line). The calculations were performed by taking into account the solution effect at 298.15 K, and “g” and “*” indicate the gaseous and chemisorbed state, respectively.^[^ ^436^ ^]^ Copyright 2015 American Chemical Society. e) Calculated free energy diagram of perfect monolayer graphene g, N‐doped graphene (N‐G), graphene with G585 defects (G585), and an ideal catalyst (Ideal) for the ORR at the equilibrium potentials.^[^ ^437^ ^]^ Copyright 2024 Copyright Clearance Center, Inc. All rights reserved. f) the corresponding deconvoluted N 1s spectra and the correlated models representing N‐content and defects in DG_1:1_‐950, 1050, 1150.^[^ ^438^ ^]^ Copyright 2024 Copyright Clearance Center, Inc. All rights reserved. g) Various N‐containing functional groups in graphene.^[^ ^439^ ^]^ Copyright 2017 The Authors. h) The schematic of all N dopant types and defect types for DFT calculations. Gray and blue balls represent C and N atom, respectively^[^ ^440^ ^]^ Copyright 2020 Elsevier Inc. i) A schematic graphene nanoribbon with four typical composite active sites. A: armchair edge, Z: zigzag edge, PN: pyridinic‐N, C5: pentagon carbon ring, C7: heptagon carbon ring. For example, the composite of pyridinic‐N and pentagon defect on the armchair edge is labeled as A‐PN+C5 (left). ORR and OER volcano plots of overpotential versus adsorption energy of *OH (ΔG^0^_*OH_) (right).^[^ ^446^ ^]^ Copyright 2018 The Authors. j) Potential defect structures of the NSP‐doped np‐graphene. The expected chemical doping level enhancement by the geometric requirements to form highly curved graphene.^[^ ^448^ ^]^ Copyright 2016 Wiley‐VCH Verlag GmbH & Co. KGaA, Weinheim. k) The volcano plot for the ORR and OER by plotting the overpotential as a function of ΔG(*O) at various possible active sites. The top and side views of the active site A‐1 for the ORR^[^ ^452^ ^]^ Copyright 2024 Copyright Clearance Center, Inc. All rights reserved.

The presence of defects, in addition to affecting the charge distribution, causes structural deformation, and this change affects the change in bond length and angle around the defect before and after O_2_ adsorption as seen in **Figure** **12**a. the proximity of O_2_ disturbs the *sp* ^2^ hybridized carbon atoms (C_ad_), pulling the carbon atoms up from the carbon plane. in a rigid two‐dimensional carbon structure, it is difficult for the adjacent carbon atoms to move to release the strain present in the original carbon, precisely because of the high energy movement The cost limits the *sp* ^2^/*sp* ^3^ leap of C_ad_ and inhibits the adsorption of O_2_. The introduction of defective CAC materials can release the stress due to the flexible structure and reduce the *sp* ^2^/*sp* ^3^ leap energy barrier of carbon atoms so that the adjacent edge carbon sites can adsorb O_2_.^[^ ^427^ ^]^

#### 5.2.2 Edge, Hole Defects

Pure CAC materials with a large number of intrinsic defects can effectively stimulate the charge redistribution of conjugated *sp* ^2^‐carbon substrates to improve the ORR activity, so that a large number of cavity defects can be created on the surface of the material to expose a large number of jagged and armchair‐shaped edge defects, greatly increasing the spin and charge density of the edge carbon atoms to further improve the ORR performance.^[^ ^428^, ^429^ ^]^ Wang et al. used an electrochemical micro‐manipulator‐microinjection system to test the electrochemical properties of CAC materials at specified locations, and the experimental results visually demonstrate that the off‐domain charge distribution of the edge carbon atoms ensures more active reactivity of CAC materials with more exposed edges.^[^ ^430^ ^]^ By gradually evaporating the zinc‐based metal oxides in the CAC material through high‐temperature pyrolysis leaving a large number of cavities, a large number of edge defects, fracture lattice streaks, and cavity defects appear on the surface and sides of the CAC material. Under the condition that all carbon atoms are saturated with hydrogen atoms (excluding suspension bonds) and based on the first nature principle calculation, the theoretical model of the target carbon center at the edges and holes is established, and the free energy diagram of each carbon site at each step of the ORR process can be analyzed to show that the jagged edge position and armchair carbon atom position at the cavity are free energy drop (exothermic reaction) paths at zero potential, and these two sites are ORR actual active sites as seen in Figure 12b.^[^ ^431^ ^]^

The abundant edge defects obtained during the high‐temperature pyrolytic activation of CAC materials are a major contributor to the two‐electron ORR, and undoped defect‐rich CAC materials with defects as active sites can also be obtained with high selectivity and high activity.^[^ ^432^, ^433^ ^]^ In addition to the removal of doped atoms to prepare defective carbon, it is feasible to use the CO_3_^2−^/CO_2_ electro‐reduction The electrochemical and chemical reactions are synergistically used to achieve CO_2_ recycling by exchanging only two electrodes to reverse the current and etching the deposited carbon into carbon with cavities and edge defects after the defect‐building effect in the oxidation process. The relationship between the defect density and the selectivity of the two‐electron ORR shows an increasing and then decreasing trend, so it is necessary to control the defect density to an optimal value.^[^ ^434^ ^]^ Interestingly, the method can achieve reasonable control of the defect density by controlling the oxidation time and reoxidation current and avoiding the randomness of defect preparation. Based on the 2p orbital partial density of states (PDOS) at different defect sites, it can be seen that the cavity 1 and armchair C‐2p peaks do not appear as antibonding states that are easily filled by *OOH adsorption, and this will enhance the product state energy and reduce chemisorption, while the intensity of the Fermi energy level C‐2p peak of the cavity 3 is between that of cavity 2/zigzag and cavity 1/armchair, indicating moderate adsorption of * OOH. The C‐2p peaks of cavity 2 and zigzag defects are strongly increased, improving the *OOH orbital overlap and being highly active for 2e^−^ ORR as seen in Figure 12c.^[^ ^435^ ^]^

Carbon nanocages with abundant intrinsic defects were synthesized using in situ magnesium oxide template method using benzene as a precursor, and the catalytic activity of this pure carbon catalyst without any dopant was comparable to that of N‐doped carbon. Density generalized theory calculations conclude that in addition to zigzag and armchair edge defects and hexagonal cavity defects, there are also pentagonal defects with positive topological orientation errors. Comparing the free energy diagrams and theoretically predicted onset potentials of different defects as seen in Figure 12d, the ORR performance of armchair and zigzag defects with the same edge defect is not similar, considering that the unpaired π‐electrons of the two adjacent carbon atoms of the armchair defect pair up to form stable covalent bonds, and the zigzag edge carbon atoms have unpaired π‐electrons that facilitate the transfer of electrons to O_2_ to induce the formation of intermediate *OOH, therefore The difference in electronic structure leads to completely different ORR properties for these two different shapes of edge defects. In general, the ORR free energy decreases gradually at the pentagonal and sawtooth defect sites and possesses a more positive onset potential, so the pentagonal and sawtooth defect sites are the main contributors to the high ORR activity of carbon nanocages. Therefore, in addition to edge and cavity defects, it is also worthwhile to study the effect of modulating different topological defects on ORR performance to make it a major contributor to our preparation of highly active electro‐oxygenated CAC‐based catalysts.^[^ ^436^ ^]^

#### 5.2.3 Intrinsic Topological Defects

In addition to edge positions, intrinsic topological defects in the face (non‐hexagonal rings such as heptagonal and pentagonal) can cause local Gaussian curvature, long‐term deformation, and electron orbital rehybridization. The charge of intrinsic topological defects can be effectively modified, thus modulating the activity of the CAC material. By trapping oxygen molecules at the defect sites, oxygen adsorption on the defected CAC material can be enhanced compared to the pristine material. Pure defect CAC‐based catalysts were obtained by removing N‐ dopant and retaining unstable carbon vacancies to form stable defect structures. The effects of defects on ORR performance were studied.^[^ ^380^ ^]^ G585 contains two topological defects (two pentagonal carbon rings and one octagonal carbon ring), and this double‐vacancy structure is more stable than single‐atom vacancies, and the decrease in N content increases the defect density of G585, making G585 an ideal material for probing defects and catalytic properties. Comparing the performance of perfect monolayer graphene g, nitrogen‐doped graphene (N‐G), graphene with G585 defects (G585), and ideal catalyst in the calculated free energy diagram of ORR at equilibrium potential as shown in Figure 12e, it can be seen that G585 is thermodynamically favorable and to be close to the ideal catalyst in all reaction steps, it does not have dangling bonds itself, the O‐species will not be strongly bound to The O‐species are not strongly bound to the CAC material, and the radical reactions are all exothermic, but the N‐doping is not favorable for the chemisorbed oxygen atom (O*) reduction, and its decisive step O*→OH* requires a high energy input of 1.03 eV, demonstrating that G585 has better catalytic performance than the N‐doping.^[^ ^437^ ^]^ As seen in Figure 12f, nitrogen loss due to heat treatment (pyridine nitrogen, graphite nitrogen) is the cause of defects (5‐8‐5 or pentagonal defects) in CAC materials, so the defect concentration required for optimal ORR activity can be obtained by adjusting the annealing temperature.^[^ ^438^ ^]^

The controllable preparation of defects in CAC materials becomes a reality when a specific N configuration (pyridine‐N, graphite‐N, or pyrrole‐N) corresponds to the topological defect configuration (Figure 12g).^[^ ^439^ ^]^ The carbon lattice is disrupted by the introduction of N atoms, and the N‐doped samples are effectively converted into defects after zinc‐induced edge‐engineered hydrothermal treatment, and thermogravimetric analysis yields the release of N atoms in whatever form they are present in the carbon structure without loss of carbon atoms, leaving only new reconfigured defects. The graphite‐N atom is bonded to three neighboring carbon atoms inside the perfect carbon matrix, and the single vacancies created after the removal of the high energy input migrate and merge into highly stable double vacancies (C585). In contrast, the removal of pyridine‐N atoms in the exposed part of the hexagon forms pentagonal defects at the edges (S‐C5), as well as the removal of pyrrolidine‐N atoms located at the apex of the pentagon to form a hexagonal carbon lattice or adjacent pentagonal defects (A‐C5) as seen in Figure 12h. This also implies that a rational design of the starting carbon configuration to introduce a specific N configuration can synthesize the corresponding ideal defect type.^[^ ^440^ ^]^ Tuning the *sp* ^3^/*sp* ^2^ carbon ratio on the basis of tuning to obtain a suitable topological defect conformation can also have a positive effect on improving the catalytic activity of CAC‐based catalysts. When the other two carbon atoms of the pentagonal carbon *sp* ^3^ hybridization, the overall charge density diffuses and a spin‐down vacant orbital appears above the Fermi energy level, enhancing the adsorption of O_2_ molecules, the superpotential of the reaction process drops to 0.48 V. The rate‐determining step (RDS) changes from the original O_2_ activation to that of OH desorption. Thus there is a strong synergistic effect in topological defects (pentagonal carbon) and hybridization states (*sp* ^3^/*sp* ^2^ carbon interface) to improve ORR activity.^[^ ^441^, ^442^ ^]^

In chemically etched Mo_2_C defects in constructed CAC materials, single‐vacancy carbon (SVC), double‐vacancy (DVC), and Stone Wales 5757 carbon (5757C) sites show lower 2e^−^ ORR energy barriers and more moderate *OOH reaction‐free energies than in pristine CAC materials, and single‐vacancy carbon (Edge C‐DVC) in edge positions and removal of the 5757C conformation The new topologically defective structure (5757C‐D) with a moderate Bader charge (4.02‐4.05) after the re‐evolution of the Mo atoms located at the intersection of the pentagonal, hexagonal and heptagonal shapes has a moderate Bader charge (4.02‐4.05), facilitating the adsorption and desorption of the intermediate *OOH and thus the generation of H_2_O_2_.^[^ ^443^ ^]^

### 5.3 Defect and Dopant Co‐Promoted Oxygen Reduction Reaction

The introduction of heteroatoms alters the local charge and spin density of the carbon matrix, while the presence of defects or disordered structures at the edges or surfaces of CAC materials due to the absence of carbon atoms or/and lattice reconfiguration breaks the electron‐hole symmetry also alters the charge and spin density.^[^ ^444^, ^445^ ^]^ Therefore, in order to obtain catalysts with positive or/and higher charge and spin density electronic structures to facilitate the chemisorption of oxygen molecules and oxygen‐related intermediates, the simultaneous introduction of heteroatom doping and defect engineering into CAC‐based catalysts becomes an interesting attempt.^[^ ^62^ ^]^

The preparation of high aspect ratio carbon nanotubes ensures that a large number of edge atoms and defects are exposed, and nitrogen atoms are doped after high‐temperature ammonia oxidation treatment. The comparison of volcanic diagrams of *OH adsorption‐free energy of four sites (Figure 12i) shows that the adsorption‐free energy reaches the optimal value when defects and doping (pentacarbon and pyridine nitrogen in jagged edge) cooperate. The coexistence of the two allows charge redistribution and space bending, resulting in the formation of a critical dipole moment. The dipole moment has a strong binding affinity to the reaction intermediates, which makes the catalyst have extremely high oxygen electrocatalytic activity. The catalyst provides a power density of up to 151mWcm^−2^ and excellent discharge/charge stability of at least 300 cycles in an assembled zinc‐air battery.^[^ ^446^ ^]^ The combination of topological defects with multiple dopant atoms is equally feasible and effective. The incorporation of oxygen groups and/or oxygen dopants in monolayer graphene determines the activation of the N‐doped graphene ORR activity, while the presence of oxidation defects integrates the nitrogen‐doped atomic activity centers and the presence of nitrogen dopants optimizes the electronic structure of the graphene system; none of the three can contribute to the highest ORR activity of the catalyst.^[^ ^447^ ^]^ As shown in Figure 12j, the higher curvature and curvature gradient in the bicontinuous open small‐aperture carbon structure matched with the high density of topological defects induced to accommodate more dopant atoms during the subsequent vapor deposition process. This results in optimal local charge and atomic structure, and chemical doping at high curvature gradients and high density of topological defects contribute to increased electrochemically active sites.^[^ ^448^ ^]^ Sulfur‐rich polyphenylene sulfide (PPS) was used as the precursor to form a hollow structure through a self‐propagating high‐temperature synthesis (SHS) reaction to produce a large number of topological defects, and Co‐N/S‐CNBs were successfully prepared by high‐temperature pyrolysis under NH_3_ atmosphere, with additional topological defects and microporous structures produced during post‐processing. The unique hollow structure and highly graphitized composition of the catalysts ensured the charging/discharging stability of the catalysts for more than 360 h. The synergistic effect of the defects and the Co and N sites ensured excellent ORR catalytic activity (*E*_1/2_ = 0.89 eV) in alkaline electrolytes.^[^ ^449^ ^]^

The promotion of ORR by single electron defect engineering (doping or defects) is limited, but when the synergistic effect of N doping and edge defects improves the ORR activity of CAC materials.^[^ ^405^, ^450^ ^]^ The change of carbon catalyst with pyrolysis temperature changes the ratio of different nitrogen conformations, and the proper loss of unstable N conformation during this process increases the number of C(*sp* ^2^)‐C(*sp* ^2^) structures, and the removal of N from the carbon skeleton produces additional carbon edge defects with increased graphitization. While the theoretical ORR energy barrier at the C4 position of the graphitic valley at the edge of the carbon defect is 0.56 eV, and it is a special kind of active center, and a large number of available edge defects and effective N doping are together for the high ORR catalytic activity.^[^ ^451^ ^]^ The volcano plot of ORR/OER overpotential for various active sites on N‐doped graphene monolayers as well as armchair and sawtooth graphene nanoribbons against δG(* O) (Figure 12k) shows the effect of different N doping types, doping concentrations and the distance of the active site from the cavity edge on the catalytic performance. The left/right side of the *x*‐axis in the figure indicates the strong/weak interaction between the O atom and the substrate, and it is obvious that the carbon atom (A‐1) at the edge of the armchair nanoribbon and adjacent to the graphitic N dopant has the lowest superpotential and is the most effective active site for ORR.^[^ ^452^ ^]^

The combination of defects and doping can also achieve excellent performance when applied to two‐electron oxygen reduction. The high content of nitrogen active centers, high pore capacity, large specific surface area, and the presence of a large number of defects provide efficient oxygen adsorption, rapid mass transfer, and high density of active sites for CAC‐based catalysts, and this explains the high activity and selectivity of the generated H_2_O_2_. The pentagonal defect (Z5) leads to a redistribution of surrounding electrons, contributing to the electron transfer from the carbon matrix to the adsorbed *OOH, and after coupling with the graphitic nitrogen dopant presents a higher degree of distribution and enhanced electron transfer. In contrast, the graphitic nitrogen‐doped pentagonal defect has a significant charge separation at the carbon atom and *OH bond, producing an alternative C‐*OOH bond of relatively low strength, ensuring that the catalyst has high selectivity and high activity in the production of H_2_O_2_ from 2e^−^ ORR.^[^ ^453^ ^]^

### 5.4 Single‐Atom Catalysts

**Figure 13:**
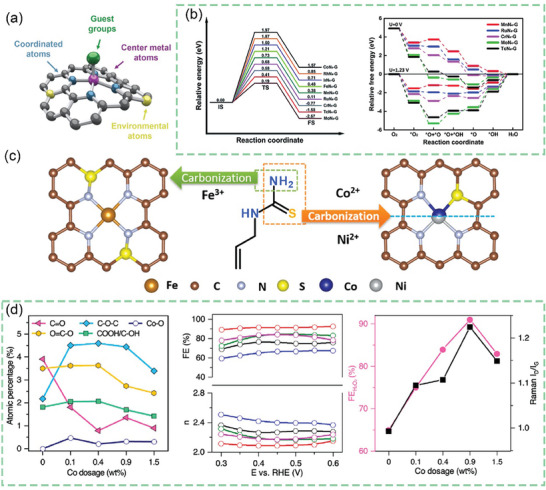
a) Geometric structure of M–N–C SACs, where the typical M–N–C SACs consist of center metal atoms, coordinated atoms, environmental atoms, and guest groups.^[^ ^455^ ^]^ Copyright 2020 Wiley‐VCH GmbH. b) Potential energy surfaces for the minimum energy pathway of O_2_ dissociation on MN_4_‐G. Free‐energy diagram of ORR on MN_4_‐G, and free‐energy diagram of ORR on MN_4_‐G.^[^ ^457^ ^]^ Copyright 2024 Copyright Clearance Center, Inc. All rights reserved. c) The schematic illustration of the formation of Fe‐SAs/NSC, Co‐SAs/NSC, and Ni‐SAs/NSC with different coordination environments.^[^ ^462^ ^]^ Copyright 2019 American Chemical Society. d) The roles of the C−O−C group and carbon defects in ORR catalysis. The corresponding H_2_O_2_ FE and calculated electron transfer number. Atomic percentages of different OFGs on the Co−N/O‐C catalysts with different amounts of Co. Relationships between H_2_O_2_ FE, Co dosage, and *I*_D_/*I*_G_.^[^ ^474^ ^]^ Copyright 2023 American Chemical Society.

Single‐atom catalysts (SACS) have been flourishing due to their high atomic utilization, specific electronic structure, and high specific/mass activity, and combining single‐atom catalysts with carbon‐based supports has become a popular choice to replace noble metal catalysts. In a typical M‐N‐C site, a single transition metal atom is usually coordinated with four atoms in the same plane, and further anchored within the carbon skeleton for uniform distribution. In turn, the presence of the carbon skeleton provides electronic conductivity, large specific surface area, and high pore volume, facilitating mass transfer in the reaction process and exposing more active sites to complement the M‐N‐C sites. The central metal atom interacts directly with the ORR feedstock and intermediates,^[^ ^454^ ^]^ while the ligand atoms share strong electronic interactions in combination with the central atom, and the tuning of both types of atoms can modulate the intrinsic catalyst activity. Carbon skeleton doped heteroatoms can modulate the electronic structure of M‐N‐C sites in long‐range off‐domain, while some small molecules or inorganic particles can interact with ligand‐unsaturated central metal atoms, and likewise affect the intrinsic electrochemical catalytic activity of M‐N‐C through coordination bonds or intermolecular interactions as shown in **Figure** **13**a.^[^ ^455^, ^456^ ^]^

The adsorption and dissociation of O2 molecules during the reaction are strongly dependent on the anchored metal atoms, where CrN_4_‐G, MnN_4_‐G, MoN_4_‐G, and TcN_4_‐G acquire more charge than FeN_4_‐G, CoN_4_‐G, RuN_4_‐G, RhN_4_‐G and IrN_4_‐G with end‐edge adsorption conformation, exhibiting stronger adsorption ability as well as lower dissociation potential. A comparison of the reaction energy, energy barrier, adsorption strength, and free energy of various transition metal monatomic catalysts shows that MnN_4_‐G should be the preferred graphene‐loaded transition SACs as shown in Figure 13b.^[^ ^457^ ^]^ In addition to varying the type of transition metal at the center of the monoatomic catalyst, designing the use of binary monoatomic or ternary monoatomic catalysts is also a strategy to improve the catalytic activity,^[^ ^458^ ^]^ even using a live‐printing method that allows the simple and controlled presence of five or more monometallic atoms uniformly in a single support.^[^ ^459^ ^]^ Depending on the difference in electron density of different metal atoms directs the charge enrichment to achieve higher electron density, allowing the catalyst to obtain the best adsorption/desorption energy for ORR intermediates,^[^ ^460^ ^]^ or the introduction of a third type of metal atom modulates the electronic structure of the two metal active sites to obtain lower d‐band centers, allowing smaller free energy for desorption of *OH as well as a smaller superpotential for the metal active sites, thus enhancing the ORR activity.^[^ ^461^ ^]^

The atomically dispersed metal atoms anchored on the nitrogen‐sulfur co‐doped porous carbon as an effective catalyst possess the active site enrichment as well as effective mass transfer characteristics of single‐atom catalysts as well as porous CAC materials. However, the affinity of sulfur‐containing ligands with different metals leads to different final structures affecting the catalyst ORR performance. Fe has a lower tendency of S coordination in organic ligands and the final S bond is attached to N atoms, while Co and Ni are directly bonded to S as seen in Figure 13c, and after DFT calculations it is concluded that Fe‐SAs/NSC has higher metal‐centered charge density, higher density of states near the Fermi energy level, electron transfer is significantly enhanced and has higher conductivity and catalytic properties, meaning that the coordination state of S atoms plays a crucial role in lowering the reaction energy barrier at the single‐atom center.^[^ ^462^ ^]^ Similarly, the introduction of S‐atoms into the coordination structure applied to the 2e^−^ ORR process also has an impact on performance. When the metal Mo atoms are coordinated to O and S, the reaction intermediates are adsorbed on the carbon atoms near the sulfur, and the electrons are transferred from the Mo atoms to the adsorption sites, while the increase of the coordination number of sulfur enhances the adsorption strength of the reaction intermediates and thus further promotes the 2e^−^ ORR process tunability.^[^ ^463^ ^]^

The effect of active site density, also known as inter‐site distance, on the catalytic reaction should be taken into account when designing the density of single‐atom catalysts on carbon supports. Take FeN_3_ as an example, the Fe‐Fe sites in close proximity transfer electrons to O_2_ and enhance the adsorption activation of O_2_ or OH, so the high density of FeN_3_ will worsen the energy level of ORR's decisive step uphill, but if the neighboring active site adsorbs and activates another O_2_ or OH, it will weaken the binding of OH and moderate the unfavorable energy of ORR's decisive step to the active site, and the neighboring FeN_3_ sites The weak binding of O_2_ and graphene to produce the complex H_2_O_2_ can occur, meaning that FeN_3_ has the feasibility to be used as a 2e^−^ ORR catalyst, but care should be taken to modulate the distance between active sites, as high loading of transition metal sites tends to reduce the production of H_2_O_2_. In contrast, the proximity effect of FeN_4_ is weak, and neither the 4e^−^ nor the 2e^−^ pathway is affected by the Fe‐Fe site distance and the OH or O_2_ coverage on the adjacent FeN_4_ active sites.^[^ ^464^ ^]^

It is also important to design single‐atom catalyst structures with strategies to improve catalyst stability as well as catalytic activity. For example, the protection of the active site is enhanced by using the hydrophilic and hydrophobic ring structure of the cyclodextrin surface to modify the metal center to inhibit the confinement of OH‐ to the central metal atom.^[^ ^465^ ^]^ Alternatively, strategies can be adopted to expose the active site as much as possible, such as using silica‐limited spreading strategies to prepare catalysts with high exposure to single‐atom catalytic sites to achieve low loading and high activity.^[^ ^466^ ^]^ It is also feasible to combine single‐atom catalysts with defect engineering to expand the exposed vacancies and defects in the carbon support to accelerate the transfer of matter and electrons, promote the fastest and fullest possible participation of the active site in the reaction, and optimize the d‐orbital filling and spin state of the transition metal atom by the introduction of defects to increase the ORR activity.^[^ ^467^, ^468^ ^]^ After annealing and regeneration of the deactivated single‐atom catalysts due to the dementalization phenomenon, the internal metal active sites can also recover the ORR catalytic activity upon exposure, meaning that the single‐atom catalysts are regenerative in nature.^[^ ^469^ ^]^

### 5.5 Surface Molecular Functionalization

Surface molecular functionalization is a mild and effective way to induce charge transfer and enhance the anisotropic conductivity of CAC materials than using harsh chemical environments or employing high‐temperature calcination. F4TCNQ (2,3,5,6‐tetrafluoro‐7,7,8,8‐tetracyanoquinodimethane), a p‐type molecule with a low level of lowest unoccupied molecular orbital energy (LUMO), good planarity and strong adsorption of CN groups and F atoms, effectively introduces local electronic imbalance by stacking with graphene without breaking the covalent bond, forming a unique F4TCNQ fixed‐point functionalization that gives graphene a well‐defined interlayer electrical conductivity. Graphene has a clear interlayer charge transfer and electron structure regulation, facilitating the adsorption of reaction intermediates and promoting electrocatalytic oxygen reduction.^[^ ^470^ ^]^ the presence of the P‐n interface facilitates OH ion adsorption, and the non‐oxidized graphene aerogel (NOGA) is modified by p‐doping with adsorbed organic molecules non‐covalent functionalization precisely regulates the surface functional groups and then provides a continuous channel for electrons and reactants to and from the active site.^[^ ^471^ ^]^

The presence of electron‐rich oxygen functional groups (i.e., epoxy groups) can fine‐tune the electronic structure of the metal primary catalytic site, optimizing the excess adsorption energy of the active site on the ORR intermediate, presenting a moderate adsorption strength and improving the ORR activity.^[^ ^472^ ^]^ The zigzag defect positions with high C═O group coverage or other edge configurations similar to the local environment introduced when designing a two‐electron oxygen reduction catalyst become highly active centers that can significantly change the local electronic structure of the active site.^[^ ^473^ ^]^ The catalysts with different Co occupancy vary in N and O concentrations after pyrolysis, and the C─O─C and *I*_D_/*I*_G_ ratios in the oxygen functional groups of this CAC material show a volcano shape, and combined with the corresponding hydrogen peroxide Faraday efficiency (FE) and the performance of the electron transfer number it can be seen that the promotion of C─O─C and defect formation in the graphene layer during pyrolysis indirectly improves the performance of 2e^−^ ORR as seen in Figure 13d, and the addition of Co promotes the graphitization of carbon to produce a higher degree of defects.^[^ ^474^ ^]^

### 5.6 Summary of Strategies and Methods to Improve Catalytic Properties

The researchers did not stop at directly applying electrochemical energy storage and conversion with biomass materials that were only carbonized and activated. The result of simple treatment is that it does not meet the needs of practical applications and is far from exploiting the maximum value of biomass materials. An effective strategy that can change the charge distribution, spin distribution, and energy band structure among carbon atoms is worth developing and adopting. The introduction of heteroatoms can break the charge density symmetry due to different electronegativity and atomic size from carbon atoms, forming localized dense electron‐forming active sites, while changing the overall hydrophilicity of the catalyst. As to how to select the appropriate type and configuration of doping atoms in the catalyst design, it should be correspondingly analyzed whether the changes in adsorption and desorption of reactants/reaction intermediates brought by the introduction of heteroatoms can meet/approach the optimal values calculated by theory, these are directly related to the reactivity and selectivity of the catalyst. To meet the requirements of high‐level and versatile catalyst performance one can use effective strategies such as introducing heteroatoms with different electronegativities and atomic sizes synergistically or preparing catalysts with a large range of uniform high doping amounts. To clarify the doping amount, the spatial distribution of dopant atoms, and the contribution of different dopant atoms to the reaction activity in the co‐doped state is to clarify the conformational relationship between the doping effect of heteroatoms and the catalytic performance, and this also points to the direction for the next step of developing new doping strategies.

The activation of biomass carbonation by carbon atoms themselves/rearrangement gives rise to intrinsic defects, and the presence of defects or disordered structures is also dependent on regulating the charge distribution and thus the catalytic performance by breaking the electron‐hole symmetry of the CAC material. Different types of defects give rise to different electronic structures, and it is important to select defect structures that promote electron transfer to convert O_2_ to intermediate *OOH and proper adsorption of intermediates and can reduce the ORR free energy, such as sawtooth defects (the same applies to the two‐electron pathway), pentagonal hole defects, two‐vacancy G585 topology defects, marginal single‐vacancy C‐DVC defects, and 5757C conformational defects (the latter two defects favor two‐electron oxygen reduction). The precise preparation of the ideal defects and the control of the defect density to the optimal value is the main focus of the current scientific work to overcome. The relationship between doping and defects is not single/absolute; doping generates defects, defects anchor dopant atoms, and when defects coexist with doping they promote the chemisorption of oxygen molecules and oxygen‐related intermediates, but the combination of doping and defects does not play an absolutely positive role, and there are still a few catalysts whose performance is not enhanced as envisioned. As for other methods of inducing charge transfer and enhancing the electrical conductivity of CAC materials (single‐atom catalysts, surface molecular functionalization, etc.) is also possible to adjust the microscopic local structure and site density of single or multiple active sites to adjust the overall catalytic activity of the catalyst. The relationship between dopant/defect and electrochemical activity should be studied clearly and systematically as soon as possible to promote the development of efficient and controllable synthetic catalysts.

## 6 Properties and Application of Biomass‐Derived Catalytically Active Carbon Catalysts

Greenhouse gases such as methane, carbon dioxide, and nitrogen oxides are emitted in large quantities during the combustion of fossil fuels. The discharge of various wastes not only leads to climate change, but also seriously harms the natural environment and human health. Therefore, the overuse of fossil fuels will lead to imminent environmental problems. In addition, the uncertainty of the future cost of fossil fuels makes people begin to reduce their dependence on fossil fuels. Governments began to review their energy strategies and policies to deal with energy and environmental problems.^[^ ^475^ ^]^ Important strategies include improving the current technical efficiency, developing new equipment with high efficiency and little impact on the environment, and partially or completely transitioning to renewable energy.^[^ ^476^ ^]^

Energy and chemical technology are undergoing rapid transformation, and renewable energy is gradually being studied and used to replace fossil fuels. Therefore, the research of renewable energy and the development and application of new energy storage and conversion equipment have become an important topic to reduce the consumption of non‐renewable energy and the negative impact on the environment. Metal–air battery and fuel cell can be used as substitutes for traditional energy because of their environmental protection and high efficiency, which has attracted wide attention in various countries.^[^ ^477^, ^478^ ^]^ Oxygen reduction reaction is the key process in metal–air battery. It is necessary to develop catalysts with high catalytic activity to solve the problem of slow kinetics of oxygen reduction reaction on cathode. It is an important challenge to design and develop low‐cost and sustainable biomass carbon‐based catalysts to promote metal‐air batteries and fuel cells.^[^ ^479^ ^]^ In addition, based on the two‐electron path of oxygen reduction reaction, the development and application of biomass carbon‐based catalyst in two‐electron oxygen reduction reaction to produce hydrogen peroxide is one of the important research directions in a frontier research field with high safety and economic friendliness.^[^ ^480^ ^]^

### 6.1 Metal–Air Batteries

#### 6.1.1 Zinc–Air Batteries

A rechargeable zinc–air battery is a new type of energy storage device that releases or stores energy through a redox reaction between zinc alloy at the negative electrode and oxygen at the positive electrode. Due to the aqueous electrolyte and semi‐open battery design, it has great advantages in terms of energy density, safety, green environment, and cost, and is expected to be a potential candidate for large‐scale energy storage power plants and next‐generation portable energy storage devices.

**Figure 14:**
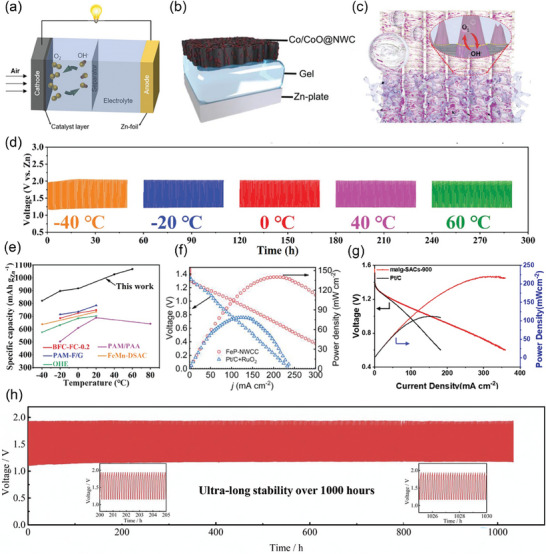
a) Schematic of rechargeable liquid zinc–air battery (ZAB). b) Schematic representation of rechargeable all‐solids. c) Mechanism diagram of triple‐phase boundaries (TPBs).^[^ ^481^ ^]^ Copyright 2021 Wiley‐VCH GmbH. d) long‐time discharge–charge cycling curves of coin cells with C20E2G5 electrolyte and SV‐900 catalyst under different temperatures. e) Specific capacity of coin cells with C20E2G5 electrolyte and previously reported gel electrolytes.^[^ ^483^ ^]^ Copyright 2023 Wiley‐VCH GmbH. f) Discharge polarization curves and corresponding power densities of ZABs assembled with FeP‐NWCC and 20% Pt/C+RuO_2_.^[^ ^485^ ^]^ Copyright 2022 Wiley‐VCH GmbH. g) The polarization curves and the corresponding power density plots for malg‐SACs‐900 catalyst and commercial 20% Pt/C as the cathode catalyst.^[^ ^86^ ^]^ Copyright 2022 Elsevier Ltd. All rights reserved. h) Stability of the charge and discharge cycle (5 mA cm^−2^, 5 min for charge, and 5 min for discharge in each cycle) for the Fe_4_N@N–C‐based ZAB. Inset shows the charge and discharge curves at 200–205 h and 1025–1030 h.^[^ ^89^ ^]^ Copyright 2024 Copyright Clearance Center, Inc. All rights reserved.

The main structural components of the zinc‐air battery include the positive air electrode and the loaded catalyst, the diaphragm, the electrolyte, and the negative zinc electrode as seen in **Figure** **14**a.^[^ ^481^ ^]^ In the discharge process, oxygen in the air diffuses through the air electrode to the catalyst with its ORR, while the oxidation reaction of zinc occurs at the negative zinc electrode, and the electrolyte is conducted by hydroxide ions to form a discharge current in the external circuit. And during the charging process, the positive air electrode catalyst catalyzes the hydroxide to generate oxygen diffusing to the outside of the electrode through the membrane, and the negative zinc electrode plates the zinc ions in the electrolyte to the electrode. The specific electrochemical reactions are as follows:

The total reaction equation during discharge:

(26) $$2\mathrm{Zn}+O_{2}\to 2\mathrm{ZnO},\;E\;=\;1.66\;V\;\mathrm{vs}\;\mathrm{RHE}$$

Electrochemical reactions of electrodes during discharge:

Zinc anode:

(27)

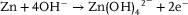



(28)

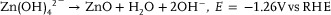




Air cathode:

(29) $$O_{2}+4e^{-}+2H_{2}O\to 4OH^{-},\;E=\;0.4\;V\;\mathrm{vs}\;\mathrm{RHE}$$

The total reaction equation during charging:

(30) $$2\mathrm{ZnO}\to O_{2}+2\mathrm{Zn}$$

Electrochemical reaction of electrodes during charging:

Zinc anode:

(31) $$\mathrm{ZnO}+H_{2}O+2e^{-}\to \mathrm{Zn}+2OH^{-}$$

Air cathode:

(32) $$4OH^{-}\to O_{2}+4e^{-}+2H_{2}O$$

Throughout the process, electrons migrate through the catalyst surface and interface to the active site to undergo a series of oxygen reactions with adsorbed oxygen molecules or OH^−^ reactants, including charge transfer, molecular reconstruction, and bond breaking and generation. The final reaction products desorb off the electrocatalyst surface at the sites. Therefore the active reaction area, conductivity and reaction energy barriers exposed at the catalyst surface interface are critical to the performance of rechargeable zinc air batteries. Fast transport channels for oxygen and ions are provided by strategies such as tuning surface atoms, interfacial stresses, bridge bonds, or electronic structures or constructing nano‐limited reaction interfaces to reduce the concentration polarization phenomenon under high current reactions.^[^ ^64^, ^482^ ^]^ Considering the problems of lack of flexibility, electrolyte leakage, freezing, zinc dendrite growth, and hydrogen precipitation side reactions (HER) on zinc anodes in liquid batteries, the design of solid‐state batteries assembled with gel polymer electrolytes (GPEs) became a good choice as seen in Figure 14b.^[^ ^481^ ^]^ The commonly used polyvinyl alcohol (PVA) gel electrolytes are not freeze‐resistant due to their high water content. Combining Zn^2+^‐treated cellulose to weaken hydrogen bonds and promote dissolution with antifreeze (glycerol) and cross‐linking agents (epichlorohydrin) allows the preparation of solid flexible zinc‐air batteries consisting of excellent ionic conductivity, tensile/compression strength, elasticity, adhesion, and freeze/heat resistance over a −40 °C to +60 °C They exhibit high energy density, high energy density and high HER over a wide temperature range of −40 °C to +60 °C as shown in Figure 14d,e.^[^ ^483^ ^]^

Good progress has been made in the application of CAC derived from biomass to zinc‐air batteries (**Table** **3**). The ordered porous structure of woody biomass is used to construct a three‐dimensional interoperable pore structure to produce a large range of three‐phase interfaces and expose more catalytic active sites. In general, zinc–air battery catalysts use nitrogen‐doped carbon as a support to introduce new active sites to synergistically promote the oxygen‐electric reaction. The nitrogen doping serves as an active site for the adsorption and activation of O_2_ while forming carbon defects in the carbon substrate, increasing the positivity of carbon atoms near the nitrogen and making the carbon atoms moderately adsorbed and rapidly activated with oxygen‐related intermediates, thus improving the ORR activity. Even the nitrogen‐rich porous carbon with a large specific surface area, abundant nanopore structure, and large proportion of pyridine nitrogen prepared from microalgal biomass alone as carbon and nitrogen sources exhibited better peak power density, specific capacity, and stability than Pt/C catalysts in acid and alkaline electrolytes as air cathodes for Zn–Air batteries.^[^ ^484^ ^]^

**Table 3:** Summary of catalytic performance of biomass‐derived CAC‐based catalysts based on four‐electron ORR applied to metal‐air batteries.

Carbon source	Catalyst	Application	Open‐circuit voltage	Power density	Stability	Specific capacity	Reference
Legume root nodules	RN350‐Z(1‐2)‐1000	Liquid Zn–air battery	1.474 V	204 mW cm^−2^	5 days	–	[84]
Starch	Fe_4_N@N–C‐	Liquid Zn–air battery	1.493 V	182 mW cm^−2^	1000 h	768 mA h g^−1^	[89]
		All‐solid‐state Zn–air battery	1.503 V	121 mW cm^−2^	6.6 h	–	
Microalgae	malg‐SACs‐900	Zn–air battery	–	220.7 mW cm^−2^	60 h	546.5 mA h^−1^	[86]
Microalgae	N/biochar‐800–7	Zn–air battery	–	125 mW cm^−2^	60 h	703 mA h/g‐Zn	[81]
Spirulina	Fe_SA_/FeO_NC_/NSC	Zn–air battery	1.44 V	179.0 mW cm^−2^	15 h	641.9 mA h g^−1^	[88]
Chitosan	NC‐5‐900	Al–air battery	1.85 V	2453.4 Wh kg^−1^	–	2144 mAh g^−1^	[83]
Corn silk	Fe SA/NCZ	Flexible Al–air battery	1.441 V	101 Mw cm^−2^	44 h	–	[503]
Phytic acid	P‐CD/G	Al–air battery	1.56 V	157.3 mW cm^−2^	–	–	[500]
Soybean roots	Co_3_Fe_7_‐Fe_3_C/HNC	Al–air battery	1.58 V	210 mW cm^−2^	70 h	–	[100]
Lotus root	NCA_LR_/Fe	Al–air battery	1.81 V	181.1 mW cm^−2^	14 h	–	[502]
Poplar inflorescence	N‐PIACs	Li−O_2_ batteries	–	–	220 cycles	12060 mAh/g	[504]
Citrus maxima peel	CMPACs	Li−O_2_ batteries	–	–	466 cycles	7800 mA h/g	[495]
Oak	ODC	Li−O_2_ batteries	–	–	800 h	12000 mAh g−1	[505]
Basswood	CA‐wood/Ru	Li−O_2_ batteries	–	–	100 cycles	5763 mA h g^−1^	[496]
Miscanthus × giganteus	MGACs	Li−O_2_ batteries	–	–	601 cycles	9400 mAh/g	[492]

The other active site is not the same. The presence of uniformly distributed abundant metallic iron phosphide nanoparticles accelerates water dissociation to generate protons to solve the shortage of ORR protons in alkaline electrolytes, optimizes the adsorption and desorption of reactants and products at the active site by adjusting the electron distribution through the interaction of metallic iron particles and carbon matrix supports, effectively reduces the reaction barriers and promotes ORR (liquid ZABs exhibit a peak power of 144 mW cm^−2^) as seen in Figure 14f and the alloying effect of metallic iron and phosphorus atoms acts to provide excellent durability and stability (over 1350 cycles).^[^ ^485^ ^]^ The same nitrogen‐doped carbon‐enhanced oxygen molecule adsorption and activation accelerates the electron coupling process in concert with the homogeneous distribution of FeNi alloy nanoparticles to activate and dissociate water molecules, accelerating the proton generation and coupling charge transfer processes allowing high‐speed electrocatalytic reactions at the three‐phase interface during the ORR, exhibiting desirable electrochemical properties in ZABs.^[^ ^486^ ^]^ Moreover, the carbon source was changed from wood to peanut shell to synthesize FeNi alloy nitrogen‐doped carbon catalyst, and the charge‐discharge polarization curve and peak power density of the zinc‐air battery were still better than that of 20% Pt/C+IrO_2_‐based battery.^[^ ^487^ ^]^

The same surface phosphorus‐induced CoO particles produce a large number of defects and the altered coordination environment around the metal species can be effectively activated against water and oxygen molecules in the electrolyte, and the partial metal defects that would be produced during phosphorus induction provide excellent stability.^[^ ^488^ ^]^ The constant current electric field induction strategy is used to dope cobalt–nitrogen elements into the monolithic wood‐derived carbon, combing the strong hydrophilicity of N‐doped carbon and the strong hydrophobicity of Co/CoO nanoparticles to form a three‐phase interface of great extent as shown in Figure 14c, providing sufficient space and reaction sites for the gas and electrolyte, and the synergistic effect of both ensures the adsorption of oxygen, dissociation of water and desorption of OH, and the porous carbon structure gives the cathode a good conductivity and fast mass transfer channels in liquid ZABs with a power density up to 152.8 mW cm^−2^.^[^ ^481^ ^]^ The electronic interoperability strategy of transition metal nitride and biomass carbon (from cattail) is adopted to anchor Co and N elements forming Co_2_N configuration to the carbon matrix leading to the increase of electronic states at the near‐Fermi energy level, and the higher support density facilitates the adsorption and charge transfer to the active center during the electrocatalytic process, and Co_2_N and nitrogen‐doped carbon synergistically ensure the interconnected structure of the electrocatalyst to achieve excellent catalytic performance and The synergy of Co_2_N and nitrogen‐doped carbon ensures the interconnected structure of the electrocatalysts, achieving excellent catalytic performance and durability.^[^ ^489^ ^]^

The application of single‐atom catalysts to zinc‐air batteries is a commonly used strategy to improve performance. Efficient and green synthetic Fe single‐atom catalysts were prepared using microalgae cells with endogenous metals introduced to exogenous nitrogen anchored to the carbon skeleton via hydrothermal and pyrolytic treatments. As shown in Figure 14g, the maximum power density of the Zn–Air cell assembled with this catalyst is 220.7 mW cm^−2^, much higher than the maximum power density of commercial Pt/C (112.1 mW cm^−2^).^[^ ^86^ ^]^ It is worth discussing that another form of iron and nitrogen co‐doping exists in the Fe–N_x_ site, where the strong bonding between N and Fe atoms makes this site an efficient and highly stable ORR active site. The resulting Fe_4_N prepared using zinc‐assisted pyrolytic starch exhibited an impressive peak power density of 182 mW cm^−2^ and a stable long‐term cycle of 1033 h in aqueous ZAB as seen in Figure 14h.^[^ ^89^ ^]^

#### 6.1.2 Lithium–Air Batteries

**Figure 15:**
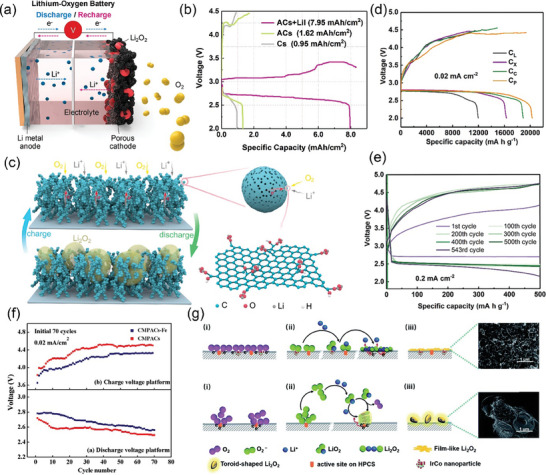
a) Schematic configuration of a Li–O_2_ battery.^[^ ^490^ ^]^ Copyright 2020, American Chemical Society. b) The initial discharge−charge voltage profiles of Li‖1 M LiTFSI/TEGDME‖Cs–O_2_ cell, Li‖1 M LiTFSI/TEGDME‖ACs‐O_2_ cell and Li‖1 M LiTFSI/TEGDME + 0.5 M LiI‖ACs‐O_2_ cell at a current density of 0.02 mA/cm^2^.^[^ ^493^ ^]^ Copyright 2020 Elsevier B.V. All rights reserved. c) Schematic of the cathode based on carbon nanosphere clusters in working Li–O_2_ batteries. d) Initial full discharge‐charge voltage profiles of C_L_, C_X_, C_C_, and C_P_ cathode at a current density of 0.02 mA cm^−2^. e) Voltage profile of the C_P_ cathode cycled at 0.2 mA cm^−2^ current density with controlled discharge‐charge capacity depths of 500 mA h g^−1^.^[^ ^494^ ^]^ Copyright 2021 Elsevier Ltd. All rights reserved. f) (upper part) Discharge and (bottom part) charge platforms of initial 70 cycles of CMPACs and CMPACs‐Fe based Li–O_2_ battery at 0.02 mA cm^−2^ when limiting the discharge capacity to 500 mA h g^−1^.^[^ ^495^ ^]^ Copyright 2018, American Chemical Society. g) Proposed mechanisms for the nucleation of Li_2_O_2_ with different morphologies on the (upper) IrCo@HPCS electrode and (bottom) HPCS electrode.^[^ ^497^ ^]^ Copyright 2024 Copyright Clearance Center, Inc. All rights reserved.

Lithium–air batteries are similar in structure to zinc‐air batteries, consisting of a lithium metal negative electrode, an organic electrolyte containing lithium ions (Li^+^), and a porous catalytic air positive electrode (O_2_ or CO_2_ as the active component) as seen in **Figure** **15**a, so lithium–air batteries consist of a Li–O_2_ battery and a Li–CO_2_ battery, while this section will focus on the Li–O_2_ battery. Lithium–O_2_ battery has a very high theoretical energy density (3500 W h kg^−1^), comparable to gasoline, and is an excellent economic green electrochemical energy storage and conversion device.^[^ ^490^ ^]^

The reaction principle of Li–O_2_ battery during discharge is as follows:

Anode reaction:

(33) $$\mathrm{Li}\to Li^{+}+e^{-}$$

Cathode reaction:

(34)

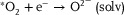

(35) $$Li^{+}\left(\mathrm{solv}\right)+{O^{2}}^{-}\to \mathrm{Li}O_{2}$$

(36) $$2\mathrm{Li}O_{2}\to Li_{2}O_{2}\left(s\right)+O_{2}$$

(37)

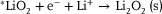




While charging, the highly insulating Li_2_O_2_ is gradually decomposed into Li+ and O_2_ mainly on the not‐sufficient Li_2_O_2_‐electrode solid‐solid contact surface. due to the strong oxidizing unstable intermediates corrosive decomposition of the electrode surface during the reaction, and the side reaction products also gradually accumulate on the electrode surface causing the active sites to be covered, so the reasonable design and development of the catalyst is crucial.^[^ ^491^ ^]^ The porous structure of the catalyst is significant for the performance of Li–O_2_ batteries. The presence of microporous structures accelerates the full transport of electrolyte and O_2_ within the bulk phase, and the defective state microporous structure accompanied by oxygen‐rich functional groups limits the agglomerative growth of Li_2_O_2_ molecules, reduces the charging voltage, and improves the energy efficiency and the reversibility of the electrode process. Biomass‐derived CAC shows great potential in the field of lithium‐air batteries due to its low cost, high conductivity, large specific surface area, and structural diversity.^[^ ^492^ ^]^ Biomass‐derived CAC has also made good progress in the field of lithium batteries (Table 3).

The biomass (skimmed cotton) was prepared by activator‐assisted pyrolysis to obtain a three‐dimensional multistage pore structure with a high specific surface area and appropriate porosity, and the maximum pore volume obtained reduced O_2_ transport losses. Combined with the introduction of lithium iodide (LiI) redox medium to obtain a low voltage gap of 200mV, the low charging potential alleviates most of the side reactions above 3V to produce a relatively stable battery environment. in exchange for a larger area‐specific capacity (7.95 mA h cm^−2^) as shown in Figure 15b.^[^ ^493^ ^]^ The loosely open and interconnected graded non‐rigid slit‐shaped pore structure has the largest active reaction area and can accommodate a large amount of discharge material, ensuring the rapid diffusion of oxygen and Li^+^ and not easily blocked completely by Li_2_O_2_ to realize the high electron conduction rate of the carbon cathode, and likewise promoting the rapid dissolution of O_2_ and Li^+^ from Li_2_O_2_ decomposition during the charging process, resulting in excellent reversibility of the cathode reaction as seen in Figure 15c. The resulting material provides a high discharge capacity of 20300 mA h g^−1^ at a current density of 0.02 mA cm^−2^ and a long cycle life of 543 cycles at a controlled discharge‐charge capacity depth of 500 mA h g^−1^ at a current density of 0.2 mA cm^−2^ in Li–O_2_ batteries as seen in Figure 15d,e.^[^ ^494^ ^]^

When metal active sites are introduced into the catalyst it increases the defect density on the carbon surface, achieving maximum carbon interaction with Li_2_O_2_ and high interfacial boundaries for electron and mass transfer. In comparing the performance of the catalyst charge and discharge cycles before and after Fe doping it can be seen that the Fe‐based oxygen electrode provides a higher discharge plateau and a lower charging plateau in 70 cycles as shown in Figure 15f, with the Fe species acting as a high active site and having excellent conductivity, reducing the discharge and charging overpotentials.^[^ ^495^ ^]^ The controlled Ru loading of 9.3 wt% is uniformly fixed in a 3D hierarchical porous structure of open and elongated microchannels, accelerating oxygen diffusion through the entire electrode while the pore walls are completely wetted to form a thin electrolyte layer for fast Li‐ion transport. This “breathable” wooden cathode increases the area capacity to 56mA h cm^−2^ when using an additional thickness of 3.4 mm.^[^ ^496^ ^]^ IrCo nanoparticles anchored in 3D interconnected porous structures modulate the surface charge more uniformly adsorbed O_2_ reacts with Li^+^ in the electrolyte and transforms the reaction product Li_2_O_2_ from ring‐shaped particles to a uniform film in close contact with the motor as seen in Figure 15g, facilitating charge transfer and effective decomposition at low charge overpotential, and its round‐trip efficiency and cycle life are significantly improved.^[^ ^497^ ^]^

#### 6.1.3 Aluminum–Air Batteries

**Figure 16:**
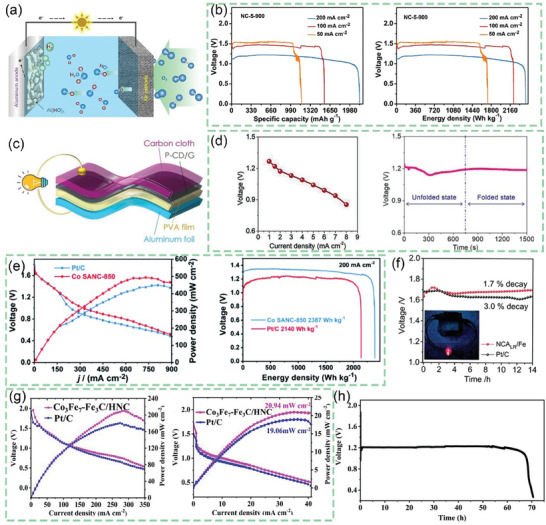
a) Schematic diagram of Al–air batteries consisting of a metal aluminum anode, air cathode (normally porous carbon with catalyst), hydrophobic separator, and electrolyte.^[^ ^498^ ^]^ Copyright 2024 Informa UK Limited. b) specific capacities, energy densities at different current densities for Al–air battery.^[^ ^83^ ^]^ Copyright 2019 Science Press and Dalian Institute of Chemical Physics, Chinese Academy of Sciences. Published by Elsevier B.V. and Science Press. All rights reserved. c) Schematic showing the structure of the all‐solid‐state flexible Al–air battery. d) Plot of discharge voltages versus current density and discharge curve (at different working states) of the all‐solid‐state Al–air battery.^[^ ^500^ ^]^ Copyright 2019 Wiley‐VCH Verlag GmbH & Co. KGaA, Weinheim. e) Polarization and power density plots, energy densities under 200 mA cm^−2^ of Al–air battery.^[^ ^501^ ^]^ Copyright 2024 Copyright Clearance Center, Inc. All rights reserved. f) Constant current discharge tests at the current density of 20 mA cm^−2^; inset: photo of parallel red, yellow, and green LEDs (rated voltages of 1.8 V to 2.0 V) simultaneously powered by only one Al–air battery assembled using NCALR/Fe as the cathode catalyst.^[^ ^502^ ^]^ Copyright 2024 Copyright Clearance Center, Inc. All rights reserved. g) Discharge polarization curve and the corresponding power plots of the alkaline Al‐air batteries using Co_3_Fe_7_‐Fe_3_C/HNC and 20% Pt/C catalysts. Discharge polarization curve and the corresponding power plots under neutral solution. h) Mechanical charge and discharge tests using the Co_3_Fe_7_‐Fe_3_C/HNC at 100 mA cm^−2^.^[^ ^100^ ^]^ Copyright 2020 Elsevier B.V. All rights reserved.

Aluminum metal mineral resources are abundant, inexpensive, and environmentally friendly, with high recyclability. With a theoretical voltage of 2.7V, an energy density of 8.1kW h kg^−1^, and a theoretical specific capacity of 2.98A h g^−1^, aluminum‐air batteries are second only to lithium‐air batteries, but the stable safety of aluminum‐air battery operation is much higher than that of lithium‐ion batteries. Aluminum air battery consists of an aluminum anode, electrolyte, and air cathode as shown in **Figure** **16**a, where the air cathode consists of a conductive collector, gas diffusion layer, and catalyst layer, and the multi‐catalytic layer consists of an electrocatalyst, conductive material, and binder. The electrochemical reactions on the electrodes are as follows:

Anodic reaction:

(38) $$\mathrm{Al}\to Al^{3+}+3e^{-}$$

Cathodic reaction:

(39) $$O_{2}+2H_{2}O+4e^{-}\to 4OH^{-}$$

Total reaction:

(40) $$4\mathrm{Al}+3O_{2}+6H_{2}O\to 4\mathrm{Al}{\left(\mathrm{OH}\right)}_{3}$$

The kinetics of the ORR at the cathode of aluminum‐air batteries is sluggish and prone to electrode polarization and high overpotential, resulting in lower actual discharge power and energy density of aluminum–air batteries, thus requiring the development of new high‐efficiency catalysts.^[^ ^498^, ^499^ ^]^ Biomass‐derived CAC has made good progress in the application of aluminum–air battery (Table 3).

Poly(chitosan) was used as the carbon source to build a multi‐layered porous carbon structure with the help of a gas‐foaming strategy to improve the diffusion of oxygen/electrolyte. The introduction of a large number of edge defects and appropriate nitrogen doping lowered the reaction energy potential barrier, enhanced the adsorption of oxygen, improved electron exchange, and accelerated the diffusion of oxygen reduction active substances. Applying the catalyst to the Al‐Air cell at a high current density (200 mA cm^−2^) alleviated the self‐corrosive hydrogen precipitation reaction at the negative electrode of the Al‐Air cell and improved the specific capacity and energy density, as shown in Figure 16b, resulting in a specific capacity of 900 mA h g^−1^ and energy density of 2453.4 W h Kg^−1^. This is better than Pt/C (1794 mA h g^−1^ and 2140 W h Kg^−1^).^[^ ^83^ ^]^ High P‐doping and homogeneous carbon dot coverage on graphene aerogel by phytate hydrothermal method successfully created many active sites for ORR. And the catalyst was coated on carbon cloth as the air cathode, PVA‐KOH gel as the electrolyte, and aluminum foil as the anode to assemble a foldable all‐solid‐state aluminum air cell as seen in Figure 16c. The cell performed well at various current densities and changing the cell morphology (open or folded) maintained an overall constant voltage (≈1.17 V) at 2 mA cm^−2^ as seen in Figure 16d, and the performance was comparable to Pt/C in a liquid aluminum‐air cell.^[^ ^500^ ^]^ Whether the poly(chitosan) is converted into ultrathin 3D nitrogen‐doped porous carbon using a gas foaming strategy or starch into 3D porous carbon aerogel using pyrolytic acid etching, the microporous defects present on the carbon framework anchor the metal monoatomic well, and the uniformly dispersed Co monoatomic active sites enable fast mass/electron transfer at the electrochemical reaction interface, with excellent high current density at 200 mA cm^−2^ power density, energy density and discharge voltage at high current densities of 200 mA cm^−2^ as seen in Figure 16e.^[^ ^501^ ^]^ The presence of Fe single atoms allows the catalyst to exhibit significant activity and durability, and the assembled Al–Air cell can light three parallel LEDs rated from 1.8 to 2 V with only 1.7% voltage decay at a constant current density of 20 mA cm^−2^ for 14 h. This is better than the performance of the Pt/C cell (3.0% voltage decay), as shown in Figure 16f.^[^ ^502^ ^]^ Considering the high activity and good stability of bimetallic‐based materials, the construction of Co_3_Fe_7_–Fe_3_C heterostructures on biomass‐derived three‐dimensional honeycomb nitrogen‐doped carbon is a good choice, and the presence of heterostructures promotes electron transfer and enhances the intrinsic electrocatalytic activity. The aluminum‐air cell assembled from this catalyst outperformed the Pt/C catalyst in both alkaline and environmentally friendly neutral electrolytes in terms of maximum power density as seen in Figure 16g and sustained discharge at a high current density of 100 mA cm^−2^ for 70 h by replacing the new zinc electrode to exclude the negative effect of anode oxidation depletion as seen in Figure 16h.^[^ ^100^ ^]^

### 6.2 Fuel Cells

A fuel cell is a device that converts the chemical energy stored in the fuel and oxidizer directly into electrical energy and consists of an anode chamber, a cathode chamber, an electrode and electrolyte diaphragm, and an external circuit. The anode chamber is where the oxidation reaction of the fuel (e.g., hydrogen, methanol, etc.) occurs, and the cathode chamber is where the reduction reaction of the oxidizer (air or oxygen) occurs. Polymer electrolyte membrane fuel cell (PEFC) uses solid polymer material as the electrolyte and operates at 50–100 °C, solving the problems of high temperature, easy corrosion, and high pollution of conventional fuel cells. PEFCs are divided into two categories according to the type of internal conduction ions: one conduction of hydrogen protons called proton membrane fuel cell (PEMFC) and one conduction of hydroxide ions called anion exchange membrane fuel cell (AEMFC).^[^ ^506^, ^507^ ^]^ The microbial fuel cell (MFC), combining fully the dual catalytic ability of microorganisms and electrochemistry, can convert the organic oxidative decomposition of industrial and domestic wastewater into electrical energy, and is a highly representative bio electrochemical system. Considering its excellent dual efficacy of energy recovery and wastewater treatment, the application of ORR catalyst in MFC will also be discussed in this chapter.

#### 6.2.1 Polymer Electrolyte Membrane Fuel Cells

**Figure 17:**
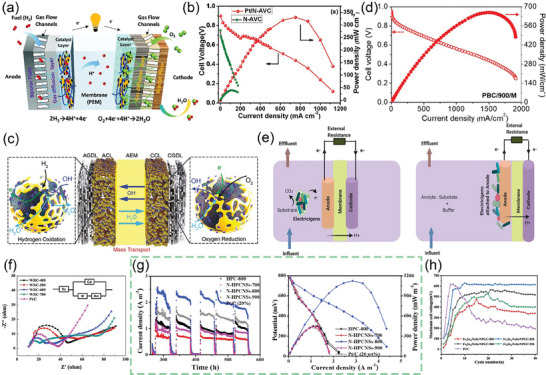
a) Schematic diagram of the working principle of PEMFCs.^[^ ^508^ ^]^ Copyright 2024 Copyright Clearance Center, Inc. All rights reserved. b) PEMFC polarization and power density data for N‐AVC and Pt/N‐AVC catalyst with 4 cm^2^ active area at 70 °C under ambient pressure^[^ ^510^ ^]^ Copyright 2020 Hydrogen Energy Publications LLC. Published by Elsevier Ltd. All rights reserved. c) Schematic diagram of the working principle of AEMFCs.^[^ ^511^ ^]^ Copyright 2021 The Authors. Advanced Science published by Wiley‐VCH GmbH. d) Single‐cell polarization result obtained using PBC/900/M as the cathode catalyst with a 4 mg cm^−2^ loading at 1 bar pressure. The anode was 0.6 mg cm^−2^ PtRu/C, and a 60 wt % ionomer was used.^[^ ^515^ ^]^ Copyright 2021 American Chemical Society. e) Dual‐chambered microbial fuel cell has distinguished cathodic and anodic chambers, single‐chambered microbial fuel cell has a simpler configuration with common chamber.^[^ ^517^ ^]^ Copyright 2021, The Author(s), under exclusive licence to Springer Nature Switzerland AG part of Springer Nature. f) Nyquist plot of cathodes;^[^ ^521^ ^]^ Copyright 2019 Elsevier Ltd. All rights reserved. g) the output current density with a Rex loading of 10 Ω in N‐HPCNSs‐MFC and Pt/C‐MFC; the polarization and power density curves in N‐HPCNSs‐MFC and Pt/C‐MFC.^[^ ^105^ ^]^ Copyright 2018 Elsevier Ltd. All rights reserved. h) The maximum output voltage per cycle during the operation of AC‐MFCs with the five cathodes^[^ ^414^ ^]^ Copyright 2018 Elsevier B.V. All rights reserved.

Proton exchange membrane fuel cells work as shown in **Figure** **17**a. The fuel hydrogen is oxidized at the anode to produce electrons and protons, and transferred to the cathode through an external circuit and a proton exchange membrane (H_2_→2H^+^+2e^−^), while the oxygen reacts with the protons and electrons at the cathode to produce water (1/2O_2_+2H^+^+2e^−^→H_2_O). But, the biggest performance‐limiting kinetic problem of this cell is the slow reaction rate of ORR in the cathode chamber, and the design and development of efficient, inexpensive, and green oxygen reduction catalytic materials have become a top priority.^[^ ^508^, ^509^ ^]^

Using biomass as a sustainable carbon source to construct a three‐dimensional multilevel pore structure to expose the active sites and doping heteroatoms to change the electron cloud distribution to improve the ORR catalytic activity is a basic idea to prepare metal‐free CAC‐based catalysts. The low eutectic NaCl/ZnCl_2_ melt was used as a double template acting on renewable lignin to obtain the ideal three‐dimensional mesoporous skeletal structure of carbon. The combination of NaCl as a confining agent to increase the nitrogen content and ZnCl_2_ as a pore‐forming agent to form rich microporous and mesoporous structures resulted in a carbon matrix with a final three‐dimensional self‐supporting monolithic porous carbon with a large specific surface area and pore volume, leading to easier access to active sites for the reactants and ensuring rapid electron/oxygen transport during the reaction. The doping of chlorine in the carbon matrix polarizes the adjacent carbon atoms and changes the charge/defect distribution on the carbon surface, modulates the intermediate adsorption energy in synergy with the nitrogen atoms, generates the proper theoretical ORR onset potential, and ensures the excellent activity and stability of the catalyst in acidic media. The biochar material was assembled as a cathode catalyst in a proton exchange membrane fuel cell to achieve a maximum power density of 779 mW cm^−2^.^[^ ^108^ ^]^

It can be seen that using biomass‐derived CAC for proton exchange membrane fuel cell applications has some potential, but there is still a big gap between relying solely on non‐metallic catalysts to replace precious metal catalysts, and the addition of metals becomes a reliable attempt to improve the ORR activity of catalysts. The nitrogen‐doped carbon prepared with aloe vera as the carbon source makes the carbon functionalization easy to anchor Pt nanoparticles improves the dispersion and interaction between Pt nanoparticles and the support, enhances the conductivity, charge transfer, and improves the durability and electrocatalytic activity of the catalyst through Pt–N–C interactions. Comparing the catalysts with and without Pt nanoparticles in PEMFC polarization and power density data as shown in Figure 17b can be seen that the addition of Pt obtains a high open circuit voltage (0.93 V) and improves the peak power density to 8 times that of the metal‐free catalyst (330 mW cm^−2^), and can be counted as a remarkable performance in PEMFC.^[^ ^510^ ^]^ Although the performance of noble metal CAC‐based catalysts has been greatly improved, the development of transition metal‐heteroatom‐doped carbon catalysts is promising considering the scarcity of precious metal resources and high prices. The main components of nitrogen‐fixing enzyme systems in naturally occurring legume root nodules contain N, S, and small amounts of Fe and Mo atoms, and the uniform distribution of these elements produces atomically dispersed metal sites. The excellent electrocatalytic performance of the ORR exhibited by the high‐temperature pyrolysis chemical activation of this biomass in 0.1 m HClO_4_ and 0.1 m KOH solutions with half‐wave potentials (*E*_1/2_) of 0.723 V and 0.868 V (vs RHE), respectively, implies the possibility of practical application to proton exchange membrane fuel cells.^[^ ^84^ ^]^

#### 6.2.2 Anion Exchange Membrane Fuel Cells

Anion‐exchange membrane fuel cells (AEMFCs) are gaining attention and rapid development because of their faster kinetics of cathodic ORR in alkaline and acidic environments compared to PEMFCs. AEMFCs are typically composed of a membrane, catalytic layer, gas diffusion layer, graphite plate, sealing material, and other components as shown in Figure 17c. Unlike PEMFC, the AEMFC interlayer uses an anionic membrane that conducts only OH‐ ions and in a different direction than a proton exchange membrane. Oxygen gets electrons and reacts with water to form OH^−^ ions (1/2O_2_+H_2_O+2e^−^→2OH^−^), OH‐ ions pass through the membrane to the anode and react with hydrogen to form water (H_2_+2OH^−^→2H_2_O+2e^−^), and hydrogen loses electrons through the external circuit to form a closed loop. The membrane and the catalytic layer on both sides of the membrane, called membrane electrode (MEA), are the core components of the fuel cell and play a decisive role in the performance and lifetime of the whole cell, and the performance of the membrane electrode is highly related to the catalyst, so the development of highly active and stable oxygen reduction electrocatalysts has become the focus of attention of many researchers.^[^ ^511^, ^512^, ^513^ ^]^

The water vapor activation and thiourea modification of bamboo to obtain S and N co‐doped multi‐stage porous CAC materials exhibited a half‐wave potential of 0.85 V, with similar catalytic activity as Pt/C catalysts, and showed high cell performance of 217 mW cm^−2^ in AENFC, representing the feasibility of biomass‐based catalysts for practical AEMFC applications.^[^ ^514^ ^]^ Co_2_P nanoparticle‐loaded nitrogen‐doped carbon catalysts were prepared from a biomass‐derived CAC source (bean sprouts) following the idea of controlled pyrolysis of metal precursors on biomaterials to induce heteroatom‐doped carbon‐loaded metal nanoparticles. The catalyst showed excellent durability (only an 11.6% decrease in current density after 20 h of chrono‐current measurement) and was much better than the commercial Pt/C catalyst (68.4% decrease in current density), and the maximum power density of the catalyst was 172.2 mW cm^−2^ when applied to an actual anion‐exchange membrane fuel cell, thus belonging to a promising eco‐friendly electrocatalyst material.^[^ ^94^ ^]^ 2D porous Fe‐monoatomic catalysts were synthesized using purified porcine blood hemoglobin rich in Fe‐porphyrins by effective pyrolysis to form Fe‐monoatomic active sites anchored in thermally exfoliated graphene oxide. The higher ratio of mesoporous/macroporous porous structure ensures electrode‐electrolyte contact and the high ratio of Fe‐N sites enhances electron donor binding to pyridine nitrogen and graphitic nitrogen groups to promote O_2_ adsorption ultimately resulting in excellent ORR activity. The catalyst exhibited a current density of 548 mA cm^−2^ at 0.6 V and a maximum power density of 409 mW cm^−2^ when applied to AMEFC as shown in Figure 17d, confirming the potential of iron‐based catalysts for commercialization in AEMFCs.^[^ ^515^ ^]^

#### 6.2.3 Microbial Fuel Cells

Microbial fuel cells (MFC) differ from conventional fuel cells in that they use electricity‐producing microorganisms to replace the catalysts in conventional fuel cells, convert the chemical energy of organic matter into usable electricity through their metabolism, and maintain a high conversion rate by steadily outputting electricity to the outside while degrading sewage.^[^ ^516^ ^]^ The configuration of a typical microbial fuel cell is similar to that of a general fuel cell, consisting of an anode chamber and a cathode chamber, with an exchange membrane separating the two chambers, but considering the high impedance caused by the exchange membrane and the negative impact on electron movement, there is also an air‐cathode single‐chamber MFC as shown in Figure 17e.^[^ ^517^ ^]^ Whether it is a dual‐chamber MFC or an air‐cathode single‐chamber MFC, the reaction principle is the same. Microorganisms present on the anode surface are oxidized to CO_2_ and protons through metabolic degradation with reducing organic matter (lactic acid, glucose, and acetate, etc.) under strictly anaerobic or partly anaerobic conditions, and the generated intracellular electrons are transferred to the cathode chamber through an external circuit to produce oxygen ions (O^2−^ or O_2_^2−^) by an ORR with oxygen molecules in solution, and then combined with hydrogen ions in solution to produce water to complete The charge transfer inside the cell is completed. Although MFC has the advantages of a mild operating environment, strong biocompatibility, low energy consumption, and low cost, it still has the disadvantage of low energy output power, limiting its practical application. Among them, the oxygen reduction pathway and reduction efficiency largely determine the power production of MFC, therefore, electrocatalysts need to be introduced to improve the ORR efficiency and thus increase the MFC output power.^[^ ^65^, ^518^, ^519^ ^]^

Doping of heteroatoms into the carbon matrix is an effective strategy to enhance ORR activity, where the heteroatoms disrupt the electroneutrality of the adjacent carbon atoms, creating additional charged sites for the adsorption of reduced oxygen. Heteroatom doping combined with a material structure that allows for easy electron and mass transfer constitutes a catalyst with high electrocatalytic activity. While most biomass materials exhibit defects with self‐doped heteroatoms, inherent characteristics, and favorable structures are ideal carbon precursors for catalysts.^[^ ^520^ ^]^ The self‐doped biomass‐based CAC material synthesized in a controlled manner after extraction of microalgal lipids to a high‐value product (bio‐oil) is characterized by nitrogen enrichment (8.31% increase in nitrogen content) and multilayer graded pore structure. The material was practically applied to the MFC with a maximum power of 412.85 mW m^−2^ and a maximum output voltage of 0.431 V for one full cycle (11.3h) of operation.^[^ ^101^ ^]^ The catalysts prepared after high‐temperature graphitization of watermelon rind reduce the mass transfer resistance, constitute a multilevel porous structure, and expose effective active sites to promote ORR kinetics. And high concentrations of pyridine‐N and graphite‐N were achieved, weakening the O‐O bonding and accelerating the oxygen reduction process, while enhancing the material conductivity. The lower ohmic resistance (Ro) and charge transfer resistance (Rct) of the catalysts are favorable to promote charge transfer and accelerate the ORR reaction interface rate to improve the yield of MFC. The watermelon peel‐derived CAC‐based catalyst in Figure 17f exhibits Ro and Rct of 8.85 and 20.63 Ω, respectively, lower than the Pt/C catalyst (27.19 and 37.56 Ω). This catalyst is a superior alternative to non‐metallic catalysts for microbial fuel cell applications.^[^ ^521^ ^]^ The biomass‐derived CAC catalyst prepared using the leaves of water lilies kept nitrogen‐doped and altered the morphology of the carbon matrix to multi‐layered porous carbon nanosheets ensuring high intrinsic activity, fast electron transfer and mass transfer pathways, and high density of active sites can equally exhibit impressive ORR catalytic performance. Application of this catalyst to MFC showed better current density (2.37 A m^−2^) after five cycles of operation and open circuit voltage (*V*_oc_ = 0.80 V), maximum power density (*P*_max_ = 1122.13 mW m^−2^), and short circuit current density (*I*_sc_ = 4.36 A m^−2^) after 570 h of cycling tests than Pt/C catalyst (current density of 1.22 A m‐2, *V*_oc_ = 0.77 V, *P*_max_ = 430.01 W m^−2^ and *I*_sc_ = 1.18 A m^−2^) as shown in Figure 17g.^[^ ^105^ ^]^

Transition metal compounds loaded on biomass (corn stover) carbon supports have good catalytic performance for ORR, and good Fe_3_Se_4_/FeSe heterojunctions were obtained by embedding Fe_3_Se_4_ into the nitrogen‐doped carbon skeleton to control the growth of bare crystalline surfaces. The presence of heterojunctions provides a large number of active sites that enhance the transfer of electrons and protons, greatly enhancing the interfacial effects during the reaction. Moreover, the embedded structure and the mixed crystalline surface make the catalyst less susceptible to aging during the ORR reaction and improve the mentioned efficiency and power output of the catalyst in MFC. In the actual application to MFC and running 40 cycles in a three‐day cycle, the catalyst was still able to maintain 612.5 mV after 40 cycles, with a decrease of only 2.4%, while Pt/C as the positive electrode had significantly poorer biological corrosion resistance, with performance gradually decreasing to 300 mV in the 5th cycle as shown in Figure 17h. It indicates that the NiAs‐type structure of Fe_3_Se_4_ and the ordered arrangement of iron vacancies in the catalyst help to improve the catalytic activity and microbial resistance, so the electron transfer pathway during ORR is not easily affected by microorganisms.^[^ ^414^ ^]^ Excellent performance can also be achieved by using another form of heteroatom‐doped CAC material anchored to a copper vacancy‐rich substrate to form a new coordination structure. The N and S co‐doped carbon dots prepared from the orange peel as a carbon source are anchored on the copper substrate by electrodeposition, where S and N introduce negative charges in the C lattice leading to lattice disorder, increasing the coordination activity of the copper and increasing the concentration of copper vacancies. The catalyst applied to the MFC cathode exhibited a maximum power density of 924.5 ± 32.5 mW m^−2^, 28.0 times higher than that of the Pt/C cathode (1.34 ± 686.5 mW m^−2^).^[^ ^522^ ^]^

### 6.3 Production of Hydrogen Peroxide

The above discussion about the application of biomass CAC materials to various batteries occurs in the four‐electron ORR pathway, while the two‐electron ORR is mostly used for the electrocatalytic generation of hydrogen peroxide. The electrochemical cathodic ORR method for hydrogen peroxide is a simpler and more efficient, safe, and environmentally friendly method for the preparation of hydrogen peroxide that has been developed in recent years.^[^ ^523^, ^524^, ^525^ ^]^ During the ORR, the selectivity of water generation is much higher than that of hydrogen peroxide generation, and the reaction is thermodynamically more inclined to the four‐electron path, so it is of great importance to developing CAC‐based catalysts with high hydrogen peroxide selectivity, high activity, and high stability. The binding of the intermediate *OOH to the catalyst surface is critical for the selectivity of the ORR pathway. When the adsorption of intermediate *OOH on the catalyst surface is too strong it increases the probability of O‐O bond breakage and further dissociation to generate water, so it is important to design catalysts with weak adsorption of intermediate *OOH, kinetically reduce the barriers of oxygen adsorption and *OOH desorption, and have high oxygen reduction activity while retaining the high selectivity of hydrogen peroxide generation.^[^ ^68^, ^480^, ^526^, ^527^ ^]^

**Figure 18:**
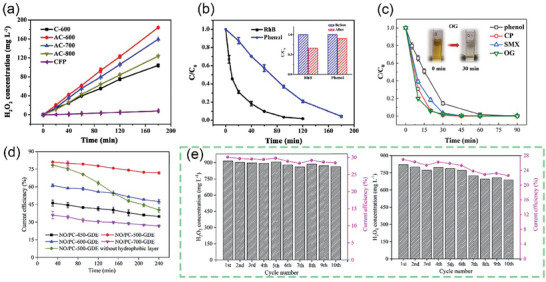
a) Time‐dependent H_2_O_2_ concentrations synthesized at −0.6 V and pH 3 by CAC materials, time‐dependent H_2_O_2_ concentrations synthesized by AC‐600 at different potentials. b) degradation performance of electro‐Fenton system with AC‐600 as the cathode material. The inset in b is the concentration change of organic pollutants during preadoption for 30 min before electro‐Fenton reaction.^[^ ^528^ ^]^ Copyright 2020 Elsevier B.V. All rights reserved. c) Removal of different organic pollutant in ZnBC‐550 based EF system.^[^ ^529^ ^]^ Copyright 2020 Published by Elsevier B.V. d) during the electrolysis by various composite cathodes (NO/PC‐X to PTFE binder ratio (m/v) of 1 : 3, pH 3.0 and 5 mA cm^−2^).^[^ ^116^ ^]^ e) The stability of the NO/PC‐500‐GDE and the restored cathode at 100 mA cm^−2^. (NO/PC‐500‐GDE without a hydrophobic layer electrolyzed at 30 mA cm^−2^ after 240 min electrolysis is defined as the deactivated cathode. The restored cathode was obtained by introducing a hydrophobic layer on the deactivated cathode).^[^ ^116^ ^]^ Copyright 2024 Copyright Clearance Center, Inc. All rights reserved.

The porous CAC material was prepared by high‐temperature carbonization of lignocellulosic Cephalosporium inner shells and acid washing to produce surface mesopores, followed by activation pyrolysis. The two‐step treatment resulted in the formation of large and medium pores on the surface of the material and the further introduction of smaller pores, finally showing a graded porous carbon with large pores and dense small medium pores, ensuring rapid ion/electron transfer. The CAC material was tested at 0.6 V and pH 3.0 with a cumulative H_2_O_2_ concentration of 184 mg L^−1^ for 3 h. The CAC material degraded 98.3% phenol at 120 min and 96.4% RhB (35 mg L^−1^) at 180 min as a cathode material in an electro‐Fenton system with good pollutant degradation performance as seen in **Figure** **18**a,b.^[^ ^528^ ^]^ The graphitization temperature of the cellulose was regulated to alter the pore structure, surface defects, and oxygen functional groups of the final multistage pore carbon, as evidenced by the high temperature decreasing the porosity and specific surface area, forming more graphitic carbon, increasing the degree of intrinsic defects (pentagonal rings and serrated edges), decreasing C─O/C═O ratio in the oxygen groups, and decreasing H_2_O_2_ selectivity despite better ORR activity. The final bio‐derived carbon catalysts prepared by pyrolysis at 550°C had the highest productivity and selectivity and were effectively removed at 90 min for a selection of different contaminants including phenol, chlorophenol (CP), sulfamethoxazole (SMX), and orange G (OG) in the FE system as seen in Figure 18c.^[^ ^529^ ^]^ In Figure 18d, the composite cathode prepared by pyrolysis of alfalfa as raw material and then impregnated with a suitable proportion of PTFE hydrophobic layer is more stable than the catalyst without hydrophobic layer to generate H_2_O_2_ current efficiency, the superhydrophobicity of PTFE coating facilitates the mass movement of O_2_ through the three‐phase interface to the active site and improves the H_2_O_2_ electroproduction, while the absence of hydrophobic layer causes the electrolyte to diffuse into the catalyst pores more easily than O_2_ to accelerate the H_2_O_2_ decomposition. The composite electrode showed only a 51.3 mg L^−1^ decrease in H_2_O_2_ concentration in 10 consecutive experiments (2400 min) of long‐term stability tests as seen in Figure 18e, indicating good H_2_O_2_ generation performance and stability at high current densities, and interestingly, the H_2_O_2_ concentration and current efficiency of the restoration cathode for the deactivated cathode introduced with hydrophobic layer after H_2_O_2_ electrically generated at 100 mA cm^−2^ reached 686.8 mg L^−1^ and 22.6%, respectively, indicating that the hydrophobic layer of the composite cathode produced good restoration performance.^[^ ^116^ ^]^ Ball milling of biomass (peanut shells) to form fine nanoparticles introduces carbon defects as oxygen adsorption sites, and the formation of more oxygen‐containing functional groups by cleaving C═C bonds and promoting oxygen binding during ball milling promotes rapid electrode/electrolyte charge transfer. Thus, surface defects act as oxygen adsorption sites to enhance ORR activity, while oxygen‐containing functional groups promote 2‐electron ORR selectivity. The 2‐h average H_2_O_2_ production rate of ball‐milled CAC materials reached 46.5 mg L^−1^ h^−1^ at pH 7, demonstrating the great potential of ball‐milled biochar for neutral and efficient electrosynthesis of H_2_O_2_.^[^ ^363^ ^]^ Carbon dots were synthesized using biomass‐based materials (glucose) and ionic liquid microwave‐assisted synthesis with a high H_2_O_2_ generation efficiency of 90% in the large potential range of 0.8 V, providing an effective metal‐free catalyst for H_2_O_2_ generation.^[^ ^530^ ^]^

## 7 Summarization and Outlook

### 7.1 Summarization

The amount of green and renewable biomass materials in nature is not less than fossil energy reserves. Therefore, with the shortage of resources caused by the continuous exploitation of fossil energy sources, the abundance of sustainable biomass has become a popular research object to be applied in energy storage and conversion and green chemical technologies. CAC materials prepared from biomass materials become extremely promising candidates for application in electrochemical catalysts based on ORR because of their natural multistage pore structure, high specific surface area, and sufficient electrical conductivity. The CAC‐based catalysts with suitable structures (morphology, pore structure, and surface area) is prepared by the selection of suitable biomass precursors for different reaction media and catalyst applications. This paper reviews the advances in the preparation, the performance enhancement strategies, and the applications of biomass‐derived CAC catalysts based on ORRs in the last 6 years. The following aspects are highlighted:

1. Biomass precursors: The inherent diverse natural structures of biomass materials, the content of internal heteroatoms, and the carbon yield after pyrolysis are all considerations for the preparation of ideal CAC‐based catalysts. The uniformly distributed interconnected hierarchical porous structure and the stable carbon yield inherent in biomass materials provide the basis for the successful preparation of highly active CAC‐based catalysts. Biomass materials with inherently multiple/enriched heteroatoms are a popular choice for specific operating environments/high‐performance requirements. The presence of heteroatoms acts as a major contributor to the formation of active sites. When the physical and chemical properties of the biomass material itself are not satisfactory, the selection of suitable preparation methods, surface and/or structural modification design ideas can turn the biomass material “ from waste to treasure”. Therefore, it is feasible to give electrocatalytic value to biomass‐derived CAC materials.
2. Preparation method: Suitable preparation methods should transform biomass materials into CAC materials with controllable structures and morphologies. The chemical components and pore structure of biomass materials depend on the regulation of experimental parameters and the selection of chemical reagent types and concentrations in the preparation process. The preparation process is a step‐by‐step process of modifying the physical and chemical properties of biomass materials to the desired state by changing various conditions. A simple single synthesis method can produce the target product with low time and energy costs. The combination of different synthesis methods should be used to maximize the value of the biomass material by eliminating the shortcomings of a single preparation method. Regardless of the synthetic method chosen, the resulting CAC‐based catalysts should have a precisely controlled morphology to obtain satisfactory biomass CAC‐based catalysts.
3. Optimization strategies: The CAC‐based catalysts with altered charge distribution and enriched active sites are necessary for practical applications. The starting point for the design of performance optimization strategies (heteroatom doping, defect engineering, single‐atom catalysts, surface molecular functionalization) should revolve around the construction of new electronic structures, the improvement of chemisorption on the catalyst surface, and the exposure of active sites for ORR. The exploration of the regulation mechanism provides a basis for optimizing the catalyst. Remarkably, the catalytic performance obtained by either optimization strategy does not show a purely linear relationship with the active site density. After the successful construction of new active sites, the precise modulation of the active site density is the key to maximize the value of biomass‐derived CAC electrocatalysis.
4. Performance in electrocatalytic applications: When applied to metal‐air batteries and fuel cells, the existence of the porous structure of biomass‐derived CAC materials ensures electrolyte permeation, oxygen and charge transport, and the construction of three‐phase interfaces for high‐speed electrocatalytic reactions. The preparation of hydrogen peroxide by two‐electron oxygen reduction catalysis is a simple and efficient, economical and safe method, and as a competing reaction to the four‐electron ORR, the pore structure and active site construction of the designed and prepared catalysts differ, and the performance indexes focus on high hydrogen peroxide selectivity and hydrogen peroxide yield.

### 7.2 Outlook

The application of biomass‐derived CAC materials in oxygen reduction electrocatalysis has made a lot of progress in recent years. But there are still limitations and challenges for further development as follows:

1. Development of efficient and controllable pyrolysis methods: Traditional pyrolysis methods have high energy consumption and long pyrolysis times. The pyrolysis methods discussed above (microwave heating, laser heating) are less time‐consuming and have low energy consumption, but they are unable to accurately control the rate of pyrolysis and ensure uniform heating. Neither pyrolysis method can create a uniform temperature field based on the texture of the material. The inhomogeneity of the temperature field and the mass and heat transfer of the material make it difficult to ensure that the reactions are synchronized. In order to ensure precise control of the entire pyrolysis process, new pyrolysis methods with high‐energy input and thermal induction devices have been developed, such as the introduction of Joule heating and magnetic induction heating. Real‐time monitoring/regulation of the material as a whole and locally in a homogeneous temperature field is the first step to ensure a high degree of product homogeneity. The development of new pyrolysis technologies is becoming increasingly sophisticated. The industrialization of CAC catalysts based on biomass is imminent.
2. Development and application of green and efficient controlled activation methods: Existing activation methods obtain the desired conformation of the catalyst through pretreatment or post‐treatment to enhance activation. Complex preparation and waste treatment processes are not sufficient for commercial applications. Physical activation is maximized by introducing new reaction devices to increase the physical activation time, accelerate the activation reaction rate, or increase the reactant concentration to generate the desired carbon material configuration or chemistry. In addition, we can continue to explore the development of efficient chemical activators that are not harmful to the environment during the activation process. We can even design experimental protocols for secondary physical activation of biomass materials using gases generated during the activation process. There are still many possibilities for exploring green and efficient activation methods.
3. Precisely controlled optimization: High‐level synthesis of homogeneous and specific types of heteroatom dopants, defects, or specific combinations of both on biomass‐derived CAC materials remains a major challenge. It is even more difficult to precisely regulate the doping and/or defect profiles of CACs at the spatial and density levels. In view of the inherent uncontrollability of biomass materials after carbonylation activation, the feasibility of preparing doped and/or defective CAC materials with specific spatial distributions and reasonable active site densities under different scenarios or different experimental parameters can be simulated by computer to accelerate the process of preparing ideal catalysts. Catalyst design optimization can be advanced by anchoring heteroatoms during the heteroatom doping process or by directly synthesizing ideal types of defects with the help of composites consisting of materials with special configurations and biomass materials.
4. Understanding the mechanism of performance optimization: It is unscientific to arbitrarily choose one or several chemical components/modulation methods to explore the direction/pathway of catalyst performance optimization. Therefore, there is a need to deepen the understanding of the mechanism of maintaining the balance of parameters such as porous structure, specific surface area, active sites, and reaction interfaces under the synergistic effect of carbon network and non‐carbon components to further optimize the catalyst performance. In particular, the synergistic effect of various enhancement strategies can lead to a chaotic state of the regulatory mechanism. Therefore, there is an urgent need to develop precise in situ or operational characterization methods to accurately describe the dynamic changes in fine structure and surface charge state during catalyst synthesis and reaction. Real‐time changes in catalyst structure and/or components during participation in the reaction process provide guidance for precise synthesis as well as for the next step of catalyst optimization. The establishment of new descriptors to evaluate the relationship between specific structures and intrinsic catalytic activity is also necessary. A comprehensive understanding of the reaction mechanism is helpful for rational and controllable design and fabrication of catalysts.
5. Improvement of the feasibility of synthesizing ideal catalysts: Catalyst configurations designed through theoretical model simulations still vary greatly in the actual preparation process. Alternatively, there may be cases where the design solution cannot be implemented on a large scale. Typically, CAC‐based catalysts are prepared from biomass materials as powder or bulk products. The use of 3D printing technology to obtain biomass‐derived CAC materials with tightly controlled pore structures can be challenging to achieve large‐scale/large‐scale structuring of biomass materials or the construction of complex micro‐ and nanostructures. The relationship between the morphology and structure of the material at the molecular level as affected by pyrolysis‐assisted shrinkage leading to molecular morphology and structural transformation is not yet known. It is also valuable to advance biomass‐derived CAC from laboratory scale to process protocols. This is because the entire chain from biomass materials to energy devices should be fully developed to ensure the recycling of materials and reagents as well as to promote green cycle sustainability of electrochemistry.
6. Promotion of the development and application of two‐electron ORRs: Compared with 2e^−^ ORR, the catalyst design and preparation methods as well as the performance evaluation techniques for 4e^−^ ORR are more mature and perfect. Especially, there are not many papers on the use of biomass materials as precursors for 2e^−^ ORR catalysts. Therefore, more attention should be paid to this field to promote its rapid development. In addition, the product of 2e^−^ ORR reaction, H_2_O_2_, is a good oxidizer, which can cause damage to the electrode during the reaction process. Therefore, when designing catalysts and reaction devices, more consideration should be given to prolonging the stability of the catalysts as well as the durability of the electrodes. The preparation of H_2_O_2_ by 2e^−^ ORR electrocatalysis alone would be too monolithic in value. It is important to enrich other chemical values and increase the competitiveness of research and development in this direction by designing oxygen reduction reactions coupled with other oxidation reactions in the cell. In addition, the controllable and convenient use of clean energy (electricity) to catalyze the oxygen reduction of H_2_O_2_ should be exploited for the in‐situ degradation of pollutants or as a feedstock for the in‐situ synthesis of other high‐demand chemical products.
7. Enrichment of optimized ORRs for applications: Biomass‐derived CAC catalysts have better ORR activity under alkaline conditions compared to neutral and acidic media. However, in practical electrochemical applications, electrical energy storage devices are more efficient and stable in acidic media while not affected by CO_2_. Oxygen reduction reactions in acidic media result in increased mass transfer resistance and decreased catalytic activity due to the destruction of the carbon carriers and flooding of the microporous structure. Meanwhile, the detachment of metal sites and the oxidation of carbon by the generated free radicals can also lead to poor catalyst stability. Therefore, the development of more stable acidic electrocatalysts is necessary. The mass transfer efficiency was improved by increasing the degree of graphitisation of the carbon material and the highly hydrophobic carbon surface. Prevent active site detachment by changing the metal site conformation or adding carbon layer protection. Introduction of free radical scavengers in the reaction device or timely separation of the generated free radicals. The current general trend is to synthesize biomass CAC‐based catalysts for all PHs. Catalyst design for other reactions can also be inspired by synthesis/modulation strategies for high‐performance oxygen reduction catalysts. Adaptation to the differences between the different reactions to facilitate the co‐development of each reaction.

## Conflict of Interest

The authors declare no conflict of interest.
